# Polyphenol-Mediated Modulation of Oxidative Stress Pathways in Type 1 Diabetes: A Systematic Review

**DOI:** 10.3390/antiox15060693

**Published:** 2026-05-30

**Authors:** Alan Ho, Harini Adivikolanu, Dilan Patel, Xinyue Wang, Rahul Mittal, Khemraj Hirani

**Affiliations:** 1 Diabetes Research Institute, University of Miami Miller School of Medicine, Miami, FL 33136, USAdilanpatel@med.miami.edu (D.P.);; 2 Division of Endocrinology, Diabetes, and Metabolism, Department of Medicine, Miller School of Medicine, University of Miami, Miami, FL 33136, USA

**Keywords:** type 1 diabetes, oxidative stress, polyphenols, antioxidants, Nrf2 pathway, reactive oxygen species, β-cell dysfunction, lipid peroxidation, redox signaling, preclinical models

## Abstract

Oxidative stress is a central component of type 1 diabetes (T1D) pathophysiology, contributing to pancreatic β-cell vulnerability and the development of chronic complications. Current therapeutic strategies are primarily focused on glycemic control and do not directly address underlying redox imbalance. Dietary polyphenols, a structurally diverse class of plant-derived compounds, have been investigated for their antioxidant and cytoprotective properties, yet their role in T1D has not been systematically defined. This systematic review evaluates the effects of polyphenols on oxidative stress and glycemic parameters in preclinical models of T1D. Across studies, polyphenols were consistently associated with attenuation of oxidative stress, as evidenced by reductions in lipid peroxidation and reactive oxygen and nitrogen species, along with restoration of endogenous antioxidant defenses, including superoxide dismutase, catalase, and glutathione. These effects were frequently linked to modulation of redox-sensitive signaling pathways, particularly Nrf2-dependent mechanisms. In contrast, glycemic outcomes were heterogeneous and influenced by compound-specific and experimental factors. Modulation of oxidative stress markers was often observed independently of changes in glycemic parameters, suggesting a primary redox-mediated mode of action. These findings provide a mechanistic rationale for prioritizing oxidative-stress-focused endpoints in future translational studies and support the evaluation of polyphenols as adjunctive strategies targeting redox imbalance in T1D.

## 1. Introduction

Type 1 diabetes (T1D) is characterized by the progressive autoimmune destruction of pancreatic β-cells, resulting in an absolute deficiency in endogenous insulin production and lifelong dependence on insulin replacement therapy. In contrast to type 2 diabetes (T2D), which is primarily driven by insulin resistance and relative insulin deficiency, T1D arises from a combination of genetic susceptibility and environmental triggers that lead to immune-mediated β-cell damage [1]. Although T1D is commonly diagnosed between ages 10 and 14 years, the median age of diagnosis in the United States is 24 years, reflecting a substantial burden of adult-onset disease [2]. T1D accounts for approximately 5–10% of all diabetes cases globally. However, its prevalence continues to rise, with an estimated 8.4 million individuals affected worldwide in 2021 and projections indicating a 66–116% increase by 2040 [3].

The standard of care for T1D relies on exogenous insulin therapy and continuous glucose monitoring [4]. Although insulin therapy is essential and lifesaving, it has notable limitations, including risks of hypoglycemia, treatment-associated weight gain, and increased cardiovascular risk [4,5]. Importantly, insulin alone does not address key underlying pathophysiological abnormalities in T1D, such as α-cell dysfunction, nor does it fully prevent the development of long-term complications [5]. Achieving and maintaining optimal glycemic control with insulin monotherapy remains challenging, and many patients continue to experience significant glycemic variability [5,6]. As a result, individuals with T1D remain vulnerable to microvascular and macrovascular complications, including diabetic nephropathy, retinopathy, neuropathy, and cardiovascular disease [7]. These limitations have driven interest in adjunctive therapeutic strategies that complement insulin therapy by targeting alternative disease mechanisms.

A defining feature of T1D pathophysiology is the central role of oxidative stress in disease progression. Pancreatic β-cells are particularly vulnerable to oxidative damage due to their relatively low expression of antioxidant defense enzymes. Hyperglycemia promotes oxidative stress through multiple mechanisms, including mitochondrial electron transport chain overload with excess reactive oxygen species (ROS) production, increased polyol pathway flux leading to NADPH depletion and impaired antioxidant defenses, and activation of the hexosamine pathway, which contributes to inflammatory signaling and cellular injury. In addition, hyperglycemia drives the formation of advanced glycation end products (AGEs), further exacerbating oxidative damage to proteins, lipids, and nucleic acids. Collectively, these processes sustain the inflammatory cascade underlying T1D and contribute to the development of microvascular complications, including nephropathy, retinopathy, neuropathy, and cardiovascular dysfunction [8,9]. Key oxidative stress biomarkers, including malondialdehyde (MDA), as well as antioxidant enzyme activities such as superoxide dismutase (SOD), catalase (CAT), and glutathione (GSH), are consistently altered in T1D [10,11].

Dietary polyphenols are a structurally diverse class of plant-derived secondary metabolites abundant in fruits, vegetables, tea, and red wine [12]. They have attracted growing interest as potential adjunctive therapies in diabetes management due to their broad range of biological activities, including antioxidant, anti-inflammatory, hypoglycemic, and cytoprotective effects [13]. Accumulating preclinical and clinical evidence suggests that long-term consumption of polyphenol-rich diets may confer protection against chronic diseases, including diabetes mellitus, cardiovascular disease, and inflammatory disorders [12]. In the context of diabetes, clinical evidence supporting polyphenol efficacy has been largely generated in T2D populations. For example, a meta-analysis of 36 randomized controlled trials reported a modest but significant reduction in HbA1c (−0.21%, *p* < 0.001) with polyphenol supplementation in individuals with T2D [14].

Polyphenols are broadly classified into flavonoids and non-flavonoids. Flavonoids, the largest group, include subclasses such as flavonols (such as quercetin, kaempferol), flavones (such as apigenin), flavan-3-ols (such as catechins, epigallocatechin gallate), flavanones (such as naringenin), and isoflavones (such as genistein) [12,13]. Non-flavonoids include phenolic acids, stilbenes (such as resveratrol), lignans, tannins, and curcuminoids [12,13]. Mechanistically, polyphenols exert their effects through multiple pathways, including direct scavenging of ROS, upregulation of endogenous antioxidant defenses via activation of the Nrf2/Keap1 pathway, and modulation of key enzymes involved in glucose and lipid metabolism [15,16,17,18].

Among individual compounds, resveratrol, quercetin, and curcumin have been the most extensively studied polyphenols in this context. Resveratrol, a stilbene polyphenol found in grapes, peanuts, and berries, has demonstrated reductions in blood glucose and HbA1c in T1D animal models through improved β-cell function, enhanced glucose uptake, and improved insulin action [19]. In a two-month exploratory human trial of thirteen patients, resveratrol supplementation administered adjunctively to insulin therapy significantly improved fasting blood glucose and HbA1c, alongside decreased MDA levels and increased total antioxidant capacity [20]. Quercetin, a structurally related flavonoid widely distributed in fruits and vegetables, has also shown protective effects on pancreatic β-cells in STZ-induced diabetic rat models, including normalization of blood glucose, augmentation of hepatic glycogen content and enzyme activity, improvement in antioxidant status, and promotion of pancreatic islet regeneration [21]. Importantly, co-treatment with quercetin and resveratrol in STZ-induced diabetic rats produced synergistic antidiabetic effects, with the combination demonstrating greater improvements in glycemic control, antioxidant parameters, lipid profiles, and pancreatic β-cell preservation than either compound alone [22]. Curcumin, a diarylheptanoid derived from turmeric, has demonstrated significant immunomodulatory effects in autoimmune diabetes models, with treatment in accelerated NOD mouse models associated with delayed disease onset and, in some cases, prevention of autoimmune diabetes through inhibition of pancreatic leukocyte infiltration and preservation of insulin-expressing cells [23].

**Figure 1:**
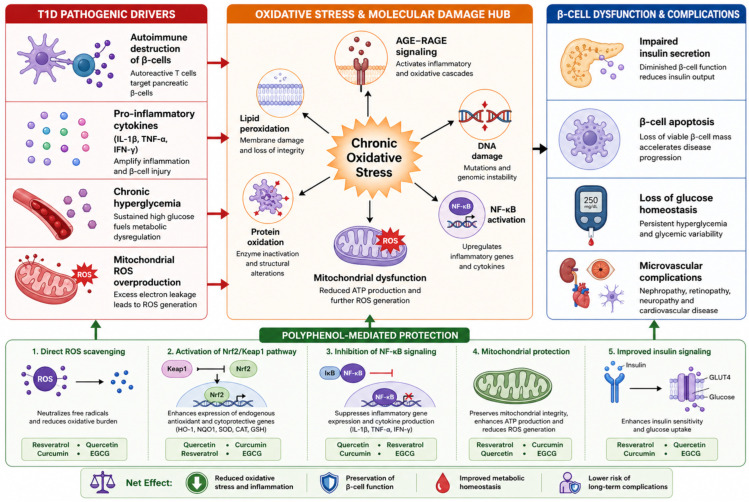
Integrated model of oxidative-stress-mediated β-cell dysfunction in type 1 diabetes and mechanisms of polyphenol-mediated protection. Autoimmune β-cell destruction, proinflammatory cytokines, chronic hyperglycemia, and mitochondrial reactive oxygen species (ROS) overproduction converge to drive chronic oxidative stress. This promotes lipid peroxidation, protein oxidation, DNA damage, NF-κB activation, and mitochondrial dysfunction, leading to impaired insulin secretion, β-cell apoptosis, loss of glucose homeostasis, and microvascular complications. Polyphenols (such as resveratrol, quercetin, curcumin, and epigallocatechin gallate) counter these processes via ROS scavenging, activation of the Nrf2/Keap1 pathway, inhibition of NF-κB signaling, mitochondrial protection, and improved insulin signaling, collectively preserving β-cell function and metabolic homeostasis. Created in BioRender. Mittal, R. (2026) https://BioRender.com/uov2sb8 (accessed on 1 May 2026). Abbreviations: AGE, advanced glycation end product; ATP, adenosine triphosphate; CAT, catalase; EGCG, epigallocatechin gallate; GLUT4, glucose transporter type 4; GSH, reduced glutathione; HO-1, heme oxygenase-1; IL-1β, interleukin-1 beta; IκB, inhibitor of nuclear factor kappa B; Keap1, Kelch-like ECH-associated protein 1; NF-κB, nuclear factor kappa B; NQO1, NAD(P)H quinone dehydrogenase 1; Nrf2, nuclear factor erythroid 2-related factor 2; RAGE, receptor for advanced glycation end products; ROS, reactive oxygen species; SOD, superoxide dismutase; T1D, type 1 diabetes; TNF-α, tumor necrosis factor alpha.

The objective of this systematic review is to comprehensively evaluate the effects of dietary polyphenols on glycemic control and oxidative stress in T1D through a structured synthesis of preclinical evidence. Specifically, it seeks to characterize the consistency of these effects across polyphenol subclasses, delineate underlying mechanisms with emphasis on redox-regulatory pathways, and assess their potential relevance as adjunctive strategies within the current therapeutic framework in preclinical animal models. An integrated overview of the pathways linking T1D pathogenesis, chronic oxidative stress, and polyphenol-mediated protective mechanisms is presented in Figure 1.

This review addresses a critical gap in the current literature by providing a focused and mechanistically grounded synthesis of polyphenol effects specifically in T1D, rather than extrapolating from T2D or mixed populations. In contrast to prior work, which has largely evaluated glycemic outcomes in isolation or aggregated heterogeneous diabetic cohorts, this study systematically integrates glycemic and oxidative stress endpoints across defined polyphenol subclasses and experimental models. By organizing evidence along both chemical class and biological mechanism, this review advances beyond compound-specific observations to delineate convergent redox-regulatory pathways that are directly relevant to T1D pathophysiology. This approach enables a more precise assessment of where polyphenols exert their most consistent effects and clarifies their potential role within a disease framework characterized by oxidative vulnerability. As such, this work provides a structured foundation for future translational studies and offers a more targeted perspective on the therapeutic relevance of polyphenols in T1D.

## 2. Methods

This systematic review aimed to comprehensively evaluate the available evidence from animal studies examining the effects of dietary polyphenols on oxidative stress markers and glycemic control parameters in T1D. The study was conducted and reported in accordance with the Preferred Reporting Items for Systematic Reviews and Meta-Analyses (PRISMA) guidelines. The review protocol was registered on the INPLASY database prior to data extraction (registration number: INPLASY202640104).

### 2.1. Search Strategy

A systematic search was conducted across databases PubMed/MEDLINE, Scopus, and Embase. Keywords and Medical Subject Heading (MeSH) terms relating to the intervention (“polyphenols,” “flavonoids,” “resveratrol,” “quercetin,” “curcumin,” “epigallocatechin gallate,” “phenolic compounds”), the population (“type 1 diabetes,” “T1D”), and the outcomes (“glycemic control,” “glucose,” “insulin sensitivity,” “HbA1c,” “oxidative stress,” “antioxidants,” “reactive oxygen species”) were combined using Boolean operators.

### 2.2. Eligibility Criteria

Studies were included if they involved animal models of T1D, evaluated a defined dietary polyphenol or phenolic compound as the primary intervention, included an appropriate comparator (untreated, vehicle, or placebo control), and reported at least one outcome related to glycemic control or oxidative stress. Eligible animal models included streptozotocin (STZ)-induced, alloxan-induced, cyclophosphamide-induced, and spontaneous autoimmune models such as the non-obese diabetic (NOD) mouse. Polyphenol interventions encompassed major phenolic subclasses, including stilbenes, flavonols, flavanols, flavanones, flavones, isoflavones, diarylheptanoids, glycosylated flavonoids, ellagitannins, and standardized plant extracts with identified polyphenol content.

Glycemic outcomes such as fasting blood glucose, insulin, HbA1c, C-peptide, and homeostatic model assessment (HOMA) indices and oxidative stress outcomes such as lipid peroxidation markers, reactive oxygen and nitrogen species, DNA damage markers, antioxidant enzyme activity, non-enzymatic antioxidants, total antioxidant capacity, and upstream pathway markers were considered relevant. Studies published between 1 January 2000 and 31 December 2025 with full text available in English were included. Controlled in vivo animal experiments, ex vivo functional physiology assessments on tissues from in-vivo-treated animals, randomized controlled trials, and prospective cohort studies were all considered eligible study designs.

Studies were excluded if they focused exclusively on T2D, gestational diabetes, or prediabetes without extractable T1D data. Reviews, editorials, commentaries, letters, case reports, and case series were excluded, as were studies without a confirmed T1D model or diagnosis or with absence of a comparator group, pediatric populations, and in-vitro-only designs.

### 2.3. Study Screening and Inclusion

Study selection was conducted in two sequential stages: title/abstract screening followed by full-text assessment. Four independent reviewers (AH, HA, DP, and SW) screened 447 unique records, with each record evaluated against the predefined eligibility criteria. Inter-rater reliability was assessed at each stage using Cohen’s kappa (κ), interpreted according to established benchmarks: <0.00 no agreement, 0.00–0.20 slight, 0.21–0.40 fair, 0.41–0.60 moderate, 0.61–0.80 substantial, and 0.81–1.00 almost perfect agreement. Discrepancies at either stage were resolved through discussion or, where necessary, by the senior reviewers (RM and KH).

### 2.4. Data Extraction

Data from included studies were extracted systematically using a structured form to ensure consistency and completeness. Extraction was performed independently by four reviewers (AH, HA, DP, and SW), with discrepancies resolved through consensus or consultation with the senior reviewers (RM and KH). For each study, general characteristics were recorded, including first author, year of publication, journal, country of origin, and full citation. Study design was categorized basedon the primary class of polyphenol investigated. Intervention characteristics included the polyphenol compound and chemical class, dosing regimen, route of administration (such as oral, intraperitoneal, subcutaneous), and duration of exposure. Population characteristics included species and strain, group sizes, sex distribution, and method of T1D confirmation (such as STZ, alloxan, cyclophosphamide, or NOD model).

Outcomes were extracted and categorized into glycemic control and oxidative stress domains. Glycemic outcomes included established measures of glucose metabolism (such as blood glucose, insulin, HbA1c, C-peptide), while oxidative stress outcomes included markers of oxidative damage, reactive species, antioxidant defenses, and related signaling pathways. For each outcome, the direction and a narrative summary of findings were recorded. Secondary outcomes included lipid profiles, renal function markers, inflammatory markers, vascular function measures, histopathological findings, apoptosis markers, and organ-specific functional outcomes.

Where multiple doses of a polyphenol were evaluated, each dose group was extracted as a separate observation. Funding sources and conflicts of interest were also documented. This structured extraction approach enabled thorough evaluation of study characteristics and outcomes.

### 2.5. Risk of Bias and Quality Assessment

The methodological quality of all included preclinical studies was assessed using SYRCLE’s Risk of Bias tool for animal studies [24]. This tool evaluates ten domains spanning six bias categories: selection bias (D1–D3), performance bias (D4–D5), detection bias (D6–D7), attrition bias (D8), reporting bias (D9), and other sources of bias (D10). Each domain was independently assessed by two reviewers (DP and SW) and rated as Low, Unclear, or High risk of bias. Discrepancies were resolved through discussion and consensus or discussion with the senior authors (RM and KH).

**Figure 4:**
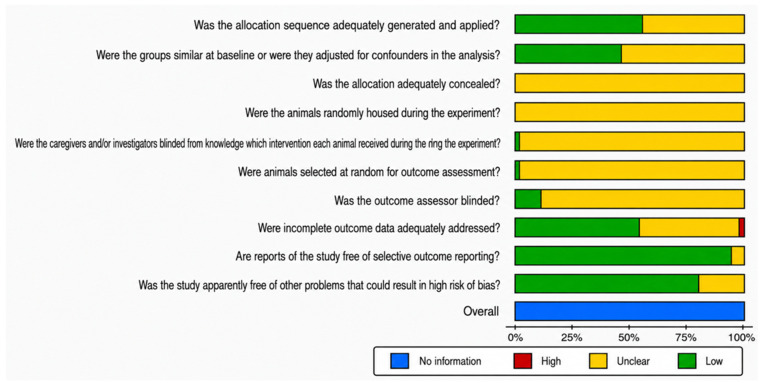
Summary bar chart of risk-of-bias assessment across SYRCLE domains for all 197 included preclinical studies. Each horizontal bar represents one SYRCLE domain (D1–D10) and shows the proportion of studies rated Low risk (green), Unclear (yellow), or High risk (red) for that domain. Domain labels correspond to: D1, sequence generation; D2, baseline comparability; D3, allocation concealment; D4, random housing; D5, caregiver/investigator blinding; D6, random outcome assessment; D7, outcome assessor blinding; D8, incomplete outcome data; D9, selective outcome reporting; D10, other sources of bias.

**Figure 5:**
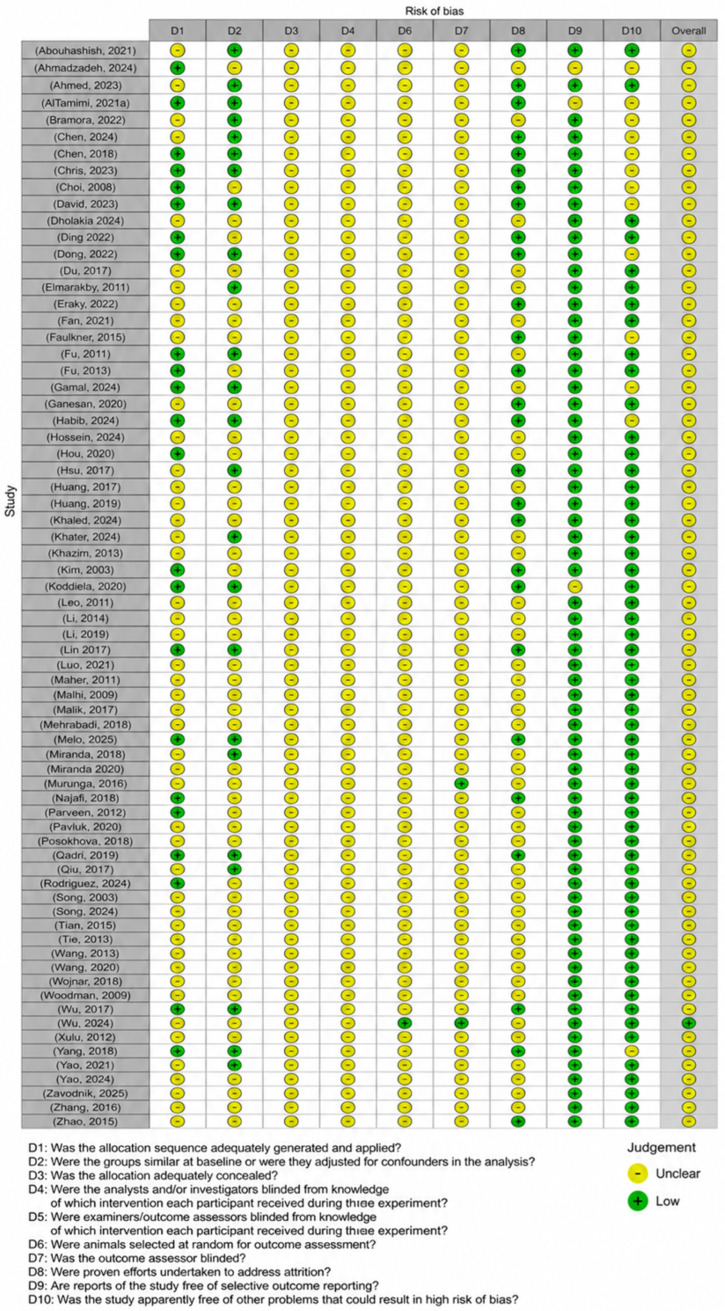
Traffic-light risk-of-bias plot for Flavonoid studies (*n* = 70). Each row represents one included study and each column represents one SYRCLE domain (D1–D10). Individual cells are colored green (Low risk), yellow (Unclear) to indicate the domain-level risk-of-bias judgment for that study.

**Figure 6:**
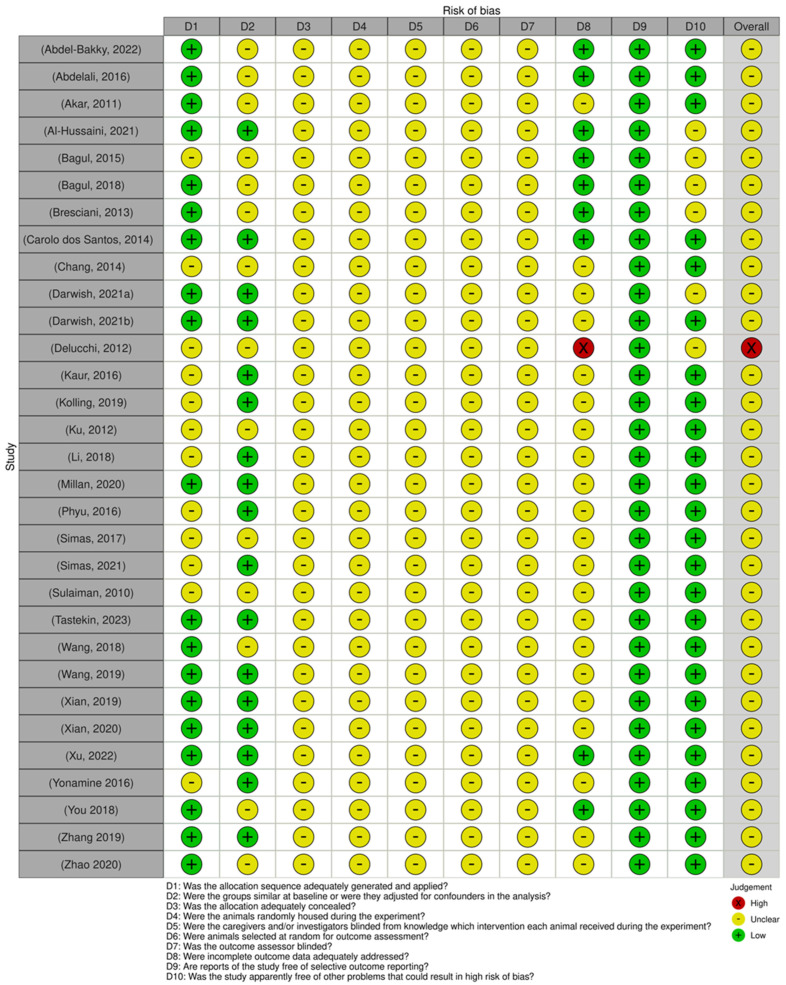
Traffic-light risk-of-bias plot for Stilbene studies (*n* = 31). Each row represents one included study and each column represents one SYRCLE domain (D1–D10). Individual cells are colored green (Low risk), yellow (Unclear), or red (High risk) to indicate the domain-level risk-of-bias judgment for that study.

**Figure 7:**
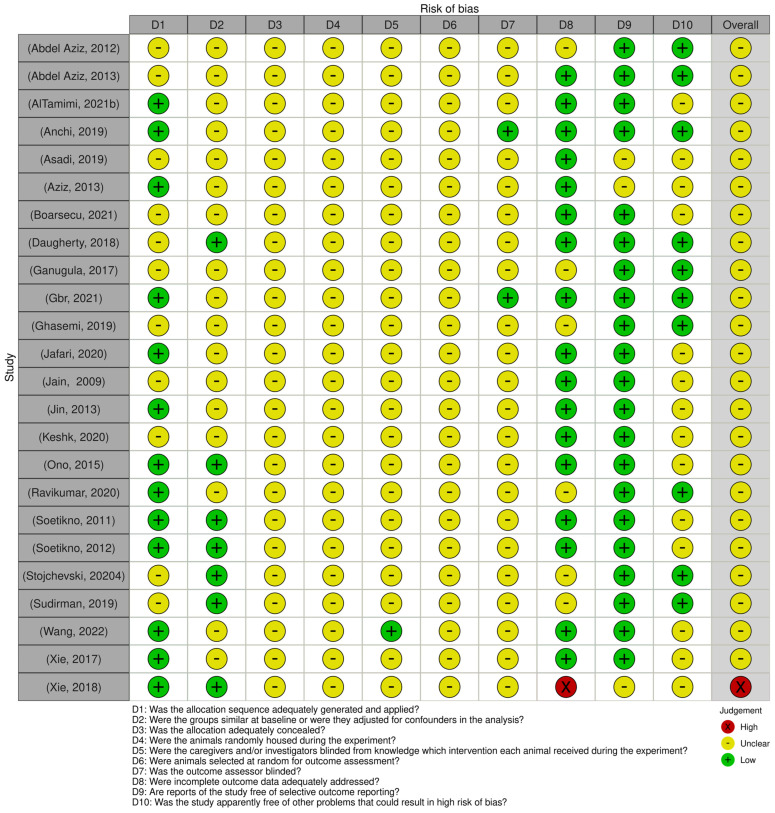
Traffic-light risk-of-bias plot for Diarylheptanoid studies (*n* = 24). Each row represents one included study and each column represents one SYRCLE domain (D1–D10). Individual cells are colored green (Low risk), yellow (Unclear), or red (High risk) to indicate the domain-level risk-of-bias judgment for that study.

**Figure 8:**
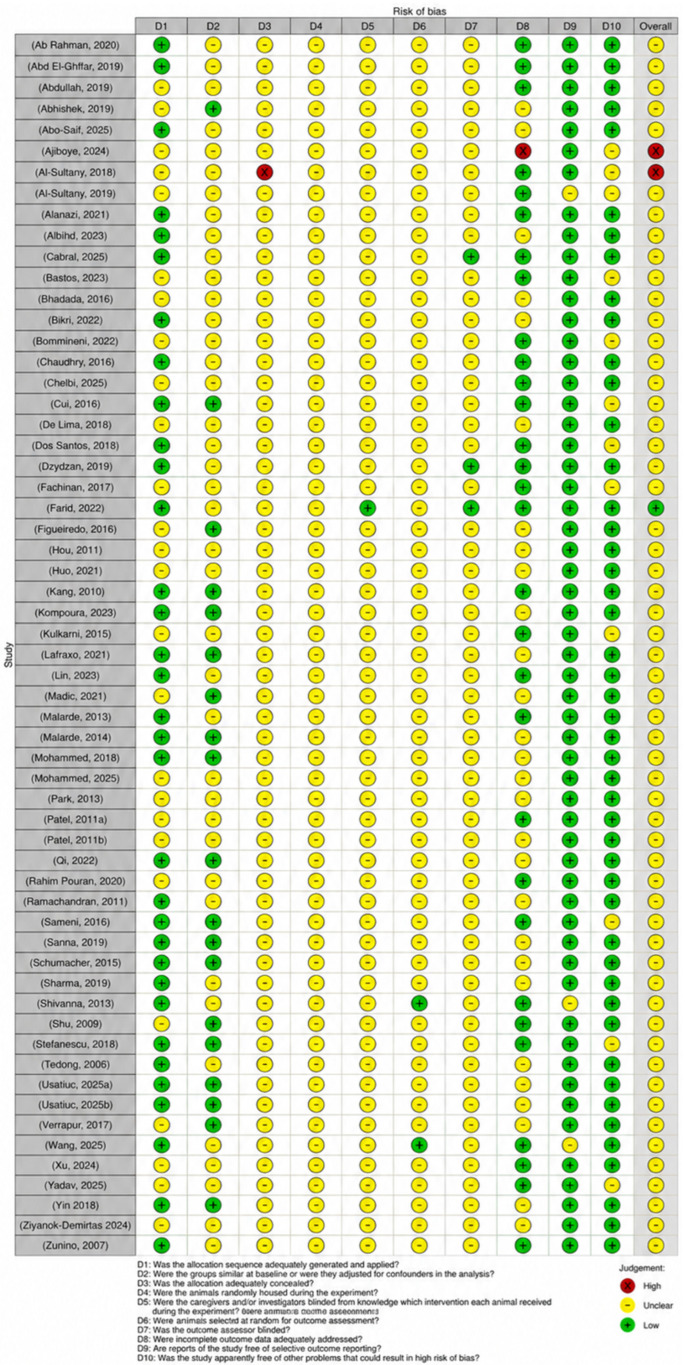
Traffic-light risk-of-bias plot for Natural Extract studies (*n* = 59). Each row represents one included study and each column represents one SYRCLE domain (D1–D10). Individual cells are colored green (Low risk), yellow (Unclear), or red (High risk) to indicate the domain-level risk-of-bias judgment for that study.

**Figure 9:**
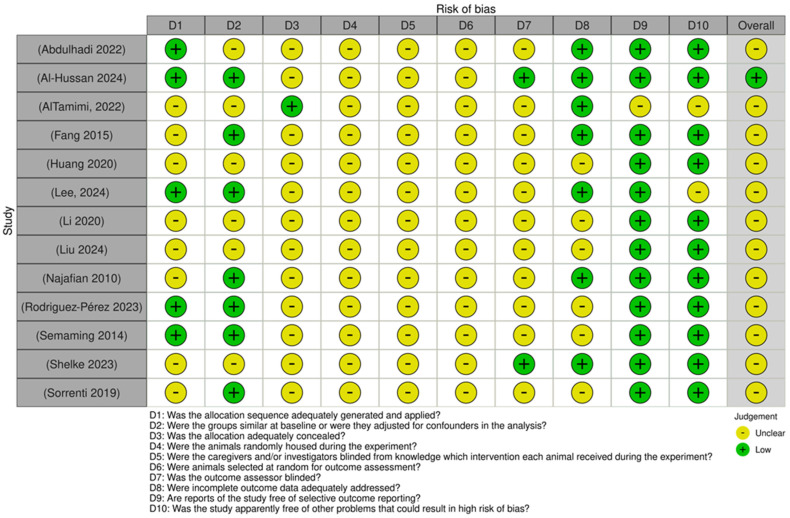
Traffic-light risk-of-bias plot for Other Polyphenol studies (*n* = 13). Each row represents one included study and each column represents one SYRCLE domain (D1–D10). Individual cells are colored green (Low risk), yellow (Unclear) to indicate the domain-level risk-of-bias judgment for that study.

Consistent with the established limitations of SYRCLE in the preclinical literature, domains D3 through D7 were anticipated to yield structurally high rates of Unclear ratings across all studies due to systematic underreporting of allocation concealment, housing randomization, and blinding procedures in animal intervention studies; Unclear ratings in these domains therefore reflect reporting insufficiency rather than confirmed bias. This nonetheless limits the degree of confidence that can be placed in the internal validity of individual studies, and findings should be interpreted with this caveat in mind when considering translational implications. Given the large number of included studies (*n* = 197) and the hierarchical organization of this review by polyphenol class, domain-level risk of bias results are presented as weighted summary bar charts for the full set of papers and as individual traffic-light plots for each polyphenol class (flavonoids, stilbenoids, diarylheptanoids, natural extracts, and other polyphenols) in Figure 4, Figure 5, Figure 6, Figure 7, Figure 8 and Figure 9.

### 2.6. Data Synthesis

The included studies exhibited substantial clinical and methodological heterogeneity, including variation in polyphenol compounds and chemical classes, animal species (such as Wistar and Sprague Dawley rats, C57BL/6 and NOD mice), T1D induction methods (STZ, alloxan, cyclophosphamide, and spontaneous NOD autoimmunity), dosing regimens and routes of administration, and intervention durations ranging from one to twelve weeks. Therefore, a narrative synthesis was adopted as the primary analytic approach. Baseline study characteristics are reported, with sample sizes being aggregated and averaged within each study. To facilitate narrative synthesis, studies were partitioned post hoc by the primary polyphenol class of the intervention, which included the following groups: flavonoids, stilbenes, diarylheptanoids, natural extracts/multi-polyphenol intervention, and other polyphenols. The designations of complete improvement, partial improvement, no improvement for both glycemic control and oxidative stress markers were assigned based on whether or not a significant improvement was observed in all reported outcomes in that paper.

## 3. Results

### 3.1. Study Selection and Characteristics

**Figure 2:**
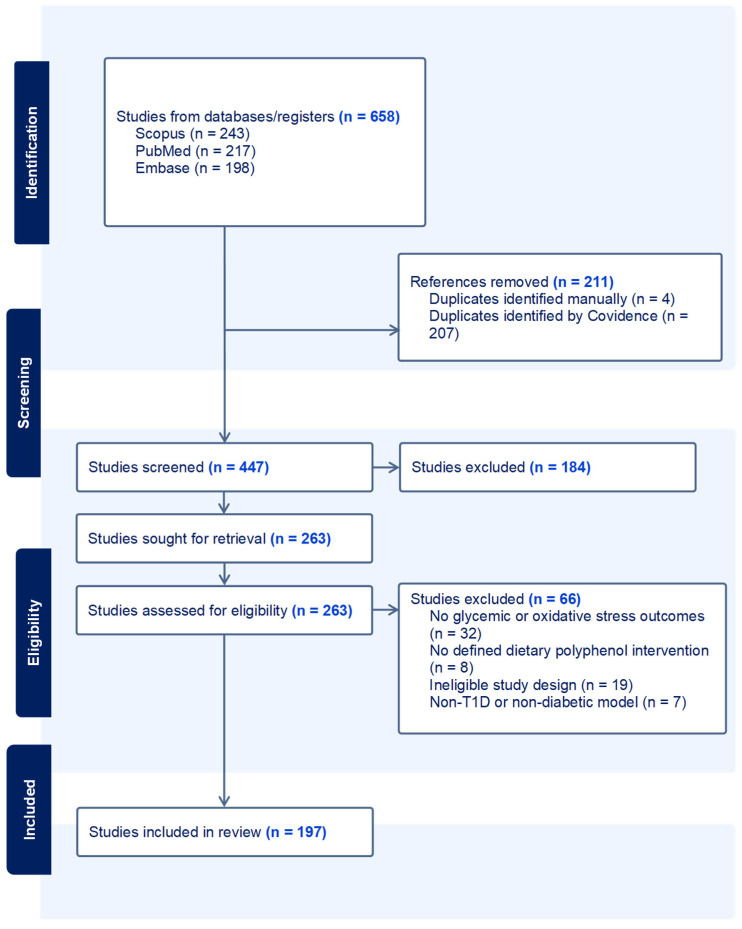
PRISMA flow diagram of study selection. Flowchart showing the systematic identification, screening, eligibility assessment, and inclusion of studies in accordance with Preferred Reporting Items for Systematic Reviews and Meta-Analyses (PRISMA) guidelines.

A systematic search of three electronic databases (PubMed/MEDLINE, Scopus, and Embase) identified a combined total of 658 records published between 1 January 2000, and 31 December 2025. Following removal of 211 duplicate records, 447 unique citations were retained for title and abstract screening. Of these, 184 were excluded based on the prespecified eligibility criteria, and 263 full-text articles were retrieved for detailed assessment. After full-text review, 66 articles were excluded for the following reasons: no glycemic or oxidative stress outcomes (*n* = 32), no defined dietary polyphenol intervention (*n* = 8), review/editorial/case report/in-vitro-only study (*n* = 19), and non-T1D or non-diabetic population/model (*n* = 7). A total of 197 studies met all inclusion criteria and were included in the final qualitative synthesis. A PRISMA flow diagram summarizing the selection process is presented in Figure 2.

### 3.2. Study Characteristics

**Figure 3:**
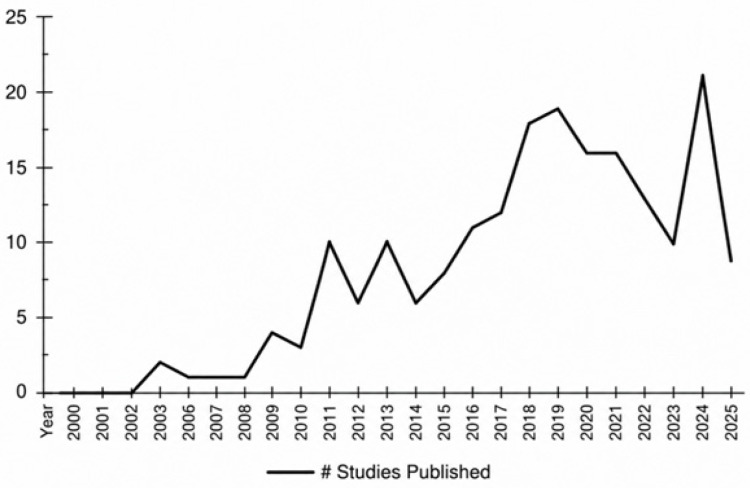
Studies published by year. Number of included studies published from 2000 to 2025.

The 197 included studies were published between 2003 and 2025, with the majority published after 2015 (*n* = 145, 73.23% of studies, Figure 3). Studies originated from 37 countries, with the largest proportions contributed by China (*n* = 47, 23.86%), India (*n* = 24, 12.18%), and Egypt and the USA (*n* = 15, 7.61% for both). The streptozotocin (STZ)-induced rodent model was the most frequently employed method of T1D induction, used in 164 studies (83.25%); alloxan-induced models were second, including both rodents (*n* = 15) and rabbits (*n* = 3) and totaling 18 studies (9.14%); non-obese diabetic (NOD) mouse models were third, used in 12 studies (6.09%). Other methods of induction included cyclophosphamide (*n* = 1) and genetic models of T1D (*n* = 2). The most frequent animal species utilized included Wistar rats (*n* = 80, 40.61%), Sprague Dawley rats (*n* = 47, 23.86%) and C57BL/6 mice (*n* = 29, 14.72%). The only non-rodent species were rabbits, used in three studies (1.52%). The remaining 38 studies utilized various other species of mice and rats (such as Long–Evans rats and Swiss Webster mice). Sample sizes per group ranged from 5 to 66, with a median sample size of 10 (IQR: 7.62–14.63) with three studies not reporting any sample sizes. The most frequent category for route of administration of the polyphenol was oral/oral gavage (*n* = 147, 74.62%), followed by intraperitoneal injection (*n* = 27, 13.71%) and gastric gavage (*n* = 12, 6.09%). The remaining 11 studies used subcutaneous injection, inhalation, or combinations of administration methods.

Inter-rater reliability was evaluated using Cohen’s kappa (κ) statistics to quantify the level of agreement between reviewers at each stage of study selection. The analysis demonstrated substantial agreement in both the title and abstract screening (κ = 0.740) and full-text screening (κ = 0.862), indicating a high degree of consistency and methodological rigor in the screening process.

The largest class of polyphenols were flavonoids (*n* = 70, 35.53%), which were further subdivided into the following groups: flavonols (*n* = 30, 42.86% of flavonoids), isoflavones (*n* = 12, 17.14%), flavanols (*n* = 11, 15.71%), flavones (*n* = 10, 14.29%), and flavanones (*n* = 7, 10.00%). The next highest represented class was that of natural extracts (*n* = 59, 29.25%), broken down into flavonoid-rich plant extracts (*n* = 20, 33.89% of natural extracts), fruit/berry extracts (*n* = 12, 20.34%), phenolic acid/tannin-rich extracts (*n* = 12, 20.34%) and other extracts (*n* = 15, 25.42%). Subsequent classes included stilbenes (*n* = 31, 15.74%) and diarylheptanoids (*n* = 24, 12.18%) which did not have any further subdivisions.

Primary outcomes assessed across all studies included markers of glycemic control (most frequently fasting or random blood glucose, serum insulin and oral glucose tolerance test area under the curve) and oxidative stress endpoints (including malondialdehyde (MDA), superoxide dismutase (SOD), catalase (CAT), and reduced glutathione (GSH)). A summary of the included studies is shown in Supplementary Tables S1–S5.

To facilitate cross-class comparison and improve the utility of this review as a reference resource, we additionally generated a concise mechanistic summary table mapping each major polyphenol class or subclass to representative compounds, dominant reported mechanisms, key formulation or bioavailability considerations, and the relative consistency of glycemic and antioxidant outcomes (Table 1).

## 4. Risk of Bias Assessment

The risk of bias of all 197 included preclinical studies was assessed across ten domains using SYRCLE’s Risk of Bias tool. As anticipated for a preclinical nature of this study, domains D3 through D7, covering allocation concealment (D3), random housing (D4), caregiver and investigator blinding (D5), random outcome assessment selection (D6), and outcome assessor blinding (D7), demonstrated near-universal Unclear ratings (93–100% across all studies), with D4 rated Unclear in 100% of studies, D3 and D6 in 99%, D5 in 98% and D7 in 93%. This reflects the well-documented failure of animal study reporting to describe these procedural elements rather than confirmed methodological deficiency. No High-risk ratings were recorded in any of these five domains across any polyphenol class.

The most informative domains for quality differentiation were D1 (sequence generation), D2 (baseline comparability), D8 (incomplete outcome data), D9 (selective outcome reporting), and D10 (other bias). Across all 197 studies, D9 was the strongest indicator of reporting integrity, with 95% of studies rated Low risk for selective outcome reporting. D10 was similarly reassuring, with 76% of studies rated Low. D1 and D2 showed greater variability, with Low-risk ratings in 49% and 41% of studies respectively, indicating that explicit reporting of randomization procedures and baseline group comparability represents a meaningful gap in this corpus. D8 showed Low risk in 45% of studies, with three studies rated High risk for incomplete data reporting. The summary of assessment proportions for each domain is shown in Figure 4.

Subclass-level analysis revealed broadly consistent patterns across polyphenol classes, with notable differences in the informative domains. The flavonoid subclass (*n* = 70) showed D9 Low in 96% of studies and D1 Low in 39%, consistent with the full corpus average. The stilbenoid subclass (*n* = 31) demonstrated the strongest overall reporting quality among the informative domains, with D9 rated Low in 100% of studies, D2 Low in 55%, and D1 Low in 61%, the highest rate across all subclasses. The diarylheptanoid subclass (*n* = 24) demonstrated the lowest D9 Low rate (88%) and the lowest D10 Low rate (42%) of any class, with 58% of studies rated Unclear for other sources of bias, the highest Unclear rate for D10 across all classes. D2 was also lowest among all classes at 29%, indicating that the baseline group comparability was least frequently documented in this subclass. The natural extract subclass (*n* = 59) showed a D2 Low rate of 32% and D8 Low in 47% of studies. The other polyphenol subclass (*n* = 13) performed favorably on D9 (92% Low) and D10 (85% Low), with D8 Low in 54% of studies. Domain-level traffic-light plots for each polyphenol class are presented in Figure 5, Figure 6, Figure 7, Figure 8 and Figure 9.

### 4.1. Flavonoids

Flavonoids represent the largest and most structurally diverse subclass of polyphenolic compounds investigated in the present review. All flavonoids share a common diphenylpropane carbon skeleton (C6–C3–C6), in which two phenolic rings are linked through a central heterocyclic ring, with the structural diversity of individual subclasses arising from variations in hydroxylation pattern and oxidation state of this central pyran ring. Flavonoids are therefore then conventionally classified into five major subclasses: flavonols, isoflavones, flavanols, flavones, and flavanones. Each subclass exhibits distinct physicochemical properties, dietary sources, and biological activities [25]. The pharmacological relevance of flavonoids in the context of T1D derives from a convergence of complementary mechanisms. Free radicals and oxidative stress are among the primary mediators of autoimmune β-cell destruction in T1D, and the resultant chronic oxidative burden is not fully corrected by exogenous insulin therapy alone [26]. Oxidative stress activates proinflammatory transcription factors such as NF-κB and mediates cytokine release, which recruits and activates immune cells to further amplify ROS generation, sustaining a cytotoxic inflammatory environment that accelerates β-cell destruction [27].

Flavonoids are positioned to interrupt this pathological cycle through multiple converging mechanisms: direct radical scavenging, upregulation of endogenous antioxidant defenses via the Nrf2/Keap1/ARE pathway, and suppression of downstream inflammatory signaling. Preclinical studies indicate that polyphenols with Nrf2-activating capacity reduce ROS, improve insulin sensitivity, and attenuate inflammation in diabetic models, with these effects observed across multiple flavonoid subclasses. It has been established across various animal models that Nrf2 activation provides cytoprotection, ameliorates oxidative stress and inflammation, and delays the progression of diabetes and its associated complications [28]. The findings from each flavonoid subclass are synthesized in the sections that follow.

### 4.2. Flavonols

Flavonols are structurally defined by a hydroxyl group at position C3, a double bond between C2 and C3, and a carbonyl group at C4 of the central heterocyclic ring, features that confer high radical-scavenging capacity and facilitate binding to key redox-regulatory proteins [25]. This group constitutes the largest and most extensively investigated subclass within the flavonoid class in this review, represented by 30 preclinical studies examining 10 distinct compounds: quercetin (*n* = 12), fisetin (*n* = 3), icariin (*n* = 3), kaempferol (*n* = 3), DiOHF (*n* = 2), rutin (*n* = 2), troxerutin (*n* = 2), and astilbin, icariside II, and morin (each *n* = 1).

In terms of T1D induction methods, STZ-induced was the most common with 26 papers, which involved Sprague Dawley rats (*n* = 11), Wistar rats (*n* = 10), and C57BL/6 mice (*n* = 4), followed by alloxan-induced in two papers utilizing Fischer (*n* = 1) and Wistar rats (*n* = 1), and single instances of NOD mice and a genetic model of T1D in Akita mice. Quercetin, the most studied flavonol in this review, is a dietary compound found abundantly in onions, apples, and capers and has been extensively characterized for its ability to activate the Nrf2/Keap1 pathway and suppress NF-κB-mediated inflammatory signaling [29].

**Glycemic Control:** Glycemic outcomes were reported in 29 of the 30 flavonol studies. Results were heterogeneous across compounds, with consistent glycemic efficacy observed for some agents and an absence of glycemic effect for others, suggesting compound-specific rather than subclass-wide effects on glucose homeostasis. Quercetin demonstrated the most consistent glycemic activity. Across 11 of the 12 quercetin studies reporting glycemic outcomes, nine documented significant reductions in blood glucose (81.82%), with several additionally demonstrating improvements in serum insulin, HbA1c, and C-peptide. Specifically, quercetin significantly decreased fasting blood glucose and increased serum insulin in STZ-induced Sprague Dawley rats at doses ranging from 10 to 100 mg/kg/day across multiple independent studies, with lipid-nanoparticle-encapsulated quercetin (QU-Lip) further demonstrating significant reductions in fasting blood glucose alongside restoration of serum insulin and C-peptide, suggesting preservation of functional β-cell mass [30,31,32,33,34,35,36]. In addition, swimming training plus quercetin showed significant glucose reduction in both sedentary and trained diabetic groups, indicating that quercetin’s glycemic effects were independent of physical activity status in this model [37]. The cardiointervalogram study found that both water-soluble and liposomal quercetin forms significantly reduced serum glucose and HbA1c at 10 mg/kg/day over 14 days and additionally reported significant improvements in cardiac autonomic indices and swimming endurance [38]. Two of the quercetin studies (18.18%), one examining lens epithelial–mesenchymal transition and another investigating seminal vesicle oxidative damage, reported no significant glycemic effect at their respective doses [39,40].

Rutin significantly reduced fasting blood glucose in both studies in which it was examined, with one study additionally documenting significant reduction in urinary glucose [41,42]. Kaempferol produced significant glycemic improvement in two of three studies, with doses at 10 mg/kg/day [43] and 50 mg/kg/day [44], while the third kaempferol study, targeting cardiac injury at 10 mg/kg over 8 weeks, observed no significant effect on fasting blood glucose [45].

Fisetin achieved glycemic improvement in one of three studies. The cardiomyopathy study reported significant reductions in fasting plasma glucose, HOMA-IR, oral glucose tolerance test (OGTT) AUC, and intraperitoneal insulin tolerance test (IPITT) AUC at 2.5 mg/kg/day over 8 weeks [46], while the remaining two fisetin studies, conducted in C57BL/6J and Akita mice respectively, found no significant effect on blood glucose or HbA1c [47,48]. Morin (30 mg/kg/day, 5 weeks, Wistar rats) significantly reduced blood glucose levels and restored serum insulin, C-peptide, and IGF-1 concentrations [49]. Astilbin, administered to NOD mice at 50–100 mg/kg twice weekly over 7 weeks, significantly controlled blood glucose trajectories over the observation period [50]. Troxerutin demonstrated mixed glycemic results: the testicular function study reported significant blood glucose lowering at 150 mg/kg/day over 4 weeks [51], whereas the ischemia–reperfusion cardiac study at the same dose and duration observed no significant glycemic effect [52]. Dihydroxyflavonol (DiOHF) similarly demonstrated mixed results, with 5 mg/kg/day revealing no significant blood glucose improvement while 1 mg/kg/day significantly decreased both blood glucose and HbA1C [53,54]. All three icariin studies, conducted in Sprague Dawley rats at doses of 20–80 mg/kg/day over 9 weeks, consistently reported no significant effect on fasting blood glucose or insulin sensitivity index, a finding echoed by the single icariside II study, which similarly observed no significant change in blood glucose or HbA1c at 5 mg/kg/day over 8 weeks [55,56,57,58].

**Oxidative Stress:** The antioxidant profile of flavonols was substantially more consistent than their glycemic effects. Of the 30 studies, 23 reported dedicated oxidative stress outcomes; of these, 19 (82.61%) demonstrated full significant improvement in all assessed markers, three (13.04%) showed partial improvement, and only one (4.3%) showed no significant effect. This dissociation between glycemic and antioxidant outcomes was most clearly illustrated by the icariin series, in which all three studies uniformly reported significant attenuation of oxidative stress, including reductions in MDA and superoxide anion levels and upregulation of Nrf2 nuclear translocation, HO-1, NQO1, SOD2, and Trx1, despite a complete absence of glycemic effect [55,56,57]. Icariside II similarly reduced renal MDA in the absence of any glycemic improvement [58].

Quercetin demonstrated the broadest and most consistent antioxidant activity across the greatest diversity of target organs. Studies documented significant reductions in MDA and restoration of SOD, CAT, GPx, and GSH in renal [30,35], hepatic [34], pancreatic [32], and lens tissues [40]. Additionally, quercetin-mediated antioxidant effects were mechanistically linked to Nrf2 nuclear translocation and upregulation of downstream targets, including HO-1 and SOD1 in renal tissue [59], and to Nrf2 activation in seminal vesicle tissue [39], consistent with its established pharmacology [29]. Quercetin also significantly reduced hepatic superoxide (O_2_•^−^) and H_2_O_2_ in a dose-dependent manner at 50 and 100 mg/kg [34]. Notably, oxidative stress outcomes were not reported in four quercetin studies: those evaluating glycemic and cardiovascular autonomic function [38], vascular endothelial relaxation [37], pancreatic islet dipeptidyl peptidase-4 (DPP-4) inhibitor combination effects [33], and the quercetin formulation comparison [36], reflecting a predominantly secondary-outcome focus in those investigations.

Fisetin demonstrated significant antioxidant improvement across all three studies despite inconsistent glycemic effects, with improvements in key oxidative stress markers such as MDA, ROS, thiobarbituric acid reactive substances (TBARSs) and catalase activity in organs such as nerve root ganglia, the kidney and the left ventricle [46,47,48]. Kaempferol significantly reduced cardiac ROS generation and 3-nitrotyrosine content and upregulated Nrf2/NQO-1 expression in a hyperglycemia-induced cardiac injury model and significantly reduced pancreatic ROS in a β-cell protection study [44,45]; oxidative stress was not assessed in the kaempferol nephropathy study [43]. Morin at 30 mg/kg/day significantly restored SOD, CAT, and GST activity, though GPx was not significantly restored at the lower dose [49]. Astilbin produced a notably context-specific oxidative stress profile: it significantly increased cytoplasmic ROS in CD4+ T-cells while mitochondrial ROS and mitochondrial mass were unchanged, representing the only instance in this review where a flavonol was deployed to modulate rather than uniformly suppress ROS [50]. Troxerutin produced discordant antioxidant results across its two studies: the cardiac study demonstrated significant reductions in myocardial IL-1β and TNF-α, operationalized here as inflammatory–oxidative stress markers [52], while the testicular study observed no significant change in MDA, GPx, or SOD despite significant glycemic improvement [51]. Both DiOHF studies demonstrated significant superoxide attenuation: aortic superoxide generation was significantly reduced in diabetic rats at 5 mg/kg/day and superoxide levels in mesenteric arteries were significantly attenuated at 1 mg/kg/day, though neither dose affected blood glucose [53,54]. Rutin did not report oxidative stress outcomes in either included study [41,42].

### 4.3. Isoflavones

Isoflavones are structurally distinct from other flavonoid subclasses by the attachment of the B-ring at the C3 rather than C2 position of the central heterocyclic ring, conferring a structural resemblance to endogenous estrogens and enabling interaction with estrogen receptors, tyrosine kinases, and redox-sensitive transcription factors [25]. This subclass was represented by 12 preclinical studies examining six compounds: genistein (*n* = 5), puerarin (*n* = 2), formononetin (*n* = 2), and biochanin A, daidzein, and a genistein/daidzein combination (each *n* = 1). STZ induction was the most common T1D model (*n* = 7), utilized across C57BL/6 mice (*n* = 4), Sprague Dawley rats (*n* = 2), and Wistar rats (*n* = 1), followed by alloxan induction in two studies using Kunming and C57BL/6J mice, respectively, and spontaneous NOD mouse models in three studies. Genistein, the most studied isoflavone in this review, is a naturally occurring phytoestrogen found in soy products that has been characterized for its capacity to inhibit tyrosine kinase signaling, modulate immune cell activation, and exert antioxidant effects in diabetic tissues [60].

**Glycemic Control:** Glycemic outcomes were reported in all 12 isoflavone studies, with significant improvement found in seven studies (58.33%), partial improvement in two studies (16.67%) and no improvement in three studies (25%). Puerarin demonstrated the most consistent and mechanistically detailed glycemic activity: in one study, it dose-dependently reduced fasting blood glucose from the second week of administration, significantly restored impaired glucose tolerance, and reduced fructosamine levels [61]; in a second study, puerarin significantly reduced blood glucose, improved glucose tolerance AUC, and increased serum insulin, with in vitro evidence linking these effects to PI3K/Akt-mediated β-cell survival [62]. Formononetin produced significant glycemic improvement in both studies: with alloxan models demonstrating significant reductions in fasting blood glucose and OGTT AUC and significantly increased serum insulin at doses ranging from 2.5–20 mg/kg/day. Further, effects were shown to be abolished in Nrf2-knockout mice, establishing a causal mechanistic link [63,64]. Biochanin A also significantly reduced blood glucose at both doses tested in STZ-induced rats [65].

Genistein produced significant glycemic improvement in three of five studies. In NOD mice, genistein significantly reduced blood glucose and delayed T1D onset, with significant improvements in fasting blood glucose and 120 min blood glucose in a glucose tolerance test (GTT) in males (but with no significant change in blood glucose levels in females) [66]; genistein-loaded chitosan nanoparticles administered via pulmonary delivery significantly reduced blood glucose levels [67]; and genistein significantly reduced blood glucose in STZ-induced mice, though without significant effect on plasma insulin [68]. The combined genistein and daidzein study in NOD mice demonstrated significant reductions in fasting blood glucose and intraperitoneal glucose tolerance test (IPGTT) glucose levels with significantly elevated plasma insulin at week 9, providing evidence for additive isoflavone effects on glucose regulation [69]. In contrast, daidzein administered as a standalone agent did not demonstrate significant T1D protection in either sex on blood glucose longitudinal curves, GTT, or insulin tolerance test (ITT) in NOD mice [70]. Two genistein studies reported no significant glycemic effect: the myocardial fibrosis study and the wound healing study, in which genistein at 0.2–5 mg/kg did not significantly modify blood glucose despite significant vascular superoxide attenuation [71,72].

**Oxidative Stress:** Oxidative stress outcomes were reported in 6 of the 12 isoflavone studies, a notably lower reporting rate than observed in the flavonol subclass. Among those reports, findings were universally positive (100%). Formononetin significantly decreased intracellular ROS and downregulated Keap1 while upregulating Nrf2, HO-1, and NQO1 protein expression in pancreatic tissue, with effects abolished in Nrf2-knockout animals, confirming pathway specificity [64]. Genistein significantly attenuated cutaneous superoxide production and dose-dependently suppressed nitrotyrosine accumulation in the wound healing study, significantly reduced urinary TBARS excretion and renal gp91phox expression in the nephropathy study and demonstrated significant reductions in erythrocyte and hepatic lipid peroxidation in the combined genistein and daidzein study [68,69,72].

Puerarin significantly reduced plasma MDA and elevated SOD activity and GSH levels in the hypoglycemic activity study and significantly decreased ROS generation in MIN6 β-cells alongside upregulation of MnSOD and GPx1 mRNA expression in the β-cell survival study [61,62]. Oxidative stress was not assessed in the remaining seven isoflavone studies, including all biochanin A, daidzein standalone, genistein/myocardial fibrosis, genistein/NOD, and genistein/nanoparticle studies, representing a meaningful gap in the oxidative characterization of this subclass.

### 4.4. Flavanols

Flavanols (flavan-3-ols) are characterized by a hydroxyl group at the C3 position and the absence of a C4 carbonyl and C2–C3 double bond, features that permit conformational flexibility and underlie their capacity to form oligomeric and polymeric proanthocyanidin structures [25]. This subclass was represented by 11 preclinical studies examining six compounds: epigallocatechin-3-gallate (EGCG, *n* = 5), epicatechin (*n* = 2), and (−)-epicatechin-3-O-β-D-allopyranoside (BB), cocoa flavanol, procyanidin B2, and pycnogenol (each *n* = 1). STZ induction was used in nine studies across Wistar rats (*n* = 3), Sprague Dawley rats (*n* = 2), C57BL/6 mice (*n* = 2), C57BL/6J mice (*n* = 1), and C57BL/KsJ mice (*n* = 1), with the remaining two studies utilizing spontaneous NOD/LtJ mouse models. EGCG, the most studied flavanol in this review, is the principal bioactive catechin of green tea and has been characterized for its capacity to suppress autoimmune β-cell destruction, modulate Th1/Th2 immune balance, and attenuate oxidative stress via iNOS suppression and mitochondrial protective mechanisms [73].

**Glycemic Control:** Glycemic outcomes were reported in all 11 flavanol studies, with positive findings noted in 10 studies (90.91%). EGCG produced significant glycemic improvement in all studies reporting this outcome, with the sole exception of the EGCG study evaluating cardiac injury in STZ-induced rats, where it observed no significant effect on blood glucose, pointing towards a primary vascular rather than glycemic target in that model [74]. In NOD mice, EGCG significantly ameliorated hyperglycemia, improved fasting blood glucose, glucose tolerance, and HbA1c, and increased plasma insulin levels over 17 weeks [75]. In STZ-induced C57BL/KsJ, C57BL/6 mice and Wistar rats, it was shown that EGCG alone significantly reduced hyperglycemia and fasting blood glucose and, when combined with bone-marrow-derived mesenchymal stem cells, significantly reduced blood glucose and increased fasting serum insulin [76,77,78]. Next, epicatechin and its derivatives found universal improvement in glycemic control in all three of its studies. Epicatechin administered prior to STZ induction in rats maintained blood glucose within the upper limit of the normal range, significantly lower than untreated diabetic controls, while dietary epicatechin in NOD mice was associated with significantly higher plasma insulin and significantly lower HbA1c relative to controls [79,80]. Lastly, the BB epicatechin glycoside significantly reduced blood glucose and HbA1c at all tested doses and increased serum insulin at the two higher doses in STZ-induced C57BL/6J mice [81].

Pycnogenol demonstrated robust glycemic improvement, significantly reducing fasting blood glucose and HbA1c and improving insulin levels, amylase activity, and hepatic glycogen content in STZ-induced rats [82]. Procyanidin B2 significantly reduced serum glucose levels in STZ-induced C57BL/6 mice [83]. Cocoa flavanol supplementation also significantly potentiated post-exercise glycemic reduction at 60 min and significantly reduced glucose AUC following aerobic exercise in diabetic rats, suggesting exercise-dependent rather than baseline glycemic effects [84].

**Oxidative Stress:** Oxidative stress outcomes were reported in 6 of the 11 flavanol studies, with all six studies demonstrating a significant improvement (100%). EGCG demonstrated the most mechanistically detailed antioxidant activity. In the cardiac injury study, EGCG significantly reduced cardiac MDA and 15-F2t-isoprostane formation and increased MnSOD protein expression, with effects abolished by the Sirt1 inhibitor EX527, implicating Sirt1-dependent antioxidant signaling [74]. In STZ autoimmune models, EGCG markedly suppressed iNOS mRNA expression in isolated islets, attenuating reactive-nitrogen-species-mediated β-cell damage, and green tea extract significantly restored MDA, PCO, GSH, GPx, GST, SOD and CAT levels [76,78].

Epicatechin significantly reduced nitrite production in STZ-treated islets in a dose-dependent manner in vitro, providing direct evidence for reactive nitrogen species attenuation at the level of the pancreatic islet [80]. Pycnogenol significantly reduced TBARS and protein carbonyl content and significantly increased GSH and GST activity in liver and pancreatic tissue [82]. Procyanidin B2 significantly reduced MDA in peripheral blood and attenuated superoxide production in dermal wound tissue [83]. Oxidative stress was not assessed in the BB, cocoa flavanol, dietary epicatechin, or two of the four EGCG studies, limiting the oxidative characterization across a substantial portion of this subclass.

### 4.5. Flavones

Flavones are structurally defined by a C2–C3 double bond and a C4 carbonyl in the central ring, with the B-ring attached at C2 and an absence of the C3 hydroxyl that characterizes flavonols, resulting in a planar structure that facilitates intercalation with protein binding domains [25]. This subclass was represented by 10 preclinical studies examining seven compounds: apigenin (*n* = 3), silymarin (*n* = 2), and baicalein, diosmin, luteolin, nobiletin, and silybin (each *n* = 1). STZ induction was used in eight studies across Wistar rats (*n* = 3), C57BL/6 mice (*n* = 2), wild-type and 12/15-LO-deficient C57BL/6J mice (*n* = 1), and Sprague Dawley rats (*n* = 1); the remaining studies utilized an alloxan-induced albino mouse model (*n* = 1) and a genetic OVE26 mouse model of T1D (*n* = 1). Luteolin, among the most extensively characterized flavones in the broader literature, is found in celery, thyme, and parsley and has been shown to simultaneously suppress NF-κB-mediated inflammation and activate Nrf2-dependent antioxidant defenses in diabetic cardiac tissue [85].

**Glycemic Control:** Glycemic outcomes were reported in all flavone studies, with significant improvement observed in only four (40%). Apigenin produced significant glycemic improvement in two of three studies: administered subcutaneously at 0.78 mg/kg/day, it significantly decreased fasting blood glucose in a genitourinary dysfunction model and significantly decreased fasting blood glucose in a cardiac complication model with greater effect observed with early versus late administration [86,87]. The third apigenin study, evaluating nephropathy at oral doses of 5, 10 and 20 mg/kg/day over 8 months, reported no significant effect on blood glucose at any dose [88]. Silymarin similarly had mixed results, with administration at 40 and 80 mg/kg/day significantly reducing fed blood glucose at days 14 and 21 in STZ-induced albino mice, while the lower 20 mg/kg dose did not achieve significance, suggesting a dose threshold effect which was contrasted by the second silymarin study where 50 and 100 mg/kg/day over 30 days in alloxan-induced Fischer rats reported no significant reduction in serum glucose at either dose [89,90].

Diosmin significantly decreased blood glucose in a dose-dependent manner at 80, 120, and 160 mg/kg in STZ-induced rats, with the higher doses achieving greater significance, and its glycemic mechanism was attributed to endogenous β-endorphin induction [91]. Luteolin, nobiletin, baicalein, and silybin each reported no significant effect on blood glucose in their respective models, indicating that glycemic control is not a consistent feature of this subclass [92,93,94,95].

**Oxidative Stress:** Oxidative stress outcomes were reported in 7 of the 10 flavone studies, with five studies demonstrating complete improvement (71.43%) and two studies reporting partial improvement (28.57%). Luteolin demonstrated significant antioxidant activity in the diabetic cardiomyopathy model, significantly reducing dihydroethidium (DHE)-positive staining and 3-nitrotyrosine content and restoring SOD activity, alongside significant upregulation of Nrf2 protein, Nrf2 mRNA, and HO-1 mRNA, all in the absence of glycemic improvement, reinforcing the Nrf2/NF-κB axis as the primary therapeutic mechanism for this compound [92]. Silymarin demonstrated significant antioxidant effects in both studies: at 40 and 80 mg/kg/day, it significantly reduced TBARS and protein carbonyl content and significantly increased total thiol molecules and total antioxidant capacity assessed by ferric reducing antioxidant power (FRAP) and reduced MPO activity in the murine STZ model while the second silymarin study found a more limited antioxidant profile, with significant reductions in hepatic and pancreatic protein carbonylation at both doses but no significant effects on SOD or CAT activity, suggesting that silymarin’s antioxidant benefit may be more selective toward carbonyl stress than enzymatic antioxidant restoration [89,90].

Apigenin at the highest dose (20 mg/kg/day) significantly reduced renal MDA and increased GSH, SOD, and CAT in a nephropathy model, while lower doses (5 and 10 mg/kg/day) did not achieve significance [88]. Nobiletin significantly reduced cardiac MDA and increased SOD1 activity and protein expression in the cardiomyopathy model [93]. Silybin significantly reduced renal superoxide production and prevented upregulation of Nox4 expression in the renal cortex of OVE26 diabetic mice, with effects independent of any glycemic change [95]. Baicalein, via pharmacological inhibition of 12/15-lipoxygenase, significantly reduced urinary TBARS excretion in the 10-week treatment group, though the 4-week group did not reach significance, again suggesting duration dependence for oxidative efficacy [94]. Oxidative stress outcomes were not reported in the two apigenin studies that assessed genitourinary and cardiac endpoints or the diosmin study [86,87,91].

### 4.6. Flavanones

Flavanones are characterized by a saturated C2–C3 bond and a C4 carbonyl in the central ring with no hydroxyl at C3, resulting in a non-planar, chiral structure that distinguishes this subclass from flavones and flavonols and influences receptor binding affinity and tissue distribution [25]. This subclass was the smallest represented in this review, comprising seven studies examining three compounds: naringin (*n* = 4), naringenin (*n* = 2), and hesperetin (*n* = 1). All seven studies utilized STZ-induced T1D models in Wistar rats (*n* = 6) and Sprague Dawley rats (*n* = 1). Naringin, the most studied flavanone in this review, is the predominant flavanone glycoside in grapefruit and citrus peel and has been characterized for its anti-inflammatory, antidyslipidemic, and cardioprotective properties in diabetic models [96].

**Glycemic Control:** Glycemic outcomes were reported in six of the seven flavanone studies, with consistent glycemic improvement observed only for hesperetin (16.67%) and inconsistent (50%) or absent (33.33%) effects for both naringin and naringenin. Hesperetin significantly decreased fasting blood glucose and 2 h postglucose-loading blood glucose, improved glucose tolerance, significantly reduced OGTT AUC, and significantly decreased serum fructosamine, representing the most comprehensive glycemic improvement profile in this subclass [97]. Naringenin did not significantly affect serum glucose in the only study reporting this outcome [98]. The other naringenin study exploring its effects on cardiac tissue did not report any glycemic control outcomes [99]. Among the four naringin studies, glycemic outcomes were decidedly mixed: naringin did not significantly reduce fasting blood glucose, glucose tolerance, or GTT AUC in two studies, but one study did show significant improvement in fasting plasma insulin [100,101], and in a third study naringin alone did not significantly affect blood glucose or serum insulin, with significant glucose reduction achieved only in the concurrent insulin plus naringin group [102]. The fourth naringin study reported a partial glycemic profile: naringin did not significantly improve fasting blood glucose or GTT AUC but did significantly reduce HbA1c, suggesting an effect on longer-term glycemic exposure without acute fasting glucose reduction [103]. Collectively, these findings indicate that flavanones as a subclass do not reliably reduce fasting blood glucose in STZ-induced T1D rodent models, with notable compound-specific exceptions.

**Oxidative Stress:** Oxidative stress outcomes were reported in five of the seven flavanone studies. Only one study demonstrated complete improvement in all oxidative stress markers (20%), with the remaining four studies demonstrating partial improvement in some of its markers (80%). Naringin significantly reduced plasma MDA concentrations in one study and significantly reduced both kidney tissue and erythrocyte TBARSs in another, though GSH was not significantly restored in either tissue in the latter study [100,103]. The last two naringin studies on dyslipidemia and combined treatment with insulin did not report oxidative stress markers [101,102]. Hesperetin significantly reduced hepatic MDA and lipid peroxidation, significantly increased hepatic GSH and GST, and increased glutathione reductase activity, though GPx was not significantly affected [97]. Naringenin produced a nuanced antioxidant profile: in the lens study, naringenin significantly reduced SOD and CAT activity alongside significant reductions in advanced oxidation protein products (AOPPs) and protein carbonyl content at the higher dose, with the authors interpreting SOD and CAT reductions as indicative of reduced oxidative load rather than antioxidant enzyme suppression; in the cardiac tissue study, naringenin did not significantly affect SOD, CAT, GPx, GSH, or total oxidant status but did significantly reduce AOPPs and MDA at both doses, suggesting selective attenuation of lipid peroxidation and protein oxidation without broad enzymatic antioxidant restoration [98,99].

## 5. Stilbenes

Stilbenes are a class of plant-derived polyphenolic compounds defined by a core 1,2-diphenylethylene (C6–C2–C6) skeleton, in which two phenyl rings are connected by an ethylene bridge. They are produced primarily as phytoalexins, stress-response molecules synthesized by plants in reaction to pathogenic attack, UV irradiation, or mechanical injury. The most extensively studied natural stilbene is resveratrol (trans-3,5,4′-trihydroxystilbene), which bears three hydroxyl groups and occurs predominantly in the biologically active trans configuration; dietary sources include the skin of red grapes, blueberries, peanuts, and mulberries [104]. Structurally related analogues in this class include pterostilbene (trans-3,5-dimethoxy-4′-hydroxystilbene), which differs from resveratrol by substitution of two of the three hydroxyl groups with methoxy moieties, and piceatannol (trans-3,3′,4,5′-tetrahydroxystilbene), which bears an additional hydroxyl group relative to resveratrol. These structural differences carry significant pharmacokinetic consequences: pterostilbene’s dimethoxy substitution confers greater lipophilicity and membrane permeability, resulting in an oral bioavailability of approximately 80% compared with approximately 20% for resveratrol, which undergoes rapid first-pass conjugation to glucuronide and sulfate metabolites [105].

Widespread interest in resveratrol in metabolic disease research stems from its pleiotropic signaling activity across several pathways of direct relevance to T1D pathophysiology. In the context of glycemic control, resveratrol has been demonstrated to improve glucose homeostasis through activation of the SIRT1/AMPK axis, which promotes GLUT4 expression and translocation, enhances glucose uptake in skeletal muscle, reduces hepatic glucose production, and supports pancreatic β-cell survival by inhibiting apoptosis via FOXO1 deacetylation [19,106]. With respect to oxidative stress, resveratrol activates the Nrf2/ARE transcriptional pathway, upregulating downstream cytoprotective genes including HO-1, NQO-1, SOD, and catalase, thereby amplifying endogenous antioxidant defenses in metabolically stressed tissue [107]. These same mechanisms also intersect with the AGE–RAGE–oxidative stress axis that drives diabetic end-organ injury, with resveratrol reported to suppress RAGE expression and attenuate downstream NF-κB-mediated ROS production and inflammatory signaling [19,108].

A total of 31 studies examined stilbene-class polyphenols in the context of T1D, making this the largest single-compound subgroup in the review. The predominant compound investigated was resveratrol (trans-resveratrol or racemic resveratrol), which accounted for 27 of 31 studies. The remaining studies examined structurally related stilbene analogues: pterostilbene (*n* = 2), piceatannol (*n* = 1), and an aza resveratrol–chalcone synthetic derivative (compound 6b; *n* = 1). Rodent species predominated, with Wistar rats (*n* = 10), Sprague Dawley rats (*n* = 5) and NOD mice (*n* = 3) most frequently used. T1D induction methods were mostly STZ (*n* = 26), followed by NOD (*n* = 3) and alloxan (*n* = 2).

**Glycemic Control:** Glycemic control outcomes were reported in 30 of 31 stilbene studies, with only a single trans-resveratrol study not recording glycemic outcomes [109]. Among those reporting glycemic endpoints, blood glucose was measured in all studies followed by insulin measured in 13 studies, with additional parameters like HbA1c, OGTT AUC and C-peptide being recorded in a handful of papers. Results were heterogeneous but leaned towards positive trends, with 17 papers recording significant improvement in all recorded metrics (56.67%), six papers recording partial improvements (20%) and seven papers recording no improvements at all (23.33%).

Studies that had significant improvement in all recorded metrics involved 14 papers testing resveratrol, single papers testing piceatannol and pterostilbene, and a paper investigating both resveratrol and pterostilbene. The resveratrol papers tested varying doses, ranging from 1 µg to 200 mg/kg/day; 10 of these papers utilized STZ-induced T1D models and found significantly decreased blood glucose in all treatments, as well as significantly improved insulin, C-peptide, HbA1C in select studies [110,111,112,113,114,115,116,117,118,119]. The remaining three resveratrol papers utilized a dose of 200 mg/kg/day and involved NOD mice, demonstrating significant reductions in blood glucose across all three studies and increased C-peptide across two [120,121,122]. Pterostilbene at 50 mg/kg/day significantly reduced blood glucose levels in alloxan-induced New Zealand rabbits [123]. Further, the combination study investigating both pterostilbene and resveratrol demonstrated that pterostilbene had a greater significant decrease in blood glucose, though both treatments reduced glucose and increased serum insulin [124]. The papers testing piceatannol revealed that 40 mg/kg/day was sufficient to significantly reduce blood glucose, restore insulin levels, and improve OGTT AUC levels [125].

Among the six studies reporting partial glycemic improvement, a consistent pattern emerged: resveratrol reliably reduced blood glucose at sufficient doses but failed to normalize all measured glycemic parameters, with HbA1c or insulin frequently remaining unaffected. Resveratrol at 25 mg/kg/day for 4 weeks in STZ-induced Sprague Dawley rats significantly decreased blood glucose and improved serum insulin, yet HbA1c was not significantly reduced [126]. A clear dose-dependent effect was evident: only resveratrol at the highest dose tested (10 mg/kg) significantly reduced fasting plasma glucose and improved fasting plasma insulin and HOMA-β and only trans-resveratrol at the highest dose tested (5 mg/kg/day) significantly reduced plasma glucose [127,128].

Two partial-improvement studies were notable for the context dependence of their glycemic findings. Resveratrol at 10 mg/kg/day IP for 30 days in insulin-treated STZ-induced Wistar rats showed that blood glucose did not significantly differ between the insulin-alone and insulin-plus-resveratrol groups; however, resveratrol co-administration significantly reduced urinary glucose excretion and restored plasma fructosamine to non-diabetic levels compared with insulin alone, indicating an improvement in overall glycemic burden not fully captured by fasting glucose [129]. Next, a time-dependent partial response in STZ-induced CD1 mice receiving resveratrol at 30 mg/kg/day for 12 weeks was recorded, with no significant glucose reduction at week 8 but a significant reduction at week 12, suggesting that certain glycemic endpoints may require extended treatment duration to manifest [130]. Contrary to this, trans-resveratrol only reduced blood glucose at week 1 with no sustained glycemic effect beyond this initial time point in a study involving STZ-induced Agouti rats [131]. Finally, the last trans-resveratrol study demonstrated significantly increased serum insulin without significantly reducing blood glucose, suggesting a possible enhancement of residual beta-cell secretory function that was insufficient to correct systemic hyperglycemia in this model [132].

Seven studies reported no significant glycemic improvement despite producing meaningful effects in other outcome domains. Four of these involved resveratrol in cardiac-focused models. Resveratrol administered at varying doses did not significantly improve blood glucose in any of these, despite largely improving cardiac parameters [133,134,135]. The last cardiac-focused paper reported that the aza resveratrol–chalcone derivative 6b at both 5 and 20 mg/kg/day orally for 16 weeks produced no significant effect on fasting blood glucose or serum insulin [136]. The remaining three studies that found no glycemic improvement showed that resveratrol and a resveratrol–hydroxypropyl-β-cyclodextrin complex had no improvement in STZ-induced Wistar and Sprague Dawley rats [137,138,139].

Taken together, the stilbene glycemic data indicate that significant glycemic improvement is achievable, particularly with resveratrol and its structural analogues at doses ≥ 5 mg/kg/day. However, this effect is neither consistent nor predictable across models, with outcome, dose, treatment duration, and the presence of background insulin therapy each acting as important moderating variables that preclude a unified conclusion regarding glycemic efficacy in preclinical T1D.

**Oxidative Stress:** Oxidative stress outcomes were reported in 24 of 31 stilbene studies, leaving seven studies that did not assess oxidative stress markers [118,128,129,133,137,138,139]. Among studies reporting oxidative stress endpoints, the markers assessed included lipid peroxidation products, most commonly MDA or thiobarbituric acid reactive substances (TBARSs) and antioxidant enzymes including SOD, catalase, and glutathione peroxidase. The oxidative stress data were substantially more consistent than glycemic outcomes: of the 24 studies reporting oxidative stress parameters, 20 reported significant improvement in all measured markers (83.33%) and four reported partial improvement (16.67%). No stilbene study reporting oxidative stress outcomes observed a complete absence of antioxidant effect, underscoring the robustness of the antioxidant signal in this compound class.

Resveratrol demonstrated the broadest and most consistent antioxidant activity across the greatest diversity of target organs in this subgroup spanning cardiac, renal, pancreatic, muscle and reproductive tissue.

In cardiac tissue, six studies documented full significant improvement in oxidative stress markers. Significant reductions in cardiac lipid hydroperoxides, protein carbonyls and advanced glycation end products alongside significant increases in GSH, GPx, and glutathione reductase were demonstrated with resveratrol [110,111]. Further extensions to SIRT3 demonstrated significant reductions in cardiac ROS and TBARSs alongside significant restoration of SOD, catalase, and GSH in concert with improved mitochondrial electron transport chain function [119]. Additionally, two cardiac studies were notable for demonstrating robust antioxidant activity in the complete absence of glycemic effect, reinforcing the dissociation between these outcome domains: resveratrol resulted in significant upregulation of cardiac Nrf2 protein and its downstream targets HO-1, NQO-1, SOD1, and SOD2, as well as significant reductions in 3-nitrotyrosine and 4-hydroxynonenal, and the aza resveratrol–chalcone derivative 6b significantly reduced DHE-positive staining and 3-nitrotyrosine while significantly upregulating Nrf2, HO-1, and NQO-1 in diabetic cardiac tissue at both doses tested, despite no glycemic benefit [134,136]. Lastly, cardiac antioxidant activity was operationalized through SIRT1 deacetylase activity, demonstrating that resveratrol stimulated cardiac SIRT1 activity to approximately 40% above control levels, a finding consistent with SIRT1’s established role as a redox-sensitive NAD^+^-dependent enzyme whose activity is suppressed under hyperglycemic conditions [135,140].

In renal tissue, three studies reported full significant oxidative stress improvement. Significant reductions in MDA and increases in Mn-SOD activity were observed in the renal cortex via the SIRT1/PGC-1α pathway [130]. In NOD mouse models, significant reductions in RAGE, NF-κB (p65), and either MCP-1 or NOX4 expression were demonstrated across two independent studies using the same model and dose, indicating convergent attenuation of the RAGE–oxidative stress signaling axis and suggesting that resveratrol’s renal antioxidant effects may be particularly relevant to the inflammatory–oxidative milieu of spontaneous autoimmune T1D nephropathy [120,121]. In pancreatic and islet tissue, three studies assessing inflammatory–oxidative markers reported full significant improvement. Significant reductions in serum MDA and increases in blood GSH were documented in two studies using identical STZ-induced BALB/c mouse models at 50 mg/kg/day for 12 days, with significant reductions in serum nitric oxide reported under the same treatment conditions in a third study, collectively demonstrating consistent suppression of reactive nitrogen species and lipid peroxidation products in this model [115,116,117]. In skeletal muscle, significant reductions in superoxide anion content were observed in both EDL and soleus muscle at doses as low as 1 µg/kg/day, with fiber-type-specific differential regulation of CuZnSOD and MnSOD protein expression, representing the lowest effective antioxidant dose in this subgroup [113]. Both resveratrol and pterostilbene at 20 mg/kg/day significantly reduced TNF-α and NF-κB levels in soleus and EDL muscles, with resveratrol significantly more effective than pterostilbene in attenuating these markers in both muscle types [124]. In reproductive tissue, consistent MDA reductions were observed across three studies. Significant reductions in testicular MDA and nitrite were reported at 150 mg/kg/day, with the latter study additionally demonstrating MDA reduction across the caput, corpus, and cauda epididymis [112,114]. Lastly, a significant reduction of NF-κB expression in sciatic nerve tissue in NOD mice treated with resveratrol provided the only neuropathy-specific antioxidant data point in this subgroup [122].

Pterostilbene produced the most comprehensive antioxidant profile of the non-resveratrol stilbenes. It was shown to significantly restore retinal CAT, GPx, and SOD activity, improve the GSH/GSSG ratio, and reduce protein carbonylation, 4-HNE, and H_2_O_2_ levels, with mechanistic data further showing significant restoration of PI3K/AKT/GSK3β phosphorylation and NQO-1 expression, implicating the Nrf2 pathway as a mediator of pterostilbene’s retinal antioxidant protection [123]. Next, piceatannol was shown to significantly reduce hepatic MDA while significantly restoring SOD and GSH-Px activity in STZ-induced C57BL/6 mice [125]. Finally, trans-resveratrol treatment significantly reduced lipid hydroperoxide, suppressed basal and NAD(P)H-stimulated superoxide production, and increased aortic and serum nitrite/nitrate levels [132].

Four studies reported partial oxidative stress improvement. Resveratrol demonstrated significant reductions in TBARSs and significant increases in SOD and GSH in both pancreatic and hepatic tissue, but hepatic GSH was not significantly restored, precluding full improvement classification [126]. Another resveratrol study found a dose-dependent antioxidant response mirroring the glycemic findings: only the 10 mg/kg dose significantly reduced MDA and significantly increased total antioxidant capacity, while the 0.1 and 1 mg/kg groups showed no significant effect on either marker [127]. Two studies demonstrated the partial oxidative stress improvement in trans-resveratrol, both lacking conventional antioxidant enzyme panels that precluded full improvement classification [109,131].

Taken together, the stilbene data consistently support a tissue-protective antioxidant effect that is dissociable from glycemic improvement in a substantial subset of studies, with mechanistic data converging on Nrf2/ARE activation, sirtuin-mediated redox signaling, and suppression of NADPH-oxidase-derived ROS as primary effector pathways [106,107,108,140].

## 6. Diarylheptanoids

Diarylheptanoids are a class of phenolic compounds defined by two aryl groups connected by a seven-carbon aliphatic chain, with curcumin (1,7-bis(4-hydroxy-3-methoxyphenyl)-1,6-heptadiene-3,5-dione) representing the principal and best-characterized member [141]. The linear curcuminoid scaffold features a central β-diketone moiety capable of keto-enol tautomerism, flanked by two feruloyl-derived phenolic rings each bearing a hydroxyl and methoxy substituent, structural features that collectively underlie curcumin’s direct radical-scavenging capacity and its ability to interact with redox-sensitive transcription factors and protein kinases [142]. This class was represented by 24 studies across two subfamilies: curcumin (*n* = 19) and curcumin derivatives (*n* = 6), with one study (SLR-18/SLR-D5) contributing to both subfamilies by directly comparing curcumin to a water-soluble derivative.

### 6.1. Curcumin

This subfamily comprised 19 studies, all conducted in STZ-induced (*n* = 18) or alloxan-induced (*n* = 1) T1D models. STZ models utilized Sprague Dawley rats (*n* = 10), Wistar rats (*n* = 5), C57BL/6 mice (*n* = 3), and BALB/c mice (*n* = 1). Curcumin, the most studied diarylheptanoid in this review, is the primary bioactive curcuminoid of *Curcuma longa* (turmeric) and has been extensively characterized for its ability to activate the Nrf2/Keap1/ARE pathway, upregulate downstream cytoprotective enzymes including HO-1 and NQO-1, and suppress NF-κB-mediated inflammatory signaling in the context of diabetic organ injury [143].

**Glycemic Control:** Glycemic outcomes were reported in all 19 curcumin studies. Significant improvement in all assessed glycemic metrics was observed in 10 studies (52.63%), partial improvement in six studies (31.58%), and no significant improvement in three studies (15.79%). Curcumin most consistently reduced blood glucose at doses of 100 mg/kg/day. Across eight studies employing this dose in STZ-induced Sprague Dawley or Wistar rats over 6–12 weeks, six reported significant reductions in blood glucose, with several additionally documenting significant improvements in plasma insulin, HbA1c, C-peptide, and glycated hemoglobin [144,145,146,147,148]. The seventh study at 100 mg/kg/day, targeting diabetic nephropathy over 12 weeks, observed no significant reduction in plasma glucose or restoration of plasma insulin, despite robust renal antioxidant activity, representing a clear dissociation between glycemic and oxidative stress outcomes at this dose [149]. Next, the study that tested per os (p.o.; oral administration) administration of 100 mg/kg/day also showed no significant improvement in blood glucose, despite the 7.5 mg/kg/day intraperitoneal (i.p.) treatment and 7.5 mg/kg treatment of cur microparticles both significantly reducing blood glucose [150]. The last study that utilized curcumin at 100 mg/kg/day did not significantly decrease blood glucose but did demonstrate that 200 mg/kg/day did significantly decrease glucose in a dose-dependent response [151]. This dose-dependent glycemic effect was supported by an additional study that demonstrated that, at 50 and 150 mg/kg/day, only the 150 mg/kg/day dose significantly reduced fasting blood glucose in Wistar rats, while 50 mg/kg/day did not reach significance [152]. Interestingly, another study at 150 mg/kg/day did not significantly reduce blood glucose in a gastric motility model despite significant gastric antioxidant improvement [153].

Other doses that lead to significant improvement in glycemic control included the high dose of 1.5 g/kg/day [154], 30 mg/kg/day [155], and both 80 and 130 mg/kg/day [156]. However, another study at the same high dose of 1.5 g/kg/day demonstrated no significant improvement in glucose or insulin, despite significant attenuation of skeletal muscle oxidative stress, again illustrating compound behavior that is independent of glycemic correction at even very high doses [157].

Formulation studies provided evidence that delivery method substantially modified glycemic efficacy. The study discussed above utilizing intraperitoneal and sustained-release curcumin microparticle (CuMP) formulations produced significant blood glucose reductions, while oral conventional curcumin at 100 mg/kg/day did not achieve a significant glucose reduction in that study [150]. Similarly, chitosan-encapsulated curcumin (CEC) at 150 mg/kg/day significantly decreased fasting blood glucose and serum insulin in STZ-induced C57BL/6 mice, whereas conventional curcumin at the same dose did not reach significance for either outcome [158]. Nanoformulated curcumin (nCUR) administered as a single acute dose 6 h prior to STZ induction significantly reduced blood glucose at both 10 and 50 mg/kg in Sprague Dawley rats, and nCUR 50 mg/kg also significantly increased plasma insulin, while plain curcumin at 50 mg/kg did not significantly reduce blood glucose under the same preventive protocol [159]. The NCD + curcumin comparison demonstrated that curcumin alone significantly decreased plasma glucose, increased plasma insulin, and reduced glycated hemoglobin versus the diabetic group, though without normalization to control values [160]. Next, 1.0% curcumin by weight ratio mixed in the diet was able to successfully significantly reduce blood glucose and glucagon and increase insulin [161]. Lastly, a dose of 200 mg/kg/day in a myocardial infarction co-morbidity model significantly lowered blood glucose, but with curcumin nanoparticles additionally producing significantly higher C-peptide levels compared to conventional curcumin at the same dose, suggesting formulation-dependent enhancement of residual β-cell function [162].

Collectively, these findings indicate that curcumin’s glycemic efficacy is neither dose-nor subclass-uniform but is substantially modulated by route of administration, formulation type, and target organ context. The consistent failure of oral conventional curcumin to achieve glycemic significance in studies where parenteral or nanoformulated delivery succeeded underscores poor bioavailability as the primary limiting factor for systemic glycemic activity in this subclass.

**Oxidative Stress:** Oxidative stress outcomes were reported in 15 of the 19 curcumin studies; of these, 12 demonstrated significant improvement in all assessed markers (80%), three showed partial improvement (20%), and four studies did not report oxidative stress outcomes [151,155,158,162]. The antioxidant profile of curcumin was broad, with effects demonstrated across hepatic, renal, cardiac, neurological, gastric, and skeletal muscle compartments.

The most mechanistically detailed hepatic antioxidant profile was documented in two liver-focused studies. In the Nrf2-focused liver study, curcumin significantly reduced plasma MDA and increased SOD, while the paradoxical elevated GSH-Px and CAT activity observed in untreated diabetic rats was significantly reduced by curcumin, an effect interpreted as normalization of a compensatory antioxidant overactivation. At the gene expression level, curcumin significantly upregulated hepatic CAT, GSH-Px, HO-1, and NQO-1 mRNA, increased nuclear Nrf2 accumulation, and significantly upregulated Keap1 protein expression versus diabetic controls, consistent with activation of the classical Nrf2/Keap1 cytoprotective axis [161]. In the second liver study, curcumin significantly reduced MDA and significantly increased SOD, liver and plasma GPx, and liver and plasma GSH in STZ-induced rats [154].

Renal antioxidant effects were documented in three studies. Curcumin significantly reduced renal ROS and MDA and significantly increased renal GSH, MnSOD protein and mRNA, and nuclear Nrf2 in a nephropathy model, establishing Nrf2 nuclear translocation as a key mechanistic feature of renal protection, all in the absence of any glycemic correction [149]. In a second nephropathy study at 150 mg/kg/day, curcumin significantly restored TAC and TOS, reduced MDA, and increased SOD, GPx, and CAT activity in kidney tissue, with these effects dose-dependent as the lower 50 mg/kg/day dose did not produce significant glycemic improvement [152]. A third renal study at 130 mg/kg/day demonstrated significant increases in TAC and TTG and significant decreases in MDA, TOS, and NO, while 80 mg/kg/day did not significantly improve any of these oxidative markers despite producing significant fasting blood glucose reduction, indicating a higher dose threshold for renal antioxidant efficacy than for glycemic effect in this model [156].

Cardiac antioxidant effects were reported in two studies. In the PKC-MAPK cardiomyopathy study, curcumin significantly reduced left ventricular MDA and significantly increased GPx activity [147]. In the CaMKII/NF-κB cardiomyopathy study, curcumin significantly decreased myocardial MDA and significantly increased myocardial GSH and TAC [144]. In the NCD + curcumin comparison study, curcumin at 20 mg/kg/day significantly increased HO-1 gene expression and HO activity in cardiac and pancreatic tissue, though this upregulation was significantly lower than that achieved by NCD at the same dose [160].

Neurological, gastric and skeletal muscle antioxidant effects were each documented in single studies. In the hippocampal study, curcumin significantly reduced MDA and protein carbonyl and significantly increased TAC in brain tissue [145]. Curcumin at 150 mg/kg/day in the gastric motility model significantly reduced gastric MDA and significantly increased gastric SOD in the absence of glycemic correction [153]. In the skeletal muscle atrophy study, curcumin at 1500 mg/kg/day significantly decreased superoxide production and significantly reduced TBARSs in muscle tissue of diabetic mice, despite producing no glycemic effect, again illustrating dissociation between antioxidant and glycemic activity [157].

Partial antioxidant improvement was observed in three studies. In the monocyte/inflammatory cytokine study, curcumin significantly reduced plasma protein carbonyl but did not significantly reduce erythrocyte lipid peroxidation [148]. The nephropathy study assessing KIM-1 and NGAL at 80 mg/kg did not significantly improve TAC, TTG, MDA, or TOS, and NO remained significantly elevated relative to control at this dose, with significance only achieved at 130 mg/kg [156]. In the curcumin microparticle formulation study, IP curcumin and CuMPs both significantly decreased MDA and NO, and CuMPs significantly maintained GSH tissue stores, while the oral curcumin arm reduced MDA at lower significance only, indicating formulation-dependent antioxidant efficacy paralleling the glycemic findings [150].

Across all 15 studies reporting oxidative stress outcomes, curcumin’s antioxidant activity was substantially more consistent and organ-spanning than its glycemic effects, with Nrf2 nuclear translocation and downstream HO-1 and NQO-1 upregulation emerging as the dominant mechanistic signature across hepatic, renal, and cardiac tissues. Notably, the repeated dissociation between antioxidant efficacy and glycemic correction suggests that curcumin’s primary therapeutic mechanism in STZ-induced T1D operates through redox-regulatory pathways independently of insulin secretion or glucose homeostasis.

### 6.2. Curcumin Derivatives

This subfamily comprised six studies examining four structurally distinct curcumin analogues: the novel curcumin derivative (NCD, a water-soluble analogue; *n* = 3, including the comparative study), J147 (*n* = 1), JM-2 (*n* = 1), and the monocarbonyl analogues C66 and B2BrBC (*n* = 1). All six studies utilized STZ-induced T1D models across Curl:HEL1 rats (*n* = 2), Wistar rats (*n* = 2), Swiss Webster mice (*n* = 1), and C57BL/6 mice (*n* = 1). Curcumin derivatives in this review represent compounds in which the parent diarylheptanoid scaffold has been structurally modified, through enhanced water solubility, monocarbonyl substitution, or synthetic analogue design, primarily to overcome curcumin’s pharmacokinetic limitations of poor bioavailability and aqueous instability [163].

**Glycemic Control:** Glycemic outcomes were reported in all six derivative studies, with significant improvement in all assessed metrics in four studies (66.67%), partial improvement in one study (16.67%), and no significant improvement in one study (16.67%). NCD demonstrated the most consistent and mechanistically progressive glycemic activity across its three studies. In the short-term study, NCD administered at 10 mg/kg/day for 45 days to STZ-induced Curl:HEL1 rats significantly lowered blood glucose and significantly increased plasma insulin [164]. In the long-term study by the same group, 150 mg/kg/day over 40 days and extended to 2 months from diabetes onset produced significant progressive reductions in fasting plasma glucose at both time points, alongside significant and sustained increases in plasma insulin and C-peptide levels, suggesting preservation and possible restoration of functional β-cell mass over time [165]. The NCD + curcumin comparison study directly compared NCD to curcumin (20 mg/kg/day, 45 days, Wistar rats). Curcumin alone significantly decreased plasma glucose, increased plasma insulin, and reduced glycated hemoglobin versus the diabetic group, though without normalization to control. NCD produced comparable glycemic improvement; both groups did not fully normalize to control levels, with the primary distinction between the two agents emerging in the oxidative stress domain [160].

J147, a synthetic curcumin-derived compound developed for neurological applications, significantly decreased blood glucose and HbA1c in STZ-induced Swiss Webster mice over 20 weeks but did not significantly alter plasma insulin, suggesting a mechanism independent of insulin secretion [166]. The monocarbonyl analogues C66 and B2BrBC produced compound-specific and administration-protocol-dependent glycemic results. Acute pretreatment with C66 (125 µmol/kg, single dose prior to STZ induction) significantly increased Ins1 mRNA and protein expression versus the STZ group, providing evidence for a preventive cytoprotective effect on β-cell gene expression; however, chronic treatment with either C66 or B2BrBC (50 µmol/kg/day over 30 days) did not reverse STZ-induced decreases in Ins1 or Glut2 expression, indicating that the β-cell-protective window for this analogue is narrow and limited to the preinjury period [167]. JM-2, a monoketone curcumin analogue administered at 10 mg/kg/day for 8 weeks to STZ-induced C57BL/6 mice in a cardiac injury model, did not significantly change fasting blood glucose or body weight [168].

Collectively, the glycemic profile of curcumin derivatives was comparable to but less than that of the parent compound (66.67% vs. 80% full improvement), though this comparison is tempered by the smaller derivative dataset and the heterogeneity of structural scaffolds represented. Notably, where curcumin’s glycemic limitations were primarily attributable to poor bioavailability and formulation-dependent absorption, the derivatives suggest an additional dimension of compound-specific mechanistic variation, with NCD demonstrating progressive β-cell preservation, J147 achieving glycemic reduction independent of insulin secretion, and C66 revealing a narrow preinjury cytoprotective window that was entirely lost under chronic administration.

**Oxidative Stress:** Oxidative stress outcomes were reported in four of the six derivative studies, with significant improvement in all markers in three (75%), partial improvement in one (25%), and two studies not reporting this outcome [165,166]. NCD demonstrated the most potent antioxidant activity within this subfamily, and its direct comparison to curcumin provided the most interpretively important oxidative stress finding in the diarylheptanoid class. Both curcumin and NCD significantly upregulated HO-1 gene expression and HO activity in cardiac and pancreatic tissue versus diabetic controls; however, NCD-induced HO-1 upregulation was significantly greater than that of curcumin at the same dose (20 mg/kg/day), identifying enhanced HO-1/Nrf2-axis engagement as the key mechanistic advantage of the water-soluble derivative over the parent compound [160]. In the short-term NCD study, NCD significantly lowered MDA and significantly increased HO-1 expression in pancreatic, hepatic, and aortic tissues [164]. Lastly, JM-2 was shown to significantly decrease superoxide production and TBARSs when delivered at a dose of 10 mg/kg/day over 8 weeks [168].

The monocarbonyl analogues C66 and B2BrBC demonstrated compound-specific and multi-organ antioxidant profiles across both experimental protocols. Under chronic treatment, B2BrBC significantly reduced MDA in plasma, liver, and kidney, reduced plasma AOPPs, and significantly increased SOD in plasma, CAT in liver and kidney, and GPx in kidney. C66 significantly reduced MDA and AOPPs in liver and plasma and significantly increased SOD in plasma and GPx in liver and kidney. Under acute pretreatment, B2BrBC significantly reduced plasma MDA, liver and kidney AOPPs, and normalized renal GPx, while acute C66 did not significantly alter any oxidative stress marker, indicating that C66’s antioxidant benefit, unlike its acute effect on β-cell gene expression, requires chronic administration [167]. Oxidative stress was not assessed in the J147 or NCD long-term study, representing gaps in the antioxidant characterization of this derivative subfamily.

Despite the limited number of derivative studies reporting oxidative stress outcomes, the findings collectively suggest that structural modification of curcumin can meaningfully amplify Nrf2/HO-1 axis engagement beyond what the parent compound achieves, as most directly evidenced by NCD’s superior HO-1 upregulation at an equivalent dose. In contrast to the parent curcumin subclass, where antioxidant efficacy was broad, consistent, and organ-spanning across 15 reporting studies, the derivative subclass remains substantially under-characterized in this domain, and the oxidative stress gap across J147, JM-2, and the NCD long-term study limits any definitive comparative conclusions at this stage.

## 7. Natural Polyphenol-Rich Extracts

The preceding sections examined the pharmacological activity of individual purified polyphenol compounds across the major flavonoid subclasses. The studies reviewed in this section take a complementary approach, investigating whole-plant or partially fractionated extracts in which the polyphenol content is characterized but not reduced to a single isolate. This distinction is pharmacologically meaningful: while isolated compounds permit mechanistic attribution, whole-plant extracts encompass a broader constellation of co-occurring polyphenols, phenolic acids, terpenes, and other secondary metabolites that may interact additively or synergistically to produce biological effects exceeding those of any individual constituent [169]. Synergistic interactions between polyphenols within a complex extract have been documented to amplify antioxidant, anti-inflammatory, and insulin-sensitizing effects beyond what would be predicted from the sum of individual compound activities, and in some instances the matrix of co-extracted compounds modulates bioavailability of active constituents in ways that isolated preparations cannot replicate [170,171]. In the context of T1D, where oxidative stress, autoimmune β-cell destruction, and chronic glycemic dysregulation converge across multiple pathological axes, the multi-target pharmacology inherent to polyphenol-rich extracts may represent a meaningful therapeutic advantage. A total of 59 preclinical studies were identified in this category, organized into four subsections based on the predominant polyphenol class or botanical source: flavonoid-rich plant extracts (*n* = 20), fruit and berry polyphenol extracts (*n* = 12), phenolic-acid- and tannin-rich extracts (*n* = 12), and other plant extracts (*n* = 15). All studies were conducted in rodent models of T1D with the exception of one study in alloxan-induced diabetic rabbits.

### 7.1. Flavonoid-Rich Plant Extracts

Flavonoid-rich plant extracts represent the largest natural extract subclass in this review, comprising 20 preclinical studies evaluating 18 distinct botanical preparations. The category includes extracts explicitly standardized to their flavonoid content, including total flavonoid fractions, flavonoid-rich fractions, and anthocyanin- and flavonolignan-rich preparations, as well as whole-plant extracts in which flavonoids constitute the characterized dominant polyphenol class. Extracts included: *Hibiscus sabdariffa* polyphenol-rich extract (HPE; *n* = 2), fermented soy permeate (FSP; *n* = 2), *Tephrosia purpurea* alcoholic extract and flavonoid-rich fraction (AcTp/FFTp), total flavonoids of *Polygonatum odoratum* (TFP), total flavonoids of *Sedum aizoon* L. (STF), flavonoid-rich fraction of *Lithocarpus polystachyus* Rehd. leaves (ST-3), *Ocimum gratissimum* flavonoid-rich extract (OGFL), propolis ethanolic extract (EEP), milk thistle seed extract (MTSE), *Cassia glauca* polyphenolic extract (CGE), *Simarouba glauca* leaf extract, *Momordica charantia* fruit juice extract, *Sphaeranthus indicus* root ethanolic extract (EESIR), *Hybanthus enneaspermus* alcoholic extract (AHE), *Psychotria dalzellii* methanol extract (MEPD), *Crataegus flavonoids* (CF), Synacinn™, and a polyherbal mixture (all *n* = 1).

STZ induction was the predominant T1D model, used in 17 studies across Wistar rats (*n* = 9), Sprague Dawley rats (*n* = 6), and C57BL/6 and NIH Swiss Outbred mice (each *n* = 1). The two *H. sabdariffa* extracts are derived from roselle calyces and have been characterized for their dense anthocyanin profile, with proposed mechanisms encompassing free radical scavenging, NF-κB suppression, and attenuation of hyperglycemia-induced endothelial oxidative injury [172].

**Glycemic Control:** Glycemic outcomes were reported in 19 of the 20 studies; of these, 15 (78.95%) demonstrated significant improvement in all assessed glycemic metrics, three (15.79%) demonstrated partial improvement, and one (5.3%) showed no significant effect. The two *H. sabdariffa* HPE studies both produced significant glycemic reductions: in STZ-induced Sprague Dawley rats at 100 mg/kg/day over 8 weeks, HPE significantly decreased plasma glucose, and at the same dose over 4 weeks in a second STZ-Sprague-Dawley model, HPE similarly significantly reduced plasma glucose [173,174]. The total flavonoids of *Sedum aizoon* L. (STF) at 200 mg/kg/day over 7 weeks in STZ-induced C57BL/6 mice significantly reduced fasting blood glucose and significantly improved OGTT glucose levels and AUC [175]. *S. indicus* root extract (EESIR) at 100 and 200 mg/kg/day over 28 days in STZ-induced Wistar rats produced dose-dependent reductions in blood glucose, with the 200 mg/kg dose significantly more effective than the 100 mg/kg dose [176]. *Cassia glauca* polyphenolic extract (CGE) at 400 mg/kg/day over 15 days significantly reduced serum glucose, improved OGTT AUC glucose, increased AUC insulin, and reduced ITT AUC glucose in STZ-induced Wistar rats [177]. *Simarouba glauca* leaf extract at 250 and 500 mg/kg/day over 30 days significantly decreased blood glucose and HbA1c in STZ-induced Wistar rats [178]. AcTp (300 mg/kg/day) and FFTp (40 mg/kg/day) both significantly decreased serum glucose and significantly increased serum insulin in STZ-induced Sprague Dawley rats over 21 days [179]. Propolis ethanolic extract (EEP) at both 100 and 200 mg/kg/day significantly reduced serum glucose in STZ-induced Wistar rats over 6 weeks [180]. MTSE/ZnO/Ag nanocomposite significantly decreased fasting blood glucose and significantly increased plasma insulin in alloxan-induced Wistar rats over 16 days, whereas the parent MTSE compound achieved the same outcome [181]. *M. charantia* fruit juice extract at 10 mL/kg/day over 28 days significantly decreased blood glucose in STZ-induced Wistar rats [182]. AHE (*H. enneaspermus*) at 250 and 500 mg/kg/day significantly reduced fasting plasma glucose on days 7, 14, and 21 in STZ-induced Wistar rats [183]. MEPD (*P. dalzellii*) at 200 mg/kg/day significantly decreased blood glucose in alloxan-induced Wistar rats over 20 days [184]. Synacinn™ at 250 mg/kg twice daily was the only dose achieving significant fasting blood glucose improvement across its five-dose escalation study in STZ-induced Sprague Dawley rats over 28 days [185]. The ST-3 (*L. polystachyus*) study reported significant reductions in fasting serum glucose and significant increases in fasting serum insulin in STZ-induced Sprague Dawley rats [186]. Lastly, the polyherbal mixture at 10 and 20 g/kg/day in alloxan-induced Wistar rats significantly reduced blood glucose, with the 20 g dose reaching significant reduction by day 7 while the 10 g dose took until day 14. Notably, both mixtures were shown to be significantly more effective than either insulin or metformin [187].

Partial glycemic improvement was observed in three studies. First, fermented soy peptide (FSP) was the only other compound studied twice but had contrasting results between studies: delivered at 1 mg/kg/day it was shown to be unable to significantly lower blood glucose but was able to restore muscle glucose content, while, being delivered at 0.1 g/day, it was unable to significantly affect blood glucose or plasma fructosamine, both in STZ-induced rats [188,189]. TFP (*P. odoratum*) at 100 and 200 mg/kg significantly reduced fasting blood glucose in STZ-induced mice by day 6, and all three doses (50, 100, 200 mg/kg) achieved significance by day 9, but insulin levels were not significantly affected at any dose [190]. The *Crataegus* flavonoids (CF) arm of the *Astragalus polysaccharides* (APS) + CF combination study demonstrated that CF alone did not significantly reduce fasting blood glucose after 2 weeks; significant reductions in fasting blood glucose, serum insulin, and OGTT AUC were achieved only in the combined APS + CF treatment group [191]. Finally, glycemic outcomes were not reported in the OGFL (*O. gratissimum* leaf extract) study, which was designed with an oxidative stress focus, discussed next [192].

Collectively, flavonoid-rich plant extracts demonstrated a high rate of glycemic efficacy in this review, with the majority of studies achieving significant improvement across all assessed glycemic metrics. However, the heterogeneity of botanical sources, extract preparations, doses, and animal models across this subclass precludes direct inter-study comparison, and the dependence of glycemic effect on combination therapy underscores that extract-level glycemic activity is not uniformly intrinsic to flavonoid content but is shaped by preparation-specific and context-specific variables.

**Oxidative Stress:** Oxidative stress outcomes were reported in 11 of the 20 studies; of these, eight (72.7%) demonstrated significant improvement across all assessed markers and three (27.3%) demonstrated partial improvement, with no study reporting a complete absence of antioxidant effect. STF demonstrated the most mechanistically characterized antioxidant profile: at the SM (100 mg/kg) and SH (200 mg/kg) doses, STF significantly increased T-SOD, GSH, and CAT activity and significantly reduced MDA content in STZ-induced C57BL/6 mice, with concurrent significant upregulation of Nrf2 nuclear expression, HO-1, and NQO1, and significant reduction in Keap1 expression, establishing activation of the Nrf2/Keap1/ARE axis as the operative antioxidant mechanism [175]. CGE at 200 and 400 mg/kg/day significantly increased GSH and total thiols and significantly reduced TBARSs, with 400 mg/kg additionally producing significant increases in catalase activity [177]. EESIR at both doses significantly increased SOD, CAT, and GPx and significantly reduced TBARSs in STZ-induced Wistar rats, with a dose-dependent advantage at 200 mg/kg [176]. EEP significantly reduced renal MDA and increased renal SOD, GPx, and FRAP in STZ-induced Wistar rats [180]. *H. sabdariffa* HPE significantly reduced cardiac MDA and AOPPs and significantly increased SOD-1, SOD-2, CAT, and GSH in STZ-induced Sprague Dawley rats, while the more recent HPE study similarly increased CAT activity and significantly reduced MDA and AOPPs in renal tissue but did not significantly restore SOD activity or GSH levels [173,174]. MEPD significantly reduced hepatic MDA/TBARSs and significantly increased hepatic GSH in alloxan-induced Wistar rats [184]. OGFL extract at 150 and 300 mg/kg/day significantly attenuated STZ-induced increases in brain MDA and significantly restored brain GSH, GST, CAT, GPx, and SOD [192]. Finally, *Simarouba glauca* leaf extract significantly reduced catalase activity and nitric oxide levels at both doses of 250 mg/kg and 500 mg/kg [178].

Partial antioxidant improvement was observed in three studies. *H. sabdariffa* HPE (2025) was addressed above compared to the other HPE study that showed significant improvement in all oxidative stress markers [173,174]. FSP significantly normalized SOD and GPx activity and significantly reduced plasma CML levels but did not significantly affect isoprostanes or the GSH/GSSG ratio [188]. The second FSP paper focused on skeletal muscle glucose levels and did not report any measures of oxidative stress [189]. AcTp and FFTp significantly prevented lens SOD depletion, GSH depletion, and lipid peroxidation in the diabetic cataract model, constituting full lens-specific antioxidant restoration, though the overall classification reflects the tissue-restricted scope of this assessment [179]. In addition to FSP, oxidative stress was not assessed in the AHE, TFP, MTSE, *M. charantia*, CF/APS, polyherbal, Synacinn™, or ST-3 studies, representing a substantial gap in oxidative stress characterization across this subclass [181,182,183,185,186,187,190,191].

Across this subclass, antioxidant efficacy was more consistent than glycemic efficacy, with all studies reporting oxidative stress outcomes demonstrating at least partial improvement, a pattern concordant with findings from the purified flavonoid subclasses reviewed earlier. Notably, the high proportion of studies not reporting oxidative stress outcomes (45%) represents a substantial evidence gap, and the tissue-specific nature of antioxidant assessments across studies, spanning renal, hepatic, cardiac, cerebral, and lens tissue, limits any unified interpretation of subclass-level antioxidant potency.

### 7.2. Fruit and Berry Polyphenol Extracts

Fruit and berry polyphenol extracts comprised 12 preclinical studies evaluating extracts derived from 10 distinct fruit and berry sources: grape extracts (grape seed extract (GSE), grape seed proanthocyanidin extract (GSPE), and freeze-dried grape powder) were the only source studied more than once (*n* = 3), followed by *Phyllanthus emblica* L. (Indian gooseberry), *Vaccinium* spp. bilberry/blueberry leaf and fruit extracts, cornelian cherry, apple polyphenols, passion fruit peel extracts (PFAE/PFHE), Corinthian currants, cranberry extract, Fu-Pen-Zi Chinese raspberry extract (FPZ), and *Emblica officinalis* fruit juice (all *n* = 1).

STZ induction was used in 10 studies across Wistar rats (*n* = 7), Sprague Dawley rats (*n* = 2), C57BL/6 mice (*n* = 1), and C57BL/6N mice (*n* = 1); NOD autoimmune models were used in two studies utilizing NOD mice (*n* = 1) and NOD/ShiLtJNarl mice (*n* = 1). Grape-derived extracts, representing the most studied fruit source in this subclass, are characterized by a dense proanthocyanidin content alongside anthocyanins and phenolic acids, have a documented capacity to reduce oxidative stress, attenuate pancreatic β-cell apoptosis, and modulate inflammatory cytokine expression in diabetic models [193].

**Glycemic Control:** Glycemic outcomes were reported in all 12 studies; six (50%) demonstrated significant improvement in all assessed metrics, three (25%) demonstrated partial improvement, and three (25%) showed no significant glycemic effect. Among grape-derived extracts, GSE at 300 mg/kg over 30 days in STZ-induced Sprague Dawley rats produced significant decreases in fasting blood glucose and serum fructosamine and significantly increased serum insulin when co-administered with mesenchymal stem cells (MSCs); GSE alone demonstrated only partial improvement in these parameters [194]. GSPE at a lower dose of 75 mg/kg/day over 60 days in STZ-induced Wistar rats significantly reduced both fasting and postprandial blood glucose, significantly improved OGTT glucose, and significantly increased fasting plasma insulin [195]. Lastly, freeze-dried grape powder at 1% dietary supplementation in NOD mice significantly reduced T1D incidence over 28 weeks [196].

FPZ (Chinese raspberry) at 30 mg/kg/day over 18 days in STZ-induced C57BL/6 mice significantly reduced fasting blood glucose, significantly increased fasting insulin and HOMA-β index, and significantly improved glucose tolerance [197]. Passion fruit peel extracts (PFAE and PFHE, both at 400 mg/kg/day over 60 days) each significantly decreased serum glucose in STZ-induced Wistar rats [198]. *P. emblica* ethyl acetate extract (EPE) at 400 mg/kg/day over 4 or 15 weeks in cyclophosphamide-accelerated NOD/ShiLtJNarl mice significantly decreased blood glucose and HbA1c and significantly increased blood insulin [199].

Partial glycemic improvement was observed in three studies. *E. officinalis* fruit juice at 1 mL/kg/day over 8 weeks in STZ-induced Wistar rats significantly reduced serum glucose and AUC glucose, though the serum insulin increase did not reach statistical significance [200]. Cornelian cherry extract (both red and yellow fruit) at 20 mg/kg/day over 14 days in STZ-induced Wistar rats significantly decreased fasting blood glucose and OGTT AUC in both fruit color groups; however, HbA1c was significantly reduced only by the red fruit extract, with no significant effect observed for the yellow fruit extract [201]. Corinthian currants at 10% w/w dietary supplementation over 4 weeks did not significantly alter blood glucose in STZ-induced Wistar rats but did produce a significant reduction in blood insulin [202].

No significant glycemic improvement was recorded in three papers. First, apple polyphenols at 0.5%, 1%, and 2% in drinking water over 10 weeks did not significantly affect blood glucose in STZ-induced Sprague Dawley rats [203]. Cranberry extract at 250 mg/kg/day over 8 weeks similarly did not significantly affect blood glucose in STZ-induced Wistar rats [204]. *Vaccinium* spp. leaf and fruit extracts (VML, VMLF, VCL, VCLF) at their respective doses over 8 weeks in STZ-induced Wistar rats showed non-significant trends toward decreased blood glucose in three of four preparations, with serum insulin not significantly differing between groups at the end of the experiment [205].

Fruit and berry polyphenol extracts demonstrated moderate and heterogeneous glycemic efficacy overall, with significant improvement confined primarily to grape-derived proanthocyanidin preparations, berry-rich extracts with documented insulin-stimulating properties, and fruit-derived preparations tested in autoimmune NOD models, while preparations evaluated predominantly for organ-protective endpoints consistently failed to achieve significant fasting glycemic reduction. The dependence of GSE’s glycemic effect on concurrent MSC co-administration further illustrates that significant outcomes within this subclass are in several instances contingent on adjunctive interventions or specific delivery contexts rather than reflecting intrinsic glycemic activity of the polyphenol extract alone.

**Oxidative Stress:** Oxidative stress outcomes were reported in 7 of the 12 studies; four (57.1%) demonstrated significant improvement across all assessed markers, two (28.6%) demonstrated partial improvement, and one (14.3%) showed no significant effect. GSE/MSC co-treatment significantly reduced serum MDA and significantly increased GPx and SOD in serum and significantly reduced pancreatic MDA and increased pancreatic GSH compared to untreated diabetic controls [194]. FPZ significantly increased hepatic SOD content, significantly reduced total superoxide and ROS generation, and significantly reduced hepatic MDA [197]. *E. officinalis* fruit juice significantly increased SOD, CAT, and GSH and significantly decreased MDA in STZ-induced Wistar rats [200]. Cranberry extract significantly decreased MDA and caspase-1 levels and significantly increased SOD and GSH in STZ-induced Wistar rats, with antioxidant benefit occurring in the absence of any glycemic effect, a dissociation of the same pattern observed in several flavonoid subclasses [204].

Partial antioxidant improvement was observed in two studies. Cornelian cherry red fruit extract significantly reduced TBARSs, oxidatively modified proteins, AGEs, and AOPPs; the yellow fruit extract significantly reduced AGEs and AOPPs and significantly increased reduced glutathione but did not significantly affect TBARSs or oxidatively modified proteins, yielding a more restricted antioxidant profile [201]. Apple polyphenols significantly reduced kidney TBARSs at 1% and 2% concentrations and significantly reduced kidney GST-π at 0.5% and 2% but did not significantly affect kidney GST or GST-α at any dose and produced no glycemic improvement, representing a renal-tissue-specific antioxidant effect in the absence of systemic glycemic activity [203]. The only paper that found no significant oxidative stress effect showed that *Vaccinium* spp. extracts did not significantly affect MDA levels at any dose or preparation tested [205]. Oxidative stress was not assessed in the EPE, passion fruit peel, Corinthian currant, grape powder, or GSPE studies [195,196,198,199,202].

Antioxidant outcomes in this subclass were modestly more consistent than glycemic outcomes, with the majority of studies reporting oxidative stress results demonstrating at least partial improvement; however, the cranberry extract finding, significant antioxidant benefit in the complete absence of glycemic effect, reinforces the now-recurrent pattern across this review that oxidative stress attenuation and glycemic correction represent mechanistically dissociable endpoints that do not co-occur reliably within a single extract or intervention. The absence of oxidative stress reporting in 5 of 12 studies, concentrated among the grape-derived and passion fruit preparations that showed the strongest glycemic effects, leaves the antioxidant characterization of this subclass substantially incomplete and limits the degree to which a unified pharmacological profile can be attributed to fruit and berry polyphenol extracts as a class.

### 7.3. Phenolic-Acid- and Tannin-Rich Extracts

Phenolic-acid- and tannin-rich extracts comprised 12 preclinical studies evaluating extracts from 12 distinct botanical sources: *Loranthus regularis*, *Trigonella foenum-graecum* L. seed methanolic extract (MEF), *Heliotropium strigosum* (Hs.Cr), *Ecklonia cava* methanol extract (ECM), *Eugenia sonderiana* hydroethanolic leaf extract, *Beta vulgaris* leaf extract, *Eugenia Uniflora* L. aqueous leaf extract, phenolic fraction concentrate from date seeds (PFC), *Plantago ovata* ethanol extract (POEE), *Excoecaria agallocha* L. ethanolic leaf extract (EAL), *Lythrum salicaria* L. ethanol extract (LSEE), and *Syzygium cumini* aqueous seed extract (AESC). STZ induction was the most common model, used in nine studies across Wistar rats (*n* = 7) and Sprague Dawley rats (*n* = 2). Alloxan-induced (*n* = 2) and NOD mice (*n* = 1) made up the remainder of studies. *Syzygium cumini* (jambul/java plum), characterized by gallic acid, ellagic acid, and anthocyanin derivatives as its principal polyphenols, has been investigated for its capacity to attenuate ER stress, inhibit NF-κB-mediated oxidative signaling, and modulate the ATF-6/CHOP axis in diabetic models [206].

**Glycemic Control:** Glycemic outcomes were reported in all 12 studies; 10 (83.33%) demonstrated significant improvement across all assessed glycemic metrics and two (16.67%) demonstrated partial improvement, with no study reporting a complete absence of glycemic effect. *L. regularis* at 150 and 300 mg/kg/day over 4 weeks significantly lowered blood glucose and increased serum insulin in STZ-induced Wistar rats in a dose-dependent manner, with the higher dose reaching a greater level of significant glucose reduction [207]. Similarly dose-dependent, Hs.Cr extract at 100, 300, 1000, and 3000 mg/kg/day in alloxan-induced Sprague Dawley rats, *Beta vulgaris* leaf extract at 50, 100, and 200 mg/kg/day in alloxan-induced Wistar rats, and EAL at 250 and 500 mg/kg/day in STZ-induced Wistar rats all produced significant reductions in blood glucose, with the higher doses correlating to more significant reductions [208,209,210]. Interestingly, LSEE at all three concentrations (100%, 50%, 25%) significantly reduced blood glucose in STZ-induced Wistar rats over 10 days, but the 50% preparation had the greatest reduction [211].

Next, MEF (*T. foenum-graecum*) at 400 mg/kg/day over 28 days significantly decreased fasting blood glucose in STZ-induced Wistar rats [212]. ECM (*E. cava*) at 300 mg/kg/day over 3 weeks significantly lowered blood glucose and significantly increased serum insulin in STZ-induced Sprague Dawley rats [213]. *E. uniflora* aqueous leaf extract administered ad libitum in drinking water over 22 weeks in spontaneous NOD mice significantly lowered blood glucose and GTT AUC, significantly increased serum insulin, and reduced diabetes incidence [214]. AESC at 250 mg/kg/day over 4 weeks significantly reduced fasting blood glucose and reduced OGTT AUC in STZ-induced Sprague Dawley rats [215]. Lastly, PFC from date seeds at 50 mg/kg/day over 12 weeks in STZ-induced Wistar rats significantly reduced fasting blood glucose when co-administered with insulin, suggesting PFC exerts an adjunctive rather than independent glycemic effect [216].

Partial glycemic improvement was observed in two studies: *E. sonderiana* extract at 200 mg/mL/kg over 90 days in STZ-induced Wistar rats significantly reduced fasting glucose, FCM, and HbA1c but did not significantly reduce AGE levels, and POEE at the highest concentration (100%) significantly reduced blood glucose comparable to metformin; but the 50% and 25% preparations failed to produce significant reductions [217,218].

Phenolic-acid- and tannin-rich extracts demonstrated the highest rate of glycemic efficacy seen so far in this review, with all 12 studies reporting at least partial glycemic improvement and the large majority achieving significant reduction across all assessed metrics. A recurring dose-dependence pattern, evident across *L. regularis*, Hs.Cr, *Beta vulgaris,* EAL, and POEE, and the adjunctive-only glycemic activity of PFC suggest that, within this subclass, glycemic efficacy is frequently threshold-dependent and, in at least one instance, contingent on co-administration with exogenous insulin rather than reflecting autonomous hypoglycemic activity.

**Oxidative Stress:** Oxidative stress outcomes were reported in 9 of the 12 studies; seven (77.8%) demonstrated significant improvement across all assessed markers and two (22.2%) demonstrated partial improvement, with no study reporting a complete absence of antioxidant effect among those reporting this outcome. EAL demonstrated the broadest tissue-level antioxidant coverage, significantly increasing SOD, CAT, and GSH and significantly reducing MDA across kidney, heart, and liver tissue in STZ-induced Wistar rats at both 250 and 500 mg/kg/day [210]. *E. sonderiana* extract significantly reduced liver and kidney MDA and significantly increased SOD, GPx, and CAT in both organs [218]. *Beta vulgaris* extract significantly reduced hepatic MDA and significantly increased hepatic total antioxidant capacity (TAO) and GSH and additionally significantly reduced serum TNF-α, IL-1β, and IL-6 and downregulated hepatic NF-κB expression, providing evidence for coupled antioxidant and anti-inflammatory signaling [209]. *L. regularis* significantly decreased TBARSs and significantly increased GSH, SOD, CAT, GR, GPx, and GST in a dose-dependent manner in STZ-induced Wistar rats, similar to its dose-dependent effect for blood glucose [207]. POEE also had dose-specific effects, as, at 100% and 50% concentrations, POEE significantly reduced TOS, OSI, MDA, AOPP, 8-OHdG, NOx, and 3-NT and significantly increased TAC and SH groups while the 25% preparation achieved significant reductions in MDA and 3-NT and significant increases in TAC but did not significantly affect AOPPs, 8-OHdG, AGEs, NOx, or SH at this dose [217]. Next, AESC significantly restored SOD, CAT, and GSH and significantly reduced MDA in STZ-induced Sprague Dawley rats [215]. Lastly, *E. uniflora* extract significantly increased hepatic GSH and significantly reduced serum TBARSs in NOD mice over 22 weeks [214].

Partial antioxidant improvement was observed in two studies. Date seed phenolics (PFC) co-administered with insulin significantly enhanced CAT and SOD activity and reduced MDA and NO across brain regions and peripheral organs but, when compared directly to the insulin-only group, the additive effect of PFC reached significance for MDA (except hippocampus) and NO (except striatum) and for CAT in most but not all brain regions, indicating region-specific rather than uniform augmentation of antioxidant capacity [216]. LSEE significantly reduced TOS, OSI, MDA, AOPPs, and 3-NT across all three concentrations and significantly reduced 8-OHdG at 50% and 100%; however, LSEE had no significant effect on NOx at any concentration, and TAC was significantly increased only at 100% and 25% but not 50%, representing incomplete restoration of the full oxidative stress panel [211]. Oxidative stress was not assessed in the MEF, Hr.Cr, or ECM studies [208,212,213].

Phenolic-acid- and tannin-rich extracts demonstrated the most consistently favorable antioxidant profile of the four natural extract subclasses, with all nine studies reporting oxidative stress outcomes achieving at least partial improvement and the majority achieving complete restoration across all assessed markers, a finding that aligns with the well-characterized radical-scavenging capacity of gallic acid, ellagic acid, and tannin-derived compounds that dominate this subclass’s polyphenol profile. The dose dependence of antioxidant efficacy observed for *L. regularis* and POEE and the region-specific rather than uniform antioxidant augmentation seen with PFC further reinforce that, even within a subclass with high overall antioxidant success rates, the magnitude and breadth of effect remain sensitive to dosing parameters and tissue-specific bioavailability.

### 7.4. Other Plant Extracts

The other plant extracts subclass comprised 15 preclinical studies evaluating botanicals that did not fit within the preceding polyphenol-defined categories, including extracts with mixed or uncharacterized polyphenol profiles, leaf-derived preparations from fruit-bearing plants, and multi-constituent preparations. Compounds evaluated were *Cuscuta chinensis* Lam. mixed plant phenolics (*n* = 2), *Anacardium occidentale* hexane leaf extract, stevia leaves, *Passiflora alata* aqueous leaf extract, *Bauhinia variegata* alcoholic leaf extract, *Moringa oleifera* leaves, Mongolian oak cup ethanol crude extract (ECE), *Ilex paraguariensis* (yerba mate) aqueous extract, okra (*Abelmoschus esculentus*) pod extract, olive leaf powder, yacon leaf extract (*Smallanthus sonchifolius*), Si Wei Jiang Huang Tang San (SWJHTS), thyme honey and olive oil combination, and *Hibiscus trionum* tea (HTT). STZ induction was used in nine studies across Wistar rats (*n* = 5), Sprague Dawley rats (*n* = 1), ICR mice (*n* = 1), and C57BL/6N mice (*n* = 1); alloxan induction was employed in five studies utilizing *Rattus rattus* (*n* = 2), Wistar rats (*n* = 1), Sprague Dawley rats (*n* = 1), and albino rabbits (*n* = 1); and a spontaneous NOD mouse model was used in one study. *I. paraguariensis* (yerba mate), notable for its chlorogenic acid and rutin content, has been characterized for neuroprotective and antioxidant properties in diabetic models, including attenuation of peripheral neuropathy and multi-organ lipid peroxidation in STZ-induced rodents [219].

**Glycemic Control:** Glycemic outcomes were reported in all 15 studies; 13 (86.7%) demonstrated significant improvement across all assessed glycemic metrics and two (13.3%) demonstrated partial improvement, with no study reporting a complete absence of glycemic effect. *Cuscuta chinensis* extract at 400 mg/kg in alloxan-induced *Rattus rattus* significantly decreased fasting blood glucose across all time points in both independent studies, with the 2019 study by the same group documenting progressive reduction to near-normoglycemic levels by 90 days [220,221]. *H. trionum* tea (HTT) at 8.25 g/kg/day over 4 weeks significantly reduced blood glucose and significantly increased serum insulin in STZ-induced Wistar rats [222]. Yacon leaf extract at 100 mg/kg/day over 30 days significantly reduced glycemia and significantly increased serum insulin concentration in STZ-induced Wistar rats [223]. *I. paraguariensis* extract at 850 mg/kg/day over 12 weeks significantly reduced blood glucose and serum fructosamine in STZ-induced Swiss mice [224]. Okra pod extract at 400 mg/kg/day over 4 weeks significantly lowered blood glucose and HbA1c in STZ-induced Wistar rats [225]. *B. variegata* alcoholic leaf extract at 250, 500, and 1000 mg/kg/day significantly decreased blood glucose in a dose-dependent manner and significantly reduced OGTT blood glucose in STZ-induced Sprague Dawley rats over 28 days [226]. Stevia leaf polyphenol preparation significantly decreased blood glucose and significantly increased serum insulin and significantly improved IPGTT and IPITT blood glucose at multiple time points in STZ-induced Wistar rats [227]. Olive leaf powder at 0.3% and 0.6% *w*/*w* in the diet significantly reduced fasting blood glucose and significantly increased serum insulin in all dose groups in STZ-induced ICR mice over 4 weeks [228]. *M. oleifera* leaves at 100, 200, and 400 mg/kg/day significantly reduced blood glucose at all doses in alloxan-induced albino rabbits over 14 days [229]. SWJHTS at 225 and 112.5 mg/kg/day significantly reduced fasting blood glucose and significantly reduced OGTT AUC at the optimal dose in STZ-induced C57BL/6N mice over 7 days [230]. Thyme honey and olive oil each independently significantly decreased blood glucose in alloxan-induced Wistar rats over 30 days [231]. Lastly, *P. alata* aqueous leaf extract at 15 g leaves/L in drinking water over 30 weeks significantly decreased insulin AUC and glucose AUC in NOD mice [232].

Partial glycemic improvement was observed in two studies. *A. occidentale* hexane extract at 300 mg/kg/day significantly decreased fasting blood glucose, urinary protein, albuminuria, glycosuria, and urinary urea in STZ-induced Wistar rats, while 150 mg/kg/day achieved significance only for glycosuria reduction, indicating dose-dependent efficacy with incomplete glycemic correction at the lower dose [233]. Another dose-threshold effect was noted in the use of Mongolian oak cup extract (ECE), where 800 mg/kg/day significantly reduced fasting blood glucose from day 2 through day 10 in alloxan-induced Sprague Dawley rats, but 200 mg/kg/day did not achieve significance [234].

Other plant extracts demonstrated a very high rate of glycemic efficacy, and when combined with the previous section of phenolic-acid- and tannin-rich extracts, represent compounds that had the highest glycemic reduction power. The efficacy of this group likely reflects the broad mechanistic diversity of this subclass, which encompasses extracts acting through complementary pathways including α-glucosidase inhibition, insulin secretagogue activity, immune modulation in autoimmune models, and direct β-cell preservation. The dose-threshold effects observed in both *A. occidentale* and ECE further reinforce the cross-subclass pattern that glycemic efficacy within polyphenol-rich botanical extracts is frequently non-linear with respect to dose, with subthreshold preparations producing incomplete or absent glycemic correction despite the same underlying polyphenol composition.

**Oxidative Stress:** Oxidative stress outcomes were reported in 8 of the 15 studies; six (75%) demonstrated significant improvement across all assessed markers and two (25%) demonstrated partial improvement, with no study showing a complete absence of antioxidant effect among those reporting this outcome. *I. paraguariensis* extract significantly reduced TBARSs in liver, kidney, and brain and normalized NPSH across all three organs, while SOD activity was significantly reduced in liver and kidney and CAT activity was significantly reduced in liver and brain [224]. Yacon leaf extract significantly increased CAT, SOD, and GPx activities and significantly decreased MDA in STZ-induced Wistar rats [223]. HTT significantly increased plasma, muscle, kidney, and liver SOD and plasma and multi-tissue GPx and significantly reduced MDA across plasma, heart, skeletal muscle, liver, and kidney [222]. ECE at 800 mg/kg/day significantly decreased MDA in heart tissue and significantly recovered SOD in heart, kidney, spleen, and liver; however, GSH levels were significantly reduced in heart tissue with ECE treatment, a finding the authors noted may reflect increased GSH utilization under active oxidative scavenging [234]. *P. Alata* leaf extract significantly increased FRAP and significantly decreased TBARSs in NOD mice, indicating concurrent improvement in total antioxidant capacity and attenuation of lipid peroxidation [232]. Lastly, thyme honey and olive oil significantly restored CAT, GSH, and GPx toward normal and reduced MDA in kidney, pancreas and liver enzymatic antioxidants [231].

Partial antioxidant improvement was observed in two studies. Stevia polyphenols did not significantly affect liver TBARSs but significantly reduced liver hydroperoxides and conjugated dienes and significantly increased liver SOD and CAT, suggesting selective attenuation of non-MDA lipid peroxidation products and enzymatic antioxidant restoration without significant reduction in MDA equivalents [227]. Olive leaf powder significantly increased GPx activity at 0.3% and 0.6% and tended to increase SOD and CAT; however, NO levels did not significantly decrease, and iNOS mRNA expression was significantly reduced only at the lowest dose (0.3%), indicating incomplete resolution of reactive nitrogen species generation [228]. Oxidative stress was not assessed in *C. chinensis*, *A. occidentale*, *B. variegata*, *M. oleifera*, okra, or SWJHTS studies [220,221,225,226,229,230,233].

Antioxidant efficacy within the other plant extracts subclass was consistently positive among studies reporting this outcome, with all eight demonstrating at least partial improvement across assessed markers and the majority achieving complete restoration—a pattern that, combined with the near-universal glycemic efficacy of this subclass, suggests that botanicals in this category exert broad-spectrum activity across both primary outcome domains despite their chemically heterogeneous polyphenol profiles. The substantial proportion of studies not reporting oxidative stress outcomes (46.67%), concentrated among extracts evaluated primarily for glycemic or organ-specific endpoints, represents the most pronounced evidence gap in this subclass along with flavonoid-rich extracts and prevents a comprehensive assessment of whether the strong glycemic efficacy observed here is accompanied by commensurate antioxidant activity across the full extract set.

## 8. Other Polyphenols

Beyond the flavonoid class, this review identified a heterogeneous group of 13 studies examining polyphenolic compounds from four structurally distinct non-flavonoid classes: chalcones and dihydrochalcones (*n* = 5), phenolic acids (*n* = 4), ellagitannins (*n* = 2), and lignans (*n* = 2). While each class shares the common polyphenolic feature of one or more aromatic rings bearing hydroxyl substituents, they are distinguished by their core carbon scaffolds, degree of polymerization, and biosynthetic origins [235]. All 13 studies utilized STZ-induced T1D models across three rodent strains: Wistar rats (*n* = 7), C57BL/6 mice (*n* = 4), and Sprague Dawley rats (*n* = 2). Given the structural heterogeneity of this group and the limited number of studies per class, findings are organized by polyphenol class below.

### 8.1. Chalcones and Dihydrochalcones

Chalcones are biosynthetic precursors of the flavonoid family, characterized by an open-chain α,β-unsaturated ketone scaffold in which two aromatic rings (rings A and B) are connected by a three-carbon alkenone unit; dihydrochalcones represent the reduced form of this scaffold, bearing a saturated three-carbon alkanone bridge in place of the α,β-unsaturated bond [236]. This group was represented by five preclinical studies examining four distinct compounds: the synthetic chalcone L2H17 (*n* = 1), trans-chalcone (*n* = 1), isoliquiritigenin (*n* = 1), phloretin (*n* = 1), and its metabolite phloretamide (*n* = 1). All five studies utilized STZ induction across Wistar rats (*n* = 3) and C57BL/6 mice (*n* = 2). Phloretin, the most pharmacologically characterized dihydrochalcone in this review, is found naturally in apple peel and has been identified as a TLR-4 inhibitor and SGLT2 co-inhibitor, with established anti-inflammatory and renoprotective properties independent of glycemic lowering [237].

**Glycemic Control:** Glycemic outcomes were reported in all five chalcone and dihydrochalcone studies; of these, three demonstrated significant improvement and one demonstrated absent glycemic effect, with one study not designed to assess glycemic endpoints. Trans-chalcone demonstrated the most comprehensive glycemic activity, significantly reducing blood glucose levels and increasing serum insulin at doses of 2–32 mg/kg/day over 24 days in STZ-induced Wistar rats, though the authors noted a paradoxical increase in triglycerides and VLDL at the highest dose of 32 mg/kg [238]. Phloretamide significantly reduced fasting plasma glucose and serum insulin at 200 mg/kg twice weekly over 12 weeks in STZ-induced Wistar rats [239]. Phloretin significantly lowered blood glucose at 50 and 100 mg/kg administered orally over 4 days in STZ-induced Wistar rats, in the context of an acute renal ischemia model in which the primary endpoint was nephroprotection rather than chronic glycemic management [240]. L2H17, a synthetic chalcone derivative administered at 20 mg/kg/day over 8 weeks in STZ-induced C57BL/6 mice, did not significantly affect blood glucose levels, consistent with a primary mechanistic target of inflammatory pathway inhibition rather than glucose metabolism in that model [240,241]. Glycemic outcomes were not reported for isoliquiritigenin, which was designed exclusively to assess renal oxidative injury [242].

**Oxidative Stress:** Oxidative stress outcomes were reported in only two of the five chalcone and dihydrochalcone studies; oxidative stress was not assessed in the L2H17, phloretin, or trans-chalcone studies, representing a notable gap in the oxidative characterization of this subclass [238,240,241]. Phloretamide significantly reduced renal MDA and advanced glycation end products (AGEs) and significantly increased renal GSH, SOD, and HO-1 in STZ-induced Wistar rats at 200 mg/kg, demonstrating a multi-target antioxidant profile concentrated in renal tissue [239]. Isoliquiritigenin produced significant antioxidant effects across multiple molecular targets in renal tissue of STZ-induced C57BL/6 mice at 10–20 mg/kg every other day over 12 weeks, significantly preventing the hyperglycemia-induced decrease in renal SOD activity, reducing O_2_^−^ production as demonstrated by DHE staining, and significantly upregulating both Nrf2 mRNA and protein expression alongside HO-1 mRNA, in the complete absence of glycemic reporting, reflecting a pattern of selective antioxidant organ protection without glycemic assessment observed previously in this review [242].

### 8.2. Phenolic Acids

Phenolic acids are the structurally simplest class of dietary polyphenols, defined by a phenolic ring bearing at least one carboxylic acid functional group, and are conventionally subdivided into hydroxybenzoic acids (C6–C1 scaffold) and hydroxycinnamic acids (C6–C3 scaffold) based on the point of attachment of the carboxyl group relative to the aromatic ring [235]. This subclass was represented by four preclinical studies examining four distinct compounds: syringaldehyde (SA), caffeic acid phenethyl ester (CAPE) and its synthetic analogue VP961, protocatechuic acid (PCA), and 3′,4′-dihydroxyphenylglycol (DHPG). All four studies utilized STZ induction, with an even split between Wistar rats (*n* = 2) and Sprague Dawley rats (*n* = 2) as the experimental models. DHPG, the most structurally unique phenolic acid in this group as a hydroxyethyl catechol derivative found in extra virgin olive oil, has been characterized for its capacity to attenuate systemic and renal oxidative burden through combined lipid peroxidation suppression and glutathione restoration [243].

**Glycemic Control:** Glycemic outcomes were reported in all four phenolic acid studies, with significant improvement in all markers demonstrated in three (75%) and partial improvement in one (25%). Syringaldehyde significantly decreased blood glucose at 5 and 20 mg/kg over 8 weeks in STZ-induced Sprague Dawley rats, with significant increases in serum insulin at both doses, effects mechanistically attributed to GLP-1 receptor agonism [244]. CAPE and its structural analogue VP961, both administered at 30 mg/kg over 21 days in STZ-induced Wistar rats, produced significant reductions in blood glucose at days 8, 15, and 21, alongside significant increases in plasma insulin; notably, both the natural compound and synthetic analogue demonstrated statistically equivalent glycemic efficacy, supporting CAPE’s ester bond as non-essential for this activity [245]. DHPG significantly reduced blood glucose in both the 0.5 and 1 mg/kg/day groups over 2 months in STZ-induced Wistar rats [246]. Lastly, PCA significantly decreased fasting blood glucose at both 50 and 100 mg/kg/day and significantly reduced plasma HbA1c, but plasma insulin was not significantly altered at either dose, suggesting glycemic improvement through peripheral mechanisms independent of pancreatic insulin secretion enhancement [247].

**Oxidative Stress:** Oxidative stress outcomes were reported in three of the four phenolic acid studies; with only syringaldehyde not reporting any oxidative stress markers [244]. All three reporting studies demonstrated significant improvement in all assessed markers (100%). CAPE and VP961 both significantly reduced plasma and pancreatic lipid hydroperoxide (LOOH) concentrations, significantly increased plasma and pancreatic reactive sulfhydryl groups (RSH), and significantly upregulated pancreatic HO-1 and γ-glutamylcysteine ligase (GGCL) protein expression, with equivalence observed between the natural compound and its synthetic analogue across all oxidative stress endpoints [245]. PCA significantly reduced cardiac MDA at both 50 and 100 mg/kg at week 12 and significantly attenuated cardiac mitochondrial ROS production at both doses, situating this compound’s antioxidant activity specifically within the myocardial mitochondrial compartment in the context of diabetic cardiomyopathy [247]. DHPG produced the most comprehensive systemic and organ-specific antioxidant profile in this subclass, significantly reducing both serum and renal TBARSs, 8-OHdG, oxLDL, and 3-nitrotyrosine, while significantly increasing serum and renal GSH and total antioxidant capacity (TAC), and significantly reducing urinary 8-isoprostane at both 0.5 and 1 mg/kg/day, demonstrating coordinated attenuation of lipid peroxidation, protein nitration, DNA oxidation, and glutathione depletion across systemic and renal compartments simultaneously [246].

### 8.3. Ellagitannins

Ellagitannins are high-molecular-weight hydrolysable tannins formed by the oxidative C–C linkage of galloyl groups esterified to a polyol core, typically D-glucose, which upon hydrolysis yield hexahydroxydiphenic acid that spontaneously lactonizes to ellagic acid; they represent the largest subgroup of the hydrolysable tannin class and are distinguished from gallotannins by the presence of these C–C-linked hexahydroxydiphenoyl (HHDP) units [248]. This subclass was represented by two studies examining two distinct compounds: ellagic acid (EA) and punicalagin. Both studies utilized STZ-induced Wistar rats. Punicalagin, the most widely studied ellagitannin in the broader literature, is the principal polyphenolic constituent of pomegranate (*Punica granatum*) and has been characterized for its potent antioxidant, anti-inflammatory, and pancreatic cytoprotective properties, with hydrolytic release of ellagic acid underlying a portion of its in vivo biological activity [249].

**Glycemic Control:** Glycemic outcomes were reported in both ellagitannin studies, with significant improvement in all markers in both. EA significantly reduced fasting glucose and significantly increased fasting insulin at 50 mg/kg/day over 12 weeks in STZ-induced Wistar rats targeting hepatic injury [250]. Punicalagin at the notably low dose of 1 mg/kg/day over 15 days significantly decreased blood glucose and significantly increased serum insulin, representing the shortest intervention duration and lowest effective dose producing dual glycemic and insulinotropic effects across the entire other polyphenols group [251].

**Oxidative Stress:** Oxidative stress outcomes were also reported in both ellagitannin studies with significant improvement in all markers in both. EA significantly reduced hepatic ROS and hepatic MDA, significantly increased hepatic GSH and hepatic SOD, and significantly increased nuclear Nrf2 activity 4-fold in the liver, with effects concentrated within the hepatic compartment in the context of non-alcoholic fatty liver disease pathology [250]. Punicalagin demonstrated pancreatic-targeted antioxidant activity, significantly reducing pancreatic MDA and protein carbonyl (PC) content, significantly increasing pancreatic GSH, GPx, GR, SOD, and CAT, and significantly increasing serum paraoxonase-1 (PON-1) activity, indicating simultaneous restoration of enzymatic antioxidant defenses at the level of the pancreatic islet and systemic antioxidant capacity [251]. Taken together, these two studies present complementary organ-specific profiles: EA concentrated effects within hepatic tissue via Nrf2 activation, while punicalagin demonstrated comprehensive enzymatic antioxidant restoration within pancreatic tissue alongside systemic PON-1 upregulation.

### 8.4. Lignans

Lignans are a class of plant phenolic compounds derived from the oxidative coupling of two phenylpropanoid (C6–C3) units linked by a central C8–C8′ bond, forming a characteristic dibenzylbutane or dibenzylbutyrolactone skeleton, features that confer a degree of structural similarity to endogenous estrogens and enable both hormonal and redox-modulatory activities [252]. This subclass was represented by two studies, both examining syringaresinol (SYR) as the sole compound. Both studies utilized STZ-induced C57BL/6 mice administered SYR at 25 mg/kg, with one study dosing three times weekly for 8 weeks targeting diabetic retinopathy [253] and the other dosing every other day for 8 weeks targeting diabetic cardiomyopathy [254]. Syringaresinol, found in cereals and various medicinal plants including ginseng berry, has been extensively characterized for its capacity to suppress the Keap1/Nrf2 axis and downstream targets including HO-1, NQO-1, and SOD2 across multiple diabetic organ injury models [255].

**Glycemic Control:** Glycemic outcomes were reported in both syringaresinol studies, with discordant results across target organs. In the retinopathy model, SYR significantly attenuated fasting blood glucose elevation over 8 weeks [253]. In the cardiomyopathy model, SYR did not significantly affect blood glucose or glucose tolerance relative to untreated diabetic controls, despite producing robust cardiac and antioxidant effects in the same animals [255]. This glycemic dissociation across two studies of the same compound at the same dose and duration, differing only in dosing frequency, suggests that the antioxidant and organ-protective effects of SYR are mechanistically separable from any effect on systemic glucose homeostasis.

**Oxidative Stress:** Oxidative stress outcomes were reported in both syringaresinol studies, with significant improvement in all assessed markers in both (100%). In the retinopathy model, SYR significantly reduced retinal ROS generation and serum ROS and H_2_O_2_ and significantly upregulated retinal Nrf2 protein and mRNA, HO-1, and SOD2 [253]. In the cardiomyopathy model, SYR significantly reduced myocardial ROS generation, significantly increased cardiac Nrf2 mRNA and protein expression, significantly increased SOD protein expression and HO-1 mRNA, significantly downregulated Keap1 protein, and significantly upregulated NQO-1 mRNA [255]. The Nrf2/Keap1/HO-1/NQO-1 pathway was activated in both target organs, establishing Nrf2 induction as the consistent mechanistic signature of SYR across retinal and myocardial T1D injury models. The glycemic dissociation observed in the cardiomyopathy study, significant antioxidant protection without any glycemic improvement, reinforces this pathway as the primary therapeutic mechanism rather than secondary to glucose normalization.

## 9. Discussion

This systematic review summarizes the available preclinical evidence on polyphenols in T1D models. The findings suggest potential effects on redox-related processes relevant to T1D, although the consistency and magnitude of these effects varied across compounds, formulations, doses, durations, and disease models. The Discussion focuses on broader themes that may be relevant for translation, including oxidative stress and glycemic outcomes, redox-sensitive mechanisms, bioavailability, model selection, and future research priorities.

## 10. Glycemic and Antioxidant Effect Dissociation

A recurring pattern across the included studies was that changes in oxidative stress markers did not always parallel changes in glycemic outcomes. In several studies, improvements in lipid peroxidation, antioxidant enzyme activity, or redox-sensitive signaling were reported even when fasting glucose, insulin, or HbA1c showed limited or no improvement. This suggests that redox-related effects may occur independently of measurable glycemic changes in preclinical T1D models.

The observation that antioxidant effects may occur without parallel glucose-lowering effects is relevant for translation. In T1D, insulin remains essential for glycemic management, while oxidative stress may continue to contribute to β-cell vulnerability and diabetic complications. Therefore, polyphenols may be more appropriately investigated as adjunctive strategies targeting oxidative tissue injury and redox imbalance, rather than as stand-alone glucose-lowering interventions.

## 11. Bioavailability and Translational Constraints

Bioavailability remains an important consideration when interpreting the variable glycemic effects observed across studies. For several polyphenols, limited absorption, rapid metabolism, short systemic half-life, and tissue-specific distribution may reduce the likelihood that experimental effects translate directly to clinically achievable exposures [256]. Differences in formulation and delivery route may also partly explain why some studies reported stronger glycemic or antioxidant effects than others.

Future studies should therefore include pharmacokinetic and pharmacodynamic assessment alongside efficacy outcomes. In particular, studies should clarify whether the doses used produce tissue concentrations relevant to human use and whether enhanced-delivery formulations improve target engagement without introducing safety concerns. Addressing these issues would help determine whether inconsistent glycemic effects reflect limited biological activity, insufficient exposure, or differences in experimental design.

## 12. Model Selection and Translational Relevance

The predominance of STZ-induced models represents an important constraint on the mechanistic and translational interpretation of the current evidence. In this review, STZ-induced rodent models accounted for 83.25% of included studies and therefore served as the main experimental context for evaluating polyphenol effects. STZ produces diabetes through rapid chemical β-cell injury involving DNA alkylation, poly(ADP-ribose) polymerase activation, NAD^+^/ATP depletion, oxidative stress, and β-cell necrosis. This model is useful for studying acute β-cell toxicity, hyperglycemia-associated oxidative stress, and secondary tissue injury, but it does not reproduce the autoimmune pathogenesis that defines human T1D.

This limitation is especially relevant for interpreting outcomes related to immunomodulation and β-cell preservation. Human T1D develops through chronic, antigen-specific immune responses involving insulitis, autoreactive T-cell activation, antigen-presenting cell function, inflammatory cytokine signaling, and progressive loss of immune tolerance. Therefore, improvements in oxidative stress markers, insulin secretion, glycemic parameters, or islet morphology in STZ-based studies should be interpreted primarily as evidence of cytoprotection in a chemically induced injury model. These outcomes do not, by themselves, establish that polyphenol suppresses autoimmune β-cell targeting or modifies the underlying disease process.

Future mechanistic studies should prioritize spontaneous and immune-driven models when evaluating polyphenols for T1D prevention, immunomodulation, or β-cell preservation. The NOD mouse model, used in only 12 studies in this review, provides a more immunologically relevant platform because it develops spontaneous insulitis, progressive immune-mediated β-cell loss, and T-cell-dependent diabetes. Humanized autoimmune diabetes models may further strengthen translational relevance by enabling assessment of human immune components. Studies using these models should include direct measures of insulitis severity, autoreactive effector T-cell activity, regulatory T-cell function, cytokine networks, β-cell antigen-specific immune responses, and sustained endogenous insulin production. This approach would help distinguish general antioxidant or cytoprotective effects from autoimmune disease-modifying activity in T1D.

## 13. Compound-Level Heterogeneity

Across the included studies, compounds within the same polyphenol subclass did not always show similar effects on glycemic outcomes or oxidative stress markers. Although subclass groupings such as flavonoids, stilbenes, diarylheptanoids, natural extracts, and other polyphenols were useful for organizing the evidence, they should not be interpreted as reliable predictors of efficacy. Differences in response may reflect compound-specific properties, formulation, dose, treatment duration, disease model, tissue target, and outcome selection.

These patterns suggest that broad subclass-level conclusions should be made cautiously. Future studies may be more informative if they focus on individual compounds or defined formulations, with attention to reproducibility across relevant models, evidence of target engagement, and clinically achievable exposure levels. This approach may help identify which polyphenols warrant further translational evaluation in T1D.

## 14. Dose and Duration Considerations

Differences in dose and treatment duration may partly account for the variability in glycemic and oxidative stress outcomes across studies. Polyphenol effects are likely influenced by exposure-dependent factors, including absorption, metabolism, tissue distribution, and the time required to induce downstream changes in antioxidant defenses or tissue injury markers. As a result, short-duration studies or single-dose designs may not adequately capture delayed or threshold-dependent effects, particularly for outcomes related to antioxidant enzyme activity, mitochondrial function, inflammation, or diabetic complications.

Future preclinical studies should incorporate dose–response assessment and longitudinal follow-up to better define the relationship between polyphenol exposure, pathway engagement, and biological effect. Reporting administered dose alone may be insufficient; where feasible, studies should also include pharmacokinetic measures, tissue exposure data, and time-course analyses of glycemic and oxidative stress endpoints. These design elements would improve comparability across studies and help determine whether inconsistent findings reflect true lack of efficacy, inadequate exposure, or insufficient treatment duration.

## 15. Oxidative Stress Endpoint Selection

Oxidative stress endpoints varied substantially across the included studies. Commonly reported markers, including MDA, SOD, CAT, GSH, and GPx, are useful for assessing lipid peroxidation and antioxidant defense capacity [257]. However, these endpoints provide limited information about the cellular source of oxidative stress, the subcellular compartment involved, or the specific signaling pathways through which polyphenols may exert redox-related effects.

Future studies should pair conventional oxidative stress biomarkers with more mechanistically informative endpoints. These may include measures of Nrf2 nuclear translocation, Keap1 regulation, HO-1 and NQO1 expression, mitochondrial ROS production, NADPH oxidase activity, protein oxidation, DNA damage, and inflammatory redox signaling. Incorporating pathway- and compartment-specific measures would allow clearer distinction between general antioxidant effects and targeted modulation of redox signaling, thereby improving mechanistic interpretation and comparability across studies.

## 16. Evidence Gaps and Future Directions

Several evidence gaps were identified across the included studies and should be prioritized in future research. A major limitation was the inconsistent reporting of oxidative stress outcomes across all major polyphenol categories, including flavonoids, stilbenes, diarylheptanoids, natural extracts, and other polyphenols. This gap was especially apparent in studies focused on organ-specific or secondary outcomes. As oxidative stress is central to the rationale for evaluating polyphenols in T1D, incomplete reporting limits comparison of antioxidant effects across compounds, subclasses, and experimental models.

A further gap is the limited depth of preclinical evidence for several individual polyphenols. Although compounds such as quercetin, resveratrol, and curcumin were examined across multiple studies, many other polyphenols were represented by only one to three studies. Consequently, conclusions regarding these less frequently studied compounds should be interpreted cautiously, particularly when favorable effects are derived from isolated experiments rather than independently replicated findings. This limitation is compounded by substantial methodological heterogeneity across studies, including differences in animal species and strain, T1D induction model, route of administration, dose, formulation, treatment duration, and outcome selection. Such variability makes it difficult to determine whether divergent findings reflect true compound-specific biological effects or differences in experimental design and exposure. Future studies should prioritize replication of promising but sparsely studied polyphenols using standardized preclinical protocols, clearly defined animal models, harmonized glycemic and oxidative stress endpoints, and transparent reporting of dose, route, formulation, and T1D induction strategy.

Interpretation of the current evidence is also limited by the infrequent assessment of sex as a biological variable. Most studies used male animals, whereas female-only studies were less common, and few studies were designed to assess sex-specific responses. This is relevant because autoimmune T1D can show sex-dependent differences, and some included studies suggested that treatment effects may differ between males and females. Future preclinical studies should include both sexes whenever feasible and should be adequately powered for sex-stratified analysis.

Combination therapy represents another important area for future study. Several studies reported glycemic benefits only when polyphenols were administered with insulin or other adjunctive interventions. This is clinically relevant because individuals with T1D require insulin therapy, yet most preclinical studies did not formally determine whether combination effects were additive, synergistic, or dependent on one component of treatment. Future studies should use experimental designs that distinguish the independent and combined effects of polyphenols, insulin, and other candidate therapies. Factorial designs would allow direct comparison of each intervention alone and in combination, while dose-combination matrices could define how varying doses influence the magnitude of response. Isobolographic analysis may further determine whether observed combination effects are additive, synergistic, or antagonistic. These approaches would clarify whether polyphenols provide incremental benefit beyond insulin therapy and help define their potential role as adjunctive interventions in T1D.

Rigorous assessment of combination therapy also depends on consistency in endpoint selection and mechanistic evaluation. Across the included studies, substantial heterogeneity in outcome measures, oxidative stress biomarkers, and pathway-specific endpoints limited direct comparison and reduced the feasibility of quantitative synthesis. Future preclinical studies would benefit from a standardized minimum endpoint panel that includes conventional oxidative stress markers, pathway-specific redox measures, and disease-relevant metabolic or tissue injury outcomes. Applying a consistent endpoint framework would improve comparability across studies, strengthen mechanistic interpretation, and support more evidence-based selection of polyphenols for translational evaluation in T1D.

## 17. Conclusions

This systematic review provides an integrated evaluation of dietary polyphenols as modulators of oxidative stress and metabolic dysfunction in T1D. Across diverse compound classes and experimental systems, the evidence converges on a central finding: polyphenols exert consistent cytoprotective effects through redox regulation, whereas their influence on glycemic control remains variable and highly context-dependent.

The antioxidant effects observed are mechanistically coherent and biologically plausible. Polyphenols consistently attenuate oxidative damage and enhance endogenous defense systems, frequently through activation of the Nrf2/Keap1/ARE axis, suppression of NF-κB-mediated inflammatory signaling, and modulation of mitochondrial redox homeostasis. These actions translate into broad tissue-level protection, including preservation of pancreatic, renal, cardiovascular, and neural integrity. Importantly, these effects are often observed independently of changes in glycemia, indicating that modulation of oxidative stress represents a primary therapeutic pathway rather than a secondary consequence of improved glucose control.

In contrast, glycemic outcomes do not demonstrate uniformity across compounds or subclasses. Glucose-lowering effects, when present, are influenced by factors such as compound structure, dosing strategy, formulation, treatment duration, and underlying disease model. This heterogeneity suggests that glycemic regulation is not an inherent property of polyphenols as a class but rather a compound-specific effect that may depend on pharmacokinetic and experimental variables. Consequently, the metabolic impact of polyphenols should not be generalized without consideration of these modifying factors.

The observed dissociation between redox modulation and glycemic control has important implications for therapeutic positioning. Given the central role of oxidative stress in β-cell vulnerability and the progression of diabetic complications, polyphenols may be more appropriately conceptualized as adjunctive agents targeting downstream pathophysiology rather than primary glucose-lowering therapies. Their capacity to mitigate oxidative injury across multiple organ systems supports a potential role in slowing disease progression and reducing complication burden. However, interpretation of these findings is limited by the predominance of preclinical models, which do not fully recapitulate the immunological and metabolic complexity of human T1D. Additional constraints include heterogeneity in study design, inconsistent reporting of key methodological details, and limited standardization of interventions.

Future work should focus on rigorously designed clinical studies that incorporate standardized formulations, pharmacokinetic characterization, and integrated outcome assessment encompassing both metabolic and redox domains. Clarifying dose–response relationships, bioavailability constraints, and interactions with established therapies will be critical to defining clinical applicability.

In summary, polyphenols demonstrate a consistent capacity to modulate oxidative stress pathways in T1D, a key contributor to β-cell dysfunction and the development of complications. While glycemic effects remain variable, the consistency and mechanistic depth of their redox-modulating actions position these compounds as compelling adjunctive candidates. By targeting fundamental processes that extend beyond glucose control, polyphenols offer a strategically relevant avenue to enhance current therapeutic paradigms and address critical gaps in the management of disease progression and complication risk.

## Figures and Tables

**Table 1:** Mechanistic and outcome profile of polyphenol classes and subclasses evaluated in preclinical models of type 1 diabetes.

Polyphenol Class/Subclass	Representative Compounds/Interventions	Predominant Reported Mechanisms	Bioavailability/Formulation Considerations	Reported Effects on Glycemic Control	Reported Effects on Oxidative Stress Markers
Flavonols	Quercetin, kaempferol, fisetin, rutin, icariin, morin, troxerutin	Direct ROS scavenging; Nrf2/Keap1 activation; NF-κB suppression; increased SOD, CAT, GPx, and GSH; reduced MDA, ROS, and nitrosative stress; attenuation of inflammatory tissue injury	Several compounds have poor aqueous solubility and variable oral bioavailability; nanoparticle, liposomal, and glycoside formulations may improve systemic exposure	Findings varied across compounds and models; the strongest glycemic effects were reported for quercetin, whereas several other flavonols showed mixed or absent effects on glucose-related outcomes	Antioxidant improvements were frequently reported, including in studies where glycemic improvement was limited or absent
Isoflavones	Genistein, daidzein, puerarin, formononetin, biochanin A	Nrf2 activation; antioxidant enzyme restoration; reduced lipid peroxidation; modulation of β-cell survival pathways; tyrosine kinase inhibition; immune and estrogen-receptor-related signaling	Bioactivity may be influenced by metabolism, sex-specific responses, and formulation; nanoparticle or alternative delivery systems may enhance systemic exposure	Findings were compound- and model-dependent, with more favorable effects reported for puerarin, formononetin, and selected genistein studies	Favorable antioxidant effects were reported in studies assessing oxidative stress, although oxidative endpoints were less frequently evaluated than glycemic outcomes
Flavanols	EGCG, epicatechin, procyanidin B2, pycnogenol, cocoa flavanols	Reduction of ROS and reactive nitrogen species; iNOS suppression; mitochondrial protection; Sirt1-related antioxidant signaling; immune modulation; improved endogenous antioxidant defenses	Stability and absorption may be limited, particularly for EGCG; dietary matrix, extract composition, and combination approaches may influence biological activity	Glycemic improvement was reported in many studies, particularly for EGCG and epicatechin-related interventions, although effects varied by experimental context	Favorable antioxidant effects were reported in studies that measured oxidative stress endpoints, but these endpoints were not uniformly assessed across the subclass
Flavones	Apigenin, luteolin, silymarin, baicalein, nobiletin, diosmin, silybin	Nrf2 activation; NF-κB suppression; reduced MDA, TBARS, protein carbonylation, superoxide, and nitrotyrosine; tissue-specific anti-inflammatory and antioxidant effects	Oral bioavailability may be limited for several flavones; route, dose, duration, and formulation likely influence observed efficacy	Glycemic findings were inconsistent; several compounds improved oxidative or inflammatory injury without clear reductions in glucose-related outcomes	Improvements in tissue oxidative stress and inflammatory–oxidative injury were commonly reported
Flavanones	Naringin, naringenin, hesperetin	ROS reduction; suppression of lipid peroxidation; partial restoration of antioxidant enzyme activity; anti-inflammatory and cardiometabolic effects	Glycoside versus aglycone form may influence absorption and activity; gut metabolism is likely relevant; formulation comparisons were limited	Glycemic effects were mixed, with the clearest signal reported for hesperetin and more limited or variable findings for naringin and naringenin	Antioxidant effects were reported in several studies, but findings were less uniform than those observed for flavonols or flavanols
Stilbenes	Resveratrol and related stilbenoids	Nrf2/ARE activation; Sirt1/SIRT3- and AMPK-related signaling; mitochondrial protection; reduced lipid peroxidation; improved antioxidant enzyme activity; RAGE–NF-κB suppression; anti-inflammatory effects	Resveratrol has low oral bioavailability due to rapid metabolism; formulation strategies may be important for translational application; related stilbenoid analogues may offer improved lipophilicity and exposure	Glycemic effects were frequently favorable but varied according to model, dose, treatment duration, and formulation	Reductions in oxidative damage and restoration of antioxidant defenses were repeatedly reported across studies assessing oxidative stress
Diarylheptanoids	Curcumin and curcuminoid formulations	Nrf2/Keap1/ARE activation; HO-1 and NQO1 upregulation; NF-κB inhibition; cytokine suppression; reduced ROS and lipid peroxidation; β-cell and tissue cytoprotection	Poor aqueous solubility and low systemic bioavailability are major limitations; nanoformulations, adjuvants, derivatives, and alternative delivery systems may improve efficacy	Glycemic findings varied by formulation, dose, route, and model	Antioxidant and anti-inflammatory effects were commonly reported, particularly when optimized formulations or derivatives were used
Natural extracts/multi-polyphenol interventions	Fruit, berry, tea, plant, flavonoid-rich, phenolic-acid-rich, and tannin-rich extracts	Multi-component antioxidant effects; direct ROS scavenging; restoration of endogenous antioxidant defenses; NF-κB suppression; Nrf2 activation in selected extracts; possible synergistic activity among phenolic constituents	Composition varies substantially across extracts; standardization of phenolic content, dose equivalence, and batch reproducibility are major translational limitations	Glycemic findings varied across extract type, composition, dose, and experimental model; attribution to individual compounds was often limited	Favorable oxidative stress findings were commonly reported, although mechanistic attribution was limited by extract complexity
Other polyphenols	Phenolic acids, ellagitannins, tannins, lignans, chalcones, and related compounds	ROS scavenging; reduced lipid peroxidation; antioxidant enzyme modulation; Nrf2/HO-1 activation in selected compounds; tissue-specific cytoprotection	Bioavailability depends strongly on chemical structure, conjugation, and microbial metabolism; formulation was less frequently standardized than for curcumin or resveratrol	Glycemic findings were variable and less well characterized because of the smaller evidence base	Favorable effects on oxidative stress markers were reported in studies that assessed these endpoints

Abbreviations: AMPK, AMP-activated protein kinase; ARE, antioxidant response element; CAT, catalase; EGCG, epigallocatechin gallate; GPx, glutathione peroxidase; GSH, reduced glutathione; HO-1, heme oxygenase-1; iNOS, inducible nitric oxide synthase; MDA, malondialdehyde; NF-κB, nuclear factor kappa B; NQO1, NAD(P)H quinone dehydrogenase 1; Nrf2, nuclear factor erythroid 2-related factor 2; RAGE, receptor for advanced glycation end products; ROS, reactive oxygen species; Sirt1, sirtuin 1; SIRT3, sirtuin 3; SOD, superoxide dismutase; TBARS, thiobarbituric acid reactive substances; T1D, type 1 diabetes.

## Data Availability

No new data were created or analyzed in this study. Data sharing is not applicable to this article.

## Supplementary Materials

The following supporting information can be downloaded at: https://www.mdpi.com/article/10.3390/antiox15060693/s1. Tables S1: A summary of included polyphenol Flavonoid studies; Table S2: A summary of included polyphenol Stilbene studies; Table S3: A summary of included polyphenol Diarylheptanoid studies; Table S4: A summary of included polyphenol Natural Extract studies; Table S5: A summary of included other polyphenol studies. Reference [258] is cited in the Supplementary Material.

## Abbreviations

The following abbreviations are used in this manuscript:

AGEs	Advanced glycation end products
AOPP	Advanced oxidation protein product
ARE	Antioxidant response element
CAT	Catalase
CEC	Chitosan-encapsulated curcumin
CuMP	Curcumin microparticle
DHE	Dihydroethidium
DNA	Deoxyribonucleic acid
EDL	Extensor digitorum longus
EGCG	Epigallocatechin gallate
FRAP	Ferric reducing antioxidant power
GSH	Reduced glutathione
GSSG	Oxidized glutathione
GPx	Glutathione peroxidase
GST	Glutathione S-transferase
HbA1c	Glycated hemoglobin
HO-1	Heme oxygenase-1
HOMA	Homeostatic model assessment
H_2_O_2_	Hydrogen peroxide
IL	Interleukin
IP	Intraperitoneal
IPGTT	Intraperitoneal glucose tolerance test
ITT	Insulin tolerance test
Keap1	Kelch-like ECH-associated protein 1
MDA	Malondialdehyde
MnSOD	Manganese superoxide dismutase
MPO	Myeloperoxidase
NADPH	Nicotinamide adenine dinucleotide phosphate
NF-κB	Nuclear factor kappa B
NOD	Non-obese diabetic
NOX	NADPH oxidase
NQO1	NAD(P)H quinone dehydrogenase 1
Nrf2	Nuclear factor erythroid 2-related factor 2
OGTT	Oral glucose tolerance test
PCO	Protein carbonyl
PI3K	Phosphoinositide 3-kinase
RAGE	Receptor for advanced glycation end products
ROS	Reactive oxygen species
RNS	Reactive nitrogen species
SIRT1	Sirtuin 1
SIRT3	Sirtuin 3
SOD	Superoxide dismutase
STZ	Streptozotocin
TBARS	Thiobarbituric acid reactive substance
T1D	Type 1 diabetes
T2D	Type 2 diabetes
TNF-α	Tumor necrosis factor alpha
