# Supplementary material for: Polyphenol-Mediated Modulation of Oxidative Stress Pathways in Type 1 Diabetes: A Systematic Review
Source: Antioxidants (Basel). 2026 May 30;15(6):693. doi: 10.3390/antiox15060693 (PMC13295557; doi:10.3390/antiox15060693)
Supplement: Supplementary file 1 [file antioxidants-15-00693-s001.zip › Supplementary Table 1.pdf]

Supplementary Table 1: A summary of included polyphenol Flavonoid studies

Reference	Country	T1D Induction Method	Animal Model	Polyphenol Subclass	Polyphenol Investigated	Outcomes Summarized
Abuohashish et al. 2021. [49]	Saudi Arabia	STZ	Wistar Rats	Flavonol	Morin	• Morin 30 mg/kg significantly reduced blood glucose levels ($p<0.05$); significantly restored insulin ($p<0.05$), C-peptide ($p<0.05$), and IGF-1 ($p<0.05$)<br>• Morin 30 mg/kg significantly restored SOD ($p<0.01$), CAT ($p<0.01$), and GST ($p<0.05$); Morin did not significantly restore GPx by 15 mg/kg
Ahmadzadeh et al. 2024. [77]	Iran	STZ	C57BL/6 Mice	Flavanol	Epigallocatechin-3-Gallate (EGCG)	• EGCG significantly reduced blood glucose levels ($p<0.0001$); significantly increased fasting serum insulin levels ($p=0.015$)<br>• No Oxidative Stress outcomes reported
Ahmed et al. 2023. [97]	Egypt	STZ	Wistar Rats	Flavanone	Hesperetin	• Hesperetin significantly decreased fasting blood glucose ($p<0.05$) and 2 h post-glucose loading blood glucose ($p<0.05$); significantly improved glucose tolerance and significantly decreased OGTT AUC ($p<0.05$); significantly decreased serum fructosamine ($p<0.05$); significantly increased serum insulin ($p<0.05$) and serum C-peptide ($p<0.05$)<br>• Hesperetin significantly decreased hepatic MDA/LPO ($p<0.05$); significantly increased hepatic GSH ($p<0.05$); significantly increased hepatic GR ($p<0.05$); significantly increased hepatic GST ($p<0.05$); did not significantly affect hepatic GPx
Altamimi et al. 2021. [46]	Saudi Arabia	STZ	Wistar Rats	Flavonol	Fisetin	• Fisetin significantly reduced fasting plasma glucose($p<0.001$); significantly increased fasting insulin ($p<0.01$); significantly reduced HOMA-IR ($p<0.001$); significantly reduced glucose AUC during OGTT and IPITT ($p<0.001$)<br>• Fisetin significantly decreased LV ROS and MDA ($p<0.001$); significantly increased LV

						GSH and SOD (p<0.001)
Bramora et al. 2021. [99]	Poland	STZ	Wistar Rats	Flavanone	Naringenin	• No Glycemic Control outcomes reported<br>• Naringenin significantly decreased AOPP (p<0.05) in the 50 mg/kg group and MDA in the 50 mg/kg (p<0.05) and 100 mg/kg (p<0.01); did not significantly affect SOD, CAT, GPx, GSH, tGSH, GSSG, TOS or TAR levels;
Chen et al. 2024. [64]	China	Alloxan	C57BL/6 Mice	Isoflavone	Formononetin (FMNT)	• FMNT significantly decreased blood glucose levels at all doses (p<0.05, p<0.01, or p<0.001); significantly increased low insulin-positive cell counts (p<0.001)<br>• FMNT significantly decreased intracellular ROS overproduction (p<0.001); significantly downregulated Keap1 and upregulated Nrf2, HO-1, NQO1 protein expression in pancreatic tissues (p<0.05 to p<0.001)
Chen et al. 2018. [45]	China	STZ	C57BL/6 Mice	Flavonol	Kaempferol	• Kaempferol did not significantly affect fasting blood glucose or body weight (p>0.05)<br>• Kaempferol significantly reduced ROS generation in heart tissue (p<0.001); significantly reduced 3-NT positive area (p<0.01); significantly upregulated Nrf2 and NQO-1 expression (p<0.001)
Chis et al. 2023. [37]	Romania	STZ	Wistar Rats	Flavonol	Quercetin	• Quercetin significantly reduced bloog glucose in both sedentary and trained groups (p<0.0001)<br>• No Oxidative Stress outcomes reported
Choi et al. 2008. [69]	South Korea	NOD	NOD Mice	Isoflavone	Genistein; Daidzein	• Genistein and daidzein significantly reduced fasting blood glucose at week 9 (p<0.05); significantly increased plasma insulin (p<0.05); significantly increased C-peptide (p<0.05)<br>• Genistein and daidzein significantly lowered lipid peroxidation in erythrocytes

						(p<0.05) and liver (p<0.05)
David et al. 2023. [41]	USA, Brunei	STZ	Sprague-Dawley Rats	Flavonol	Rutin	• Rutin significantly decreased blood glucose (p<0.01)<br>• No Oxidative Stress outcomes reported
Dholakia et al. 2024. [67]	India	STZ	Sprague-Dawley Rats	Isoflavone	Genistein	• GEN-loaded nanoparticle DPI (DLCS7) significantly reduced BGLs (p<0.0001)<br>• No Oxidative Stress outcomes reported
Ding et al. 2022. [50]	China	NOD	NOD Mice	Flavonol	Astilbin	• Astilbin significantly controlled blood glucose curves during the observation period (p<0.05)<br>• Astilbin significantly increased cytoplasmic ROS in murine CD4+ T cells after 24 h treatment (p<0.05 to p<0.001); did not significantly change mitochondrial ROS or mitochondrial weight
Dong et al. 2022. [39]	China	STZ	Wistar Rats	Flavonol	Quercetin	• Quercetin had no significant impact on blood glucose<br>• Quercetin increased the fluorescence density of Nrf2; significantly decreased the levels of MDA; significantly increased T-AOC (p<0.05) at medium and high doses.
Du et al. 2017. [40]	China	STZ	Sprague-Dawley Rats	Flavonol	Quercetin	• Quercetin had no significant effect on fasting blood glucose in any dose group<br>• Quercetin significantly increased GSH, GSH/GSSG ratio, T-SOD activity, GSH-Px activity (all p<0.05); significantly reduced MDA content in rat lens tissue (p<0.01); significantly reduced GSSG (p<0.01)
Elmarakby et al. 2011. [68]	USA	STZ	C57BL/6 Mice	Isoflavone	Genisterin	• Genistein significantly reduced blood glucose levels (p<0.05); did not significantly affect plasma insulin levels<br>• Genistein significantly reduced urinary TBARs excretion (p<0.05); significantly reduced renal gp91phox expression (p<0.05)

Eraky et al. 2022. [31]	Egypt	STZ	Sprague-Dawley Rats	Flavonol	Quercetin	• Quercetin significantly decreased blood glucose (p<0.001)<br>• Quercetin significantly decreased MDA levels (p<0.001)
Fan et al. 2021. [83]	China	STZ	C57BL/6 Mice	Flavanol	Procyanidin B2 (PCB2)	• PCB2 treatment significantly reduced serum glucose levels (p<0.05)<br>• PCB2 treatment significantly decreased MDA levels in peripheral blood (p<0.01); significantly reduced superoxide production in dermal wound tissues by DHE staining (p<0.01)
Faulkner et al. 2015. [94]	USA	STZ	C57BL/6 Mice	Flavone	Baicalein	• Baicalein treatment (4 or 10 weeks) did not significantly reduce plasma glucose<br>• Baicalein treatment for 10 weeks significantly reduced TBARs excretion (p<0.05); 4-week baicalein treatment failed to significantly lower TBARS
Fu et al. 2011. [75]	USA	NOD	NOD/LtJ Mice	Flavanol	Epigallocatechin-3-Gallate (EGCG)	• EGCG significantly ameliorated hyperglycemia after 17 weeks (p=0.0001); significantly improved fasting blood glucose and glucose tolerance (p=0.001); significantly reduced HbA1c (p=0.015); EGCG significantly increased plasma insulin levels (p=0.003); diabetes incidence decreased (p=0.013)<br>• No Oxidative Stress outcomes reported
Fu et al. 2013. [79]	USA	NOD	NOD/LtJ Mice	Flavanol	Epicatechin	• Epicatechin significantly increased plasma insulin (p<0.05); significantly decreased HbA1C (p<0.05)<br>• No Oxidative Stress outcomes reported
Gamal Sherif et al. 2024. [87]	Egypt	STZ	Wistar Rats	Flavone	Apigenin	• Apigenin significantly decreased fasting blood glucose (p<0.0001)<br>• No Oxidative Stress outcomes reported
Ganesan et al. 2020. [42]	India	Alloxan	Wistar Rats	Flavonol	Rutin	• Rutin significantly reduced fasting blood glucose in diabetic rats on day 30 (p<0.05); significantly reduced urinary glucose (p<0.05)<br>• No Oxidative Stress outcomes

						reported
Habib et al. 2024. [30]	Egypt	STZ	Sprague-Dawley Rats	Flavonol	Quercetin	• Quercetin significantly decreased fasting blood glucose ($p<0.05$); significantly increased serum insulin ($p<0.05$)<br>• Quercetin significantly reduced kidney MDA ($p<0.05$); significantly increased kidney SOD, GPx, and CAT ($p<0.05$)
Hossein et al. 2024. [35]	Iran	STZ	Wistar Rats	Flavonol	Quercetin	• Quercetin significantly reduced plasma glucose ($p<0.001$); significantly increased plasma insulin ($p<0.001$); significantly reduced urine glucose ($p<0.001$)<br>• Quercetin significantly decreased MDA ($p<0.001$); significantly increased SOD, CAT and GSH (all $p<0.001$), CAT ($p<0.001$), GSH ($p<0.001$);
Hou et al. 2020. [61]	China	STZ	C57BL/6 Mice	Isoflavone	Puerarin	• Puerarin dose-dependently reduced FBG from second week of administration ($p<0.05$); 400 mg/kg significantly reduced PBG from first week ($p<0.05$); significantly restored glucose tolerance ($p<0.05$); significantly reduced fructosamine ($p<0.05$)<br>• Puerarin significantly reduced plasma MDA ($p<0.05$); significantly elevated SOD activity ($p<0.05$); significantly restored GSH levels ($p<0.05$)
Hsu et al. 2017. [91]	Taiwan	STZ	Sprague-Dawley Rats	Flavone	Diosmin	• Diosmin significantly decreased blood glucose at 80 mg/kg ($p<0.05$), 120 mg/kg and 160 mg/kg ($p<0.01$)<br>• No Oxidative Stress outcomes reported
Huang et al. 2017. [66]	USA	NOD	NOD Mice	Isoflavone	Genistein	• Genistein significantly reduced blood glucose level on day 98 after initial dosing ($p<0.05$); significantly improved GTT ($p<0.05$); significantly delayed onset of T1D in NOD females ($p<0.05$); no significant

						change in BGL in females<br>• No Oxidative Stress outcomes reported
Huang et al. 2019. [70]	USA	NOD	NOD Mice	Isoflavone	Daidzein	• Daidzein did not significantly affect blood glucose, GTT or ITT<br>• No Oxidative Stress outcomes reported
Khaled et al. 2024. [86]	Egypt	STZ	Wistar Rats	Flavone	Apigenin	• Apigenin significantly decreased fasting blood glucose (p<0.001)<br>• No Oxidative Stress outcomes reported
Khater et al. 2024. [32]	Egypt	STZ	Sprague-Dawley Rats	Flavonol	Lipid nanoparticles of quercetin (QU-Lip)	• QU-Lip significantly reduced FBG (p<0.0001); significantly increased serum insulin (p<0.0001); significantly increased serum C-peptide (p<0.0001)<br>• QU-Lip significantly decreased pancreatic MDA (p<0.0001); significantly increased pancreatic SOD (p<0.0001); significantly increased pancreatic GPx (p<0.0001); significantly increased pancreatic CAT (p<0.0001)
Khazim et al. 2013. [95]	USA, Israel	Genetic Model	OVE26 mice	Flavone	Silybin	• Silybin did not significantly affect blood glucose levels<br>• Silybin significantly reduced 2-OH-E production in the renal cortex (p<0.05); prevented increased Nox4 expression in kidney cortex of OVE26 mice
Kim et al. 2003. [80]	South Kroea	STZ	Sprague-Dawley Rats	Flavanol	Epicatechin	• Epicatechin significantly lowered blood glucose (p<0.05)<br>• Epicatechin significantly reduced nitrie production in islets (p<0.001); significantly ehnahced nitride production in a dose-dependent manner (p<0.0001)
Kodidela et al. 2020. [78]	India	STZ	Wistar Rats	Flavanol	Epigallocatechin Gallate (EGCG)	• EGCG significantly decreased FBG (p<0.05)<br>• EGCG significantly restored MDA, PCO levels in renal tissue (p<0.05); significantly restored GSH, GPx, GST, SOD, CAT in serum (p<0.05); significantly restored MDA in mitochondria (p<0.05)

Leo et al. 2011. [53]	Australia	STZ	Wistar Rats	Flavonol	DiOHF	• DiOHF significantly decreased the blood glucose and HbA1C levels (p<0.05)<br>• DiOHF attenuated superoxide levels in diabetic arteries (p<0.01)
Li et al. 2014. [62]	China	STZ	C57BL/6 Mice	Isoflavone	Puerarin	• Puerarin significantly reduced blood glucose (p<0.001); significantly improved glucose tolerance (AUC, p<0.001); significantly increased serum insulin (p<0.05)<br>• Puerarin significantly decreased CoCl2-induced ROS generation in MIN6 cells (p<0.05); significantly increased MnSod mRNA expression (p<0.05); significantly increased Gpx1 mRNA expression (p<0.05)
Li et al. 2019. [92]	China	STZ	C57BL/6 Mice	Flavone	Luteolin	• Luteolin did not significantly affect blood glucose levels (p>0.05)<br>• Luteolin significantly reduced DHE-positive staining (p<0.001); significantly reduced 3-NT staining (p<0.01); significantly restored SOD activity (p<0.001), significantly increased Nrf2 protein and mRNA (p<0.05); significantly increased HO-1 mRNA (p<0.001); significantly increased NQO-1 mRNA (p<0.001)
Lin et al. 2017. [81]	Taiwan	STZ	C57BL/6 Mice	Flavanol	(−)-Epicatechin-3-O-β-D-allopyranoside (BB)	• BB (B1, B2, B3) significantly reduced blood glucose (p<0.001) and HbA1C (p<0.05–0.001); B2 and B3 significantly increased serum insulin (p<0.05)<br>• No Oxidative Stress outcomes reported
Luo et al. 2021. [43]	China	STZ	C57BL/6 Mice	Flavonol	Kaempferol	• KPF significantly reduced blood glucose levels (p<0.05)<br>• No Oxidative Stress outcomes reported
Maher et al. 2011. [48]	USA	Genetic Model	Akita Mice	Flavonol	Fisetin	• Fisetin had no significant effect on blood glucose or HbA1c<br>• Fisetin significantly prevented diabetes-induced increase in kidney TBARS (p<0.05); significantly restored diabetes-reduced GCL expression in kidney (p<0.01); significantly reduced elevated

						RAGE expression in kidney (p<0.01)
Malihi et al. 2009. [90]	Iran	STZ	Albino Mice	Flavone	Silymarin	• Silymarin (40 and 80 mg/kg) significantly reduced fed blood glucose at day 14 and day 21 (p<0.01)<br>• Silymarin (40 and 80 mg/kg) significantly reduced TBARS (p<0.01); significantly reduced protein carbonyl content in dose-dependent manner (p<0.05); significantly increased TTM (p<0.01); significantly increased FRAP (p<0.01); significantly reduced MPO activity (p<0.01)
Malik et al. 2017. [88]	India	STZ	Wistar Rats	Flavone	Apigenin	• Apigenin did not significantly affect blood glucose<br>• Apigenin (20 mg/kg/day) significantly reduced kidney MDA (p<0.05); significantly increased kidney GSH (p<0.05); significantly increased kidney SOD (p<0.05), and significantly increased kidney CAT (p<0.01); Apigenin (5 and 10 mg/kg/day) did not significantly affect kidney MDA/GSH/SOD/CAT
Mehrabadi et al. 2018. [65]	Iran, Canada	STZ	Wistar Rats	Isoflavone	Biochanin A (BCA)	• BCA significantly decreased blood glucose for both doses (p<0.05)<br>• No Oxidative Stress outcomes reported
Melo et al. 2024. [84]	Brazil	STZ	Wistar Rats	Flavanol	Cocoa Flavanol (CF)	• CF supplementation significantly potentiated glycemic reduction 60 minutes after aerobic exercise (p < 0.05); CF significantly reduced glucose AUC (p<0.01)<br>• No Oxidative Stress outcomes reported
Miranda et al. 2018. [33]	Brazil	Alloxan	Fischer Rats	Flavonol	Quercetin	• Quercetin formulation significantly reduced fasting blood glucose and significantly increased serum insulin levels (p<0.0001)<br>• No Oxidative Stress outcomes reported

Miranda et al. 2020. [89]	Brazil	Alloxan	Fischer Rats	Flavone	Silymarin	• Silymarin at 50 or 100 mg/kg/day did not significantly decrease serum glucose levels<br>• SMN at both doses significantly reduced hepatic PCG levels ($p<0.0001$, $p=0.0028$); at both doses significantly reduced pancreatic PCG levels ($p=0.0023$, $p=0.0001$); hepatic and pancreatic SOD and CAT activities were not significantly different
Murunga et al. 2016. [100]	South Africa	STZ	Sprague-Dawley Rats	Flavanone	Naringin	• Naringin significantly improved fasting plasma insulin ($p<0.01$); did not significantly improve FBG; did not significantly improve GTT AUC<br>• Naringin significantly ($p<0.0001$) reduced plasma MDA concentrations
Najafi et al. 2018. [52]	Iran	STZ	Wistar Rats	Flavonol	Troxerutin	• Troxerutin did not significantly affect blood glucose<br>• Troxerutin significantly reduced myocardial IL-1β levels ($p<0.05$); significantly reduced myocardial TNF-α levels ($p<0.05$)
Parveen et al. 2013. [82]	India	STZ	Wistar Rats	Flavanol	Pycnogenol (PYC)	• PYC significantly reduced FBG ($p<0.05$); significantly reduced HbA1c ($p<0.05$); significantly increased serum insulin ($p<0.05$); significantly increased amylase activity ($p<0.05$); significantly increased hepatic glycogen ($p<0.05$); significantly improved OGTT glucose tolerance at 60 and 120 min ($p<0.05$)<br>• PYC significantly decreased TBARS and protein carbonyl in liver and pancreas (both $p<0.05$); significantly increased FSH In liver and pancreas ($p<0.05$); significantly increased GST and CAT activity ($p<0.05$)
Stechyshyn et al. 2020. [38]	Ukraine	STZ	Wistar Rats	Flavonol	Quercetin	• Quercetin (water-soluble) significantly decreased serum glucose ($p<0.05$); significantly decreased HbA1c ($p<0.05$); Quercetin (liposomal) significantly decreased serum glucose ($p<0.05$); significantly decreased HbA1c ($p<0.05$)

						• No Oxidative Stress outcomes reported
Posokhova et al. 2018. [36]	Ukraine	STZ	Wistar Rats	Flavonol	Quercetin (Corvitin – water-soluble; Lipoflavon – liposomal)	• Lipoflavon significantly reduced glucose and HbA1c; HbA1c normalized under Lipoflavon treatment (p<0.05)<br>• No Oxidative Stress outcomes reported
Qadiri et al. 2019. [51]	Iran	STZ	Wistar Rats	Flavonol	Troxerutin	• Troxerutin significantly lowered blood glucose (p<0.001)<br>• Troxerutin did not have a significant impact in MDA content, and GPA and SOD activites.
Qiu et al. 2017. [63]	China	Alloxan	Kunming Mice	Isoflavone	Formononetin (FMN)	• FMN significantly reduced FBG in 5, 10, 20 mg/kg groups (p<0.01); significantly reduced OGTT AUC in all dose groups (p<0.01); significantly increased serum insulin activity all dose groups (p<0.01); significantly increased hepatic glycogen in all dose groups (p<0.01)<br>• No Oxidative Stress outcomes reported
Rodríguez et al. 2024. [102]	Argentina	STZ	Wistar Rats	Flavanone	Naringin	• Naringin alone did not significantly affect blood glucose or serum insulin; only concurrent insulin+naringin treatment significantly decreased serum glucose (p<0.05);<br>• No Oxidative Stress outcomes reported
Song et al. 2003. [76]	South Korea	STZ	C57BL/KsJ Mice	Flavanol	Epigallocatechin-3-Gallate (EGCG)	• EGCG treatment significantly reduced hyperglycemia (p not reported)<br>• EGCG markedly suppressed MLD-STZ-induced iNOS mRNA expression in isolated islets (p not reported)
Song et al. 2024. [44]	China	STZ	C57BL/6 Mice	Flavonol	Kaempferol	• KPF 50 mg/kg significantly reduced blood glucose (p<0.01)<br>• KPF treatment significantly reduced ROS levels (p<0.01)

Lei et al. 2015. [58]	China	STZ	Sprague-Dawley Rats	Flavonol	Icariside II (ICA II)	• ICA II did not significantly affect blood glucose or HbA1c<br>• ICA II significantly attenuated MDA levels in kidney tissues of diabetic rats ($p<0.05$)
Tie et al. 2013. [72]	China	STZ	C57BL/6 Mice	Isoflavone	Genistein	• Genistein at 0.2, 1, and 5 mg/kg did not significantly modify blood glucose<br>• Genistein at all doses (0.2, 1, 5 mg/kg) significantly attenuated cutaneous $O_2^{\cdot-}$ production ($p<0.05$); significantly dose-dependently suppressed nitrotyrosine formation ($p<0.05$)
Wang et al. 2013. [34]	China	STZ	Sprague-Dawley Rats	Flavonol	Quercetin	• Quercetin significantly decreased blood glucose in a dose-dependent manner ($p<0.05$)<br>• Quercetin at 50 and 100 mg/kg significantly decreased hepatic $O_2^{\bullet-}$ levels ($p<0.05$); 100 mg/kg quercetin significantly decreased hepatic H_2O_2 levels ($p<0.05$); quercetin significantly reduced intracellular ROS in BRL-3A and HepG2 cells ($p<0.05$)
Wang et al. 2020. [55]	China	STZ	Sprague-Dawley Rats	Flavonol	Icariin	• Icariin did not significantly change blood glucose, fasting insulin level, or insulin sensitivity index in diabetic rats<br>• Icariin significantly decreased MDA content ($p<0.05$); significantly increased SOD activity ($p<0.05$); significantly reduced superoxide anion levels ($p<0.05$); significantly increased HO-1, NQO1, SOD2, Trx1 expression ($p<0.05$); significantly promoted Nrf2 nuclear translocation ($p<0.05$)
Wojnar et al. 2018. [98]	Poland	STZ	Wistar Rats	Flavanone	Naringenin	• Naringenin did not significantly affect serum glucose<br>• Naringenin significantly reduced SOD activity ($p<0.05$); significantly reduced CAT activity at 100 mg/kg/day ($p<0.05$); significantly reduced AOPP at 50 mg/kg/day and 100 mg/kg/day ($p<0.05$); significantly reduced PCG at 100 mg/kg/day ($p<0.05$); significantly reduced MDA at

						50 mg/kg/day and 100 mg/kg/day (p<0.05); did not significantly affect PCG at 50 mg/kg/day
Woodman et al. 2009. [54]	Australia	STZ	Sprague-Dawley Rats	Flavonol	DiOHF	• DiOHF did not significantly affect blood glucose levels<br>• DiOHF reduced superoxide generation in diabetic aortae (p<0.05)
Wu et al. 2017. [74]	China	STZ	Sprague-Dawley Rats	Flavanol	Epigallocatechin-3-Gallate (EGCG)	• EGCG did not significantly alter blood glucose<br>• EGCG significantly reduced cardiac MDA levels (p<0.05); significantly reduced 15-F2t-isoprostane formation (p<0.05); significantly increased MnSOD protein expression (p<0.05)
Wu et al. 2024. [59]	China	STZ	C57BL/6 Mice	Flavonol	Quercetin	• No Glycemic Control outcomes reported<br>• Quercetin significantly increased kidney Nrf2 protein levels (p<0.001); significantly increased kidney HO-1 protein levels (p<0.01); significantly increased kidney SOD1 protein levels (p<0.001)
Xulu et al. 2012. [101]	South Africa	STZ	Wistar Rats	Flavanone	Naringin	• Naringin did not significantly reduce FBG (p>0.05); did not significantly improve glucose intolerance (p>0.05)<br>• No Oxidative Stress outcomes reported
Yang et al. 2018. [71]	China	STZ	Sprague-Dawley Rats	Isoflavone	Genistein	• Genistein did not significantly impact blood glucose<br>• No Oxidative Stress outcomes reported
Yao et al. 2021. [57]	China	STZ	Sprague-Dawley Rats	Flavonol	Icariin	• Icariin did not significantly alter serum glucose<br>• Icariin at 80 mg/kg significantly reduced GPR78 expression in aortas (p<0.001); significantly reduced CHOP expression (p<0.001); significantly increased p-eNOS expression (p<0.001)

Yao et al. 2024. [56]	China	STZ	Sprague-Dawley Rats	Flavonol	Icariin	• Icariin did not significantly alter serum glucose<br>• Icariin significantly increased p-eNOS expression in aortas (p<0.001); significantly increased GPER expression (p<0.01); significantly increased Sirt1 expression (p<0.05)
Zavodnik et al. 2025. [103]	Belarus	STZ	Wistar Rats	Flavanone	Naringin	• Naringin significantly reduced glycated haemoglobin (HbA1c) (p<0.05); did not significantly reduce blood glucose or insulin levels or change HOMA-IR<br>• Naringin significantly reduced kidney tissue TBARS (p<0.05); significantly reduced erythrocyte TBARS (p<0.05; did not significantly affect kidney GSH; did not significantly restore erythrocyte GSH concentration; did not significantly impact erythrocyte glutathione peroxidase activity
Zhang et al. 2016. [93]	China	STZ	C57BL/6 Mice	Flavone	Nobiletin	• Nobiletin treatment did not significantly affect blood glucose levels<br>• Nobiletin significantly reduced cardiac MDA levels (p<0.05); significantly increased SOD1 activity and SOD1 protein expression (both p<0.05)
Zhao et al. 2015. [47]	China	STZ	C57BL/6 Mice	Flavonol	Fisetin	• Fisetin did not significantly affect blood glucose levels<br>• Fisetin significantly reduced MDA levels (p not reported); significantly reduced ROS production (p not reported); significantly increased catalase activity (p not reported) in sciatic nerve, DRG, and spinal cord
