# Supplementary material for: Polyphenol-Mediated Modulation of Oxidative Stress Pathways in Type 1 Diabetes: A Systematic Review
Source: Antioxidants (Basel). 2026 May 30;15(6):693. doi: 10.3390/antiox15060693 (PMC13295557; doi:10.3390/antiox15060693)
Supplement: Supplementary file 1 [file antioxidants-15-00693-s001.zip › Supplementary Table 2.pdf]

Supplementary Table 2: A summary of included polyphenol Stilbene studies

Reference	Country	T1D Induction Method	Animal Model	Polyphenol Subclass	Polyphenol Investigated	Outcomes Summarized
Abdel-Bakky et al. 2022. [115]	Saudi Arabia, Egypt	STZ	BALB/c Mice	Stilbene	Resveratrol	• Resveratrol significantly decreased blood glucose and plasma insulin (p<0.05)<br>• Resveratrol significantly decreased serum levels of NO (p<0.05)
Abdelali et al. 2016. [109]	Kuwait	STZ	Wistar Rats	Stilbene	Trans-Resveratrol	• No Glycemic Control outcomes reported<br>• Trans-Resveratrol significantly increased TAS/TOS ratio (p<0.05), significantly decreased 8-oxo-dG levels in testis and spermatozoa (p<0.05); significantly decreased DNA fragmentation (p<0.05)
Akar et al. 2011. [132]	Turkey	Alloxan	New Zealand Rabbits	Stilbene	Trans-Resveratrol	• Trans-Resveratrol significantly increased serum insulin in 50 mg/L groups (p<0.05); did not significantly reduce serum glucose<br>• Trans-Resveratrol significantly reduced lipid hydroperoxide (p<0.05); significantly suppressed basal and NAD(P)H-stimulated superoxide production (p<0.05); significantly increased aortic and serum nitrite/nitrate levels (p<0.05)
Al-Hussaini et al. 2021. [131]	Kuwait	STZ	Agouti Rats	Stilbene	Trans-Resveratrol	• Trans-resveratrol reduced blood glucose in diabetic rats only at week 1 (p<0.05)<br>• Trans-resveratrol supplementation resulted in significant recovery of 4-HNE (p<0.05)
Bagul et al. 2015. [111]	India	STZ	Sprague-Dawley Rats	Stilbene	Resveratrol	• Resveratrol significantly decreased blood glucose (p<0.01) and increased serum insulin (p<0.05)<br>• Resveratrol significantly increased GSH (p<0.01), Glutathione Peroxidase, Glutathione Reductase, Glutathione S-

						transferase (p<0.05) and decreased Protein Carbonyl Content (p<0.01) and Advanced Glycation End Products (p<0.05)
Bagul et al. 2018. [119]	India	STZ	Sprague-Dawley Rats	Stilbene	Resveratrol	• Resveratrol significantly reduced blood glucose (p<0.01); significantly reduced HbA1c (p<0.05); significantly increased plasma insulin (p<0.05)<br>• Resveratrol significantly reduced cardiac ROS p<0.01); significantly reduced cardiac TBARS (p<0.01); significantly increased SOD activity (p<0.05); significantly increased catalase activity (p<0.01); significantly increased GSH p<0.05) in heart
Bresciani et al. 2014. [128]	Italy	STZ	Sprague-Dawley Rats	Stilbene	Trans-Resveratrol	• Trans-Resveratrol significantly reduced blood glucose at 5 mg/kg/day after the third week of treatment (p<0.05)<br>• No Oxidative Stress outcomes reported
Carolo dos Santos et al. 2014. [110]	Brazil	STZ	Wistar Rats	Stilbene	Resveratrol	• Resveratrol significantly decreased glycemia (p<0.05)<br>• Resveratrol significantly decreased cardiac lipid hydroperoxide and protein carbonyl (p<0.05); significantly increased cardiac GSH and GR (p<0.05)
Chang et al. 2014. [113]	Taiwan	STZ	Long-Evans Rats	Stilbene	Resveratrol (RSV)	• RSV at all doses significantly reduced hyperglycemia and improved hypoinsulinemia (both p<0.05)<br>• RSV significantly reduced superoxide anion content in EDL muscle (p<0.05) and SOL muscle (p<0.05); significantly downregulated CuZnSOD protein in EDL muscle (p<0.05); significantly upregulated

						MnSOD protein in SOL muscle (p<0.05)
Darwish et al. 2021. [117]	Egypt	STZ	BALB/c Mice	Stilbene	Resveratrol (RES)	• RES significantly reduced fasting blood glucose (p<0.05); significantly reduced random blood glucose; significantly increased fasting serum insulin (p<0.05); significantly attenuated glucose intolerance (p<0.05)<br>• RES significantly reduced serum MDA (p<0.05); significantly increased blood GSH (p<0.05)
Darwish et al. 2021. [116]	Egypt	STZ	BALB/c Mice	Stilbene	Resveratrol (RES)	• RES significantly reduced fasting blood glucose and significantly increased serum insulin levels (p<0.05)<br>• RES significantly reduced serum MDA (p<0.05) and significantly increased blood GSH (p<0.05)
Delucchi et al. 2012. [133]	Italy	STZ	Wistar Rats	Stilbene	Resveratrol	• Resveratrol did not significantly impact blood glucose<br>• No Oxidative Stress outcomes reported
Kaur et al. 2016. [126]	India	STZ	Sprague-Dawley Rats	Stilbene	Resveratrol	• Resveratrol significantly decreased blood glucose after 4 weeks and significantly improved insulin (both p<0.05); HbA1c was not significantly decreased<br>• Resveratrol significantly decreased pancreatic TBARS (p<0.05); significantly increased pancreatic SOD and GSH (both p<0.05); significantly decreased hepatic TBARS (p<0.05); significantly increased hepatic SOD and CAT (p<0.001, p<0.05); significantly (p<0.05); hepatic GSH not significantly increased

Kolling et al. 2019. [137]	Brazil	STZ	Wistar Rats	Stilbene	Resveratrol	• Neither resveratrol free form nor resveratrol-HPβCD significantly affected hyperglycemia<br>• No Oxidative Stress outcomes reported
Ku et al. 2012. [118]	South Korea	STZ	Sprague-Dawley Rats	Stilbene	Resveratrol	• RSV pretreatment prevented STZ-induced diabetes<br>• No Oxidative Stress outcomes reported
Li et al. 2018. [127]	China	STZ	Kunming mice	Stilbene	Resveratrol	• Resveratrol 10 mg/kg significantly reduced fasting plasma glucose ($p<0.001$); significantly increased FPI and HOMA-β ($p<0.001$); 0.1 and 1 mg/kg did not significantly affect fasting plasma glucose<br>• Resveratrol 10 mg/kg significantly reduced MDA ($p<0.05$); significantly increased T-AOC ($p<0.01$); 0.1 and 1 mg/kg had no significant effect on MDA or T-AOC
Millán et al. 2020. [123]	Spain	Alloxan	New Zealand Rabbits	Stilbene	Pterostilbene	• Pterostilbene significantly reduced blood glucose levels ($p<0.05$)<br>• Pterostilbene significantly restored CAT, GPx ($p<0.05$), and SOD activity (all $p<0.05$); significantly improved GSH/GSSG ratio ($p<0.001$); significantly reduced retinal protein carbonylation ($p<0.01$), 3NO2-Tyr/p-Tyr, m-Tyr/Phe, 3Cl-Tyr/p-Tyr ratios ($p<0.05$); significantly reduced 4-HNE levels ($p<0.05$); significantly reduced H_2O_2 levels ($p<0.05$)
Phyu et al. 2016. [139]	Australia	STZ	Wistar Rats	Stilbene	Resveratrol	• Resveratrol had no significant effect on plasma glucose concentration<br>• No Oxidative Stress outcomes reported

Simas et al. 2017. [112]	Brazil	STZ	Wistar Rats	Stilbene	Resveratrol	• Resveratrol significantly decreased blood glucose ($p<0.05$)<br>• Resveratrol treatment significantly reduced testicular MDA and testicular nitrite concentration ($p<0.05$)
Simas et al. 2021. [114]	Brazil	STZ	Wistar Rats	Stilbene	Resveratrol	• Resveratrol significantly lowered blood glucose ($p<0.05$)<br>• Resveratrol treatment significantly reduced MDA in all tissues ($p<0.01$)
Sulaiman et al. 2010. [135]	USA	STZ	CD1 Mice	Stilbene	Resveratrol (RSV)	• RSV treatment had no significant effect on blood glucose over 12-week period; did not significantly improve serum insulin<br>• RSV treatment for 3 months blocked decline in SIRT1 activity and stimulated it ($p<0.05$)
Tastekin et al. 2023. [124]	Turkey	STZ	Wistar Rats	Stilbene	Pterostilbene (PTS); Resveratrol (RSV)	• All treatment groups significantly reduced final blood glucose ($p<0.05$); PTS group showed significantly greater decrease in glucose than RSV group ($p<0.001$); all treatments significantly increased serum insulin; combining PMF with RSV was significantly more effective in improving insulin levels than RSV alone ($p<0.001$)<br>• All treatment groups significantly reduced TNF-α levels in soleus and EDL muscles ($p<0.05$); RSV was significantly more effective than PTS in reducing TNF-α and NF-κB in both muscle types ($p<0.05$); PMF+RSV showed the most prominent decrease in TNF-α in both muscle types ($p<0.001$)

Wang et al. 2018. [134]	China	STZ	FVB Mice	Stilbene	Resveratrol	• Resveratrol did not significantly affect blood glucose levels at any timepoint ($p>0.05$)<br>• Resveratrol significantly increased Nrf2 protein expression, HO-1, NQO1, SOD1, SOD2, and MT expression at 3 and 6 months ($p<0.05$); significantly reduced 3-NT accumulation at 3 and 6 months ($p<0.05$); significantly reduced 4-HNE accumulation at 3 and 6 months ($p<0.05$)
Wang et al. 2019. [122]	China	NOD	NOD Mice	Stilbene	Resveratrol (RSV)	• RSV alone significantly reduced blood glucose ($p<0.05$); RSV+MSC group maintained normal blood glucose from day 14 through end of experiment ($p<0.05$); C-peptide in MSC+RSV group significantly increased ($p=0.041$)<br>• RSV significantly reduced NF-κB expression in sciatic nerve ($p=0.015$)
Xian et al. 2019. [120]	China	NOD	NOD Mice	Stilbene	Resveratrol	• Resveratrol significantly decreased blood glucose ($p<0.05$)<br>• Resveratrol significantly decreased RAGE expression ($p<0.05$); significantly decreased NF-κB (p65) expression ($p<0.05$); significantly decreased MCP-1 expression ($p<0.05$)
Xian et al. 2020. [121]	China	NOD	NOD Mice	Stilbene	Resveratrol	• Resveratrol significantly reduced blood glucose within 28 days ($p<0.05$); significantly increased C-peptide at 8 weeks ($p<0.05$)<br>• Resveratrol significantly reduced RAGE expression ($p<0.05$); significantly reduced NF-κB (P65) expression ($p<0.05$); significantly reduced NOX4 expression ($p<0.05$)

Xu et al. 2022. [138]	China	STZ	Sprague-Dawley Rats	Stilbene	Resveratrol	• Resveratrol did not significantly affect blood glucose<br>• No Oxidative Stress outcomes reported
Yonamine et al. 2016. [129]	Brazil	STZ	Wistar Rats	Stilbene	Resveratrol	• Resveratrol plus insulin significantly reduced glycosuria vs. insulin alone (p<0.001); resveratrol plus insulin restored fructosamine to non-diabetic levels (p<0.001); blood glucose did not significantly differ between insulin and insulin plus resveratrol groups<br>• No Oxidative Stress outcomes reported
You et al. 2018. [136]	China	STZ	C57BL/6 Mice	Stilbene	Aza resveratrol-chalcone derivative 6b	• 6b did not significantly affect fasting blood glucose or serum insulin<br>• 6b significantly reduced DHE-positive staining (p<0.01, p<0.001), significantly reduced 3-NT-positive staining (p<0.01, p<0.001), significantly increased Nrf2 expression (p<0.01, p<0.001), significantly increased HO-1 expression (p<0.01), and significantly increased NQO-1 expression (p<0.01)
Zhang et al. 2019. [130]	China	STZ	CD1 Mice	Stilbene	Aza resveratrol-chalcone derivative 6b	• Resveratrol did not significantly reduce blood glucose at week 8; resveratrol significantly reduced blood glucose at week 12 (p<0.01)<br>• Resveratrol significantly reduced MDA content and increased Mn-SOD activity in renal cortex (both p<0.01)
Zhao et al. 2020. [125]	China	STZ	C57BL/6 Mice	Stilbene	Piceatannol	• Piceatannol significantly reduced fasting blood glucose (p<0.05); significantly restored serum insulin levels (p<0.01); significantly improved OGTT glucose levels at all time points and reduced AUC (p<0.001)

						• Piceatannol significantly reduced MDA levels ($p<0.05$); significantly increased SOD activity ($p<0.05$); significantly increased GSH-PX activity ($p<0.05$)
