# Supplementary material for: Polyphenol-Mediated Modulation of Oxidative Stress Pathways in Type 1 Diabetes: A Systematic Review
Source: Antioxidants (Basel). 2026 May 30;15(6):693. doi: 10.3390/antiox15060693 (PMC13295557; doi:10.3390/antiox15060693)
Supplement: Supplementary file 1 [file antioxidants-15-00693-s001.zip › Supplementary Table 3.pdf]

Supplementary Table 3: A summary of included polyphenol Diarylheptanoid studies

Reference	Country	T1D Induction Method	Animal Model	Polyphenol Subclass	Polyphenol Investigated	Outcomes Summarized
Abdel Aziz et al. 2012. [164]	Egypt	STZ	Curl: HEL1 Rats	Curcumin Derivative	Novel Curcumin Derivative (NCD)	• NCD significantly lowered blood glucose (p<0.05); significantly increased plasma insulin (p<0.001)<br>• NCD significantly lowered MDA levels (p<0.05) and increased HO-1 expression level (p<0.001) in the pancreas, liver and aorta.
Abdel Aziz et al. 2013. [165]	Egypt	STZ	Curl: HEL1 Rats	Curcumin Derivative	Novel Curcumin Derivative (NCD)	• NCD significantly decreased fasting plasma glucose after 40 days and 2 months (p<0.001); significantly increased plasma insulin after 40 days and 2 months (p<0.001); plasma C-peptide levels corresponded to insulin changes (p<0.001)<br>• No Oxidative Stress outcomes reported
Altamimi et al. 2021. [149]	Saudi Arabia	STZ	Sprague-Dawley Rats	Curcumin	Curcumin	• Curcumin did not significantly reduce plasma glucose (p>0.05) or restore plasma insulin (p>0.05)<br>• Curcumin significantly reduced renal ROS (p<0.05) and MDA (p<0.05); significantly increased renal GSH (p<0.05), MnSOD protein and mRNA (p<0.05), and nuclear Nrf2 levels (p<0.05)
Anchi et al. 2019. [150]	India	STZ	Sprague-Dawley Rats	Curcumin	Curcumin, CuMPs (cur microparticles)	• Curcumin and CuMPs via IP injection significantly lowered blood glucose (p<0.05, p<0.001 respectively)<br>• IP, SC and PO treatment of Curcumin treatment of CuMPs significantly decreased MDA (p<0.001, p<0.001, p<0.05 respectively; Curcumin significantly decreased NO (p<0.05); CuMPs significantly decreased NO (p<0.001); CuMPs significantly maintained tissue stores of GSH (p < 0.001)
Asadi et al. 2019. [152]	Iran	STZ	Wistar Rats	Curcumin	Curcumin	• Curcumin 150 mg/kg/day significantly reduced fasting blood glucose (p<0.001);<br>• Curcumin 150 mg/kg/day significantly restored TAC and TOS (p<0.001), reduced MDA (p=0.012), increased SOD activity (p=0.003), increased GPx activity (p<0.001), and restored catalase activity (p=0.011) in kidney tissue
Aziz et al. 2013. [160]	Egypt	STZ	Wistar Rats	Curcumin, Curcumin Derivative	Curcumin, Novel Curcumin Derivative (NCD)	• Curcumin significantly decreased plasma glucose (p<0.001); significantly increased plasma insulin (p<0.05); significantly decreased glycated hemoglobin (p<0.01)<br>• Curcumin significantly increased HO-1 gene expression and HO activity in cardiac and pancreatic tissue (p<0.001); curcumin-induced HO-1 upregulation was significantly lower than NCD group (p<0.001)

Boarescu et al. 2021. [162]	Romani	STZ	Wistar Rats	Curcumin	Curcumin	• Curcumin significantly lowered blood glucose (p<0.0022); significantly increased C-peptide (p<0.0298)<br>• No Oxidative Stress outcomes reported
Daugherty et al. 2018. [166]	USA	STZ	Swiss Webster Mice	Curcumin Derivative	Curcumin Derivative J147	• J147 significantly decreased blood glucose (p<0.05); significantly decreased HbA1c (p<0.05); did not significantly alter plasma insulin<br>• No Oxidative Stress outcomes reported
Ganugula et al. 2017. [159]	USA	STZ	Sprague-Dawley Rats	Curcumin	Curcumin (nano-formulated, nCUR); plain curcumin (CUR)	• nCUR 10 and 50 mg/kg significantly reduced blood glucose (p<0.05); plain CUR 50 mg/kg did not significantly reduce blood glucose; nCUR 50 mg/kg significantly increased plasma insulin (p<0.05)<br>• nCUR pretreatment significantly reduced 8-oxo-dG immunofluorescence intensity in pancreatic tissue (p<0.05); plain CUR had little to no effect on 8-oxo-dG
Gbr et al. 2021. [144]	Egypt	STZ	Sprague-Dawley Rats	Curcumin	Curcumin	• Curcumin significantly decreased blood glucose level (p<0.05)<br>• Curcumin significantly decreased myocardial MDA (p<0.05); significantly increased myocardial GSH and TAC (p<0.05)
Ghasemi et al. 2019. [156]	Iran	STZ	Wistar Rats	Curcumin	Curcumin	• Curcumin 80 and 130 mg/kg both significantly decreased fasting blood sugar (p<0.05)<br>• Curcumin 130 mg/kg significantly increased total antioxidant capacity (TAC) (p=0.038) and total thiol groups (TTG) (p=0.042); significantly decreased MDA (p=0.04), total oxidant status (TOS) (p=0.035), and NO (p<0.05); 80 mg/kg did not significantly improve TAC, TTG, MDA, or TOS;
Jafari Khataylou et al. 2020. [155]	Iran	STZ	C57BL/6 Mice	Curcumin	Curcumin	• Curcumin significantly decreased fasting blood sugar (p<0.05); significantly increased plasma insulin (p<0.05); significantly increased plasma C-peptide<br>• No Oxidative Stress outcomes reported
Jain et al. 2009. [148]	USA	STZ	Sprague-Dawley Rats	Curcumin	Curcumin	• Curcumin significantly reduced fasting blood glucose (p=0.047); significantly reduced glycated hemoglobin (p<0.04); curcumin did not significantly affect plasma insulin levels<br>• Curcumin significantly reduced plasma protein carbonyl levels (p<0.05); did not significantly reduce red cell lipid peroxidation

Jin et al. 2013. [153]	China	STZ	Sprague-Dawley Rats	Curcumin	Curcumin	• Curcumin did not significantly reduce blood glucose<br>• Curcumin significantly reduced gastric MDA (p=0.004); significantly increased gastric SOD activity (p=0.001)
Keshk et al. 2020. [145]	Egypt	STZ	Wistar Rats	Curcumin	Curcumin	• Curcumin significantly reduced blood glucose levels (p<0.05)<br>• Curcumin significantly reduced MDA (p<0.05); significantly increased TAC (p<0.05); significantly decreased protein carbonyl (p<0.05);
Ono et al. 2015. [157]	Japan	STZ	C57BL/6 Mice	Curcumin	Curcumin	• Curcumin did not significantly affect plasma glucose or insulin concentrations<br>• Curcumin significantly decreased superoxide production (p<0.05); significantly decreased TBARS (p<0.05)
Ravikumar et al. 2020. [151]	India	Alloxan	BALB/c Mice	Curcumin	Curcumin	• Curcumin 200 mg/kg significantly decreased random blood glucose (p<0.01); 100 mg/kg did not significantly decrease blood glucose<br>• No Oxidative Stress outcomes reported
Soetikno et al. 2012. [147]	Japan	STZ	Sprague-Dawley Rats	Curcumin	Curcumin	• Curcumin significantly reduced plasma glucose (p<0.01)<br>• Curcumin significantly reduced cardiac MDA (p<0.01); significantly increased left ventricle GPx activity p<0.05)
Soetikno et al. 2011. [146]	Japan	STZ	Sprague-Dawley Rats	Curcumin	Curcumin	• Curcumin significantly reduced plasma glucose (p<0.05)<br>• Curcumin significantly reduced MDA (p<0.05); significantly significantly increased GPx (p<0.05)
Stojchevski et al. 2024. [167]	USA	STZ	Wistar Rats	Curcumin Derivative	Curcumin analogs (C66; B2BrBC)	• acute C66 pretreatment significantly increased Ins1 mRNA and protein (p<0.05); chronic C66 of B2BrBC did not reverse STZ-induced decreases in Ins1 or Glut2 expression<br>• B2BrBC significantly reduced MDA in plasma (p<0.05), liver (p<0.001), and kidney (p<0.01); B2BrBC significantly reduced AOPP in plasma (p<0.01); B2BrBC significantly increased SOD in plasma (p<0.05), CAT in liver (p<0.01) and kidney (p<0.05), GPx in kidney (p<0.05); C66 significantly reduced MDA in liver (p<0.01) and AOPP in liver (p<0.01) and plasma (p<0.01); C66 significantly increased SOD in plasma (p<0.01) and GPx in liver and kidney (p<0.01); B2BrBC significantly reduced MDA in plasma (p<0.05), AOPP in liver (p<0.05) and kidney (p<0.01), and normalized GPx in kidney (p<0.01); acute C66 did not significantly alter oxidative stress markers.

Sudirman et al. 2019. [158]	Taiwan	STZ	C57BL/6 Mice	Curcumin	Curcumin (Cur); Chitosan-encapsulated curcumin (CEC)	• CEC significantly decreased fasting blood glucose(p<0.05); Cur showed decreased glucose but not significantly; CEC significantly increased serum insulin(p<0.05); Cur increased insulin but not significantly<br>• No Oxidative Stress outcomes reported
Wang et al. 2022. [168]	China	STZ	C57BL/6 Mice	Curcumin Derivative	JM-2 (curcumin analog)	• JM-2 did not significantly change fasting blood glucose or body weight<br>• JM-2 significantly decreased superoxide production (p<0.05); significantly decreased TBARS (p<0.05)
Xie et al. 2017. [154]	China	STZ	Sprague-Dawley Rats	Curcumin	Curcumin	• Curcumin significantly reduced blood glucose (p<0.001)<br>• Curcumin significantly reduced MDA (p<0.01); significantly increased SOD (p<0.001); significantly increased liver GPx (p<0.001) and plasma GPx (p<0.01); curcumin significantly increased liver GSH (p<0.001) and plasma GSH (p<0.001)
Xie et al. 2018. [161]	China	STZ	Sprague-Dawley Rats	Curcumin	Curcumin	• Curcumin significantly reduced blood glucose from day 12 onward (p<0.001); significantly increased plasma insulin (p<0.05); significantly reduced plasma glucagon (p<0.05)<br>• Curcumin significantly reduced plasma levels of MDA (p<0.01); significantly increased SOD (p<0.01); significantly decreased GSH-Px and CAT (p<0.01 and p<0.05 respectively); Curcumin significantly upregulated liver gene expression of CAT, GSH-Px, HO-1, NQO-1 (p<0.05); downregulated SOD1, decreased Nrf2; significantly upregulated Keap1 protein expression (p<0.001)
