# Supplementary material for: Polyphenol-Mediated Modulation of Oxidative Stress Pathways in Type 1 Diabetes: A Systematic Review
Source: Antioxidants (Basel). 2026 May 30;15(6):693. doi: 10.3390/antiox15060693 (PMC13295557; doi:10.3390/antiox15060693)
Supplement: Supplementary file 1 [file antioxidants-15-00693-s001.zip › Supplementary Table 4.pdf]

Supplementary Table 4: A summary of included polyphenol Natural Extract studies

Reference	Country	T1D Induction Method	Animal Model	Polyphenol Subclass	Polyphenol Investigated	Outcomes Summarized
Ab Rahman et al. 2020. [185]	Malaysia	STZ	Sprague-Dawley Rats	Flavonoid-Rich Plant Extract	Synacinn™	• Synacinn™ significantly improved fasting blood glucose ($p<0.05$)<br>• No Oxidative Stress outcomes reported
Abd El-Ghffar et al. 2019. [209]	Saudi Arabia, Egypt, Germany	Alloxan	Wistar Rats	Phenolic Acid & Tannin-Rich Extract	Beta vulgaris Leaves Extract	• B. vulgaris significantly decreased serum glucose ($p < .05$ - 0.001)<br>• Beta vulgaris extract significantly reduced hepatic MDA ($p<0.001–0.05$) and significantly increased hepatic TAO and GSH ($p<0.001–0.05$); significantly reduced TNF-α, IL-1β, IL-6 and significantly downregulated NF-κB ($p<0.001–0.05$)
Taha et al. 2019. [178]	India	STZ	Wistar Rats	Flavonoid-Rich Plant Extract	Simarouba glauca leaves extract	• Simarouba glauca significantly decreased blood glucose and HBA1C ($p<0.001$)<br>• Simarouba glauca significantly reduced catalase activity at 250 mg/kg and 500 mg/kg ($p<0.001$); significantly reduced nitric oxide levels at both doses ($p<0.001$)
Abhishek et al. 2019. [184]	India	Alloxan	Wistar Rats	Flavonoid-Rich Plant Extract	Methanol extract of Psychotria dalzellii (MEPD)	• MEPD significantly decreased blood glucose levels ($p<0.05$)<br>• MEPD significantly reduced hepatic MDA/TBARS levels ($p<0.05$); significantly increased hepatic GSH levels ($p<0.05$)
Abo-Saif et al. 2025. [204]	Egypt	STZ	Wistar Rats	Fruit & Berry Polyphenol Extract	Cranberry Extract	• Cranberry extract did not significantly affect blood glucose<br>• Cranberry extract significantly decreased MDA

						(p<0.005) and caspase 1 levels (p<0.001); significantly increased SOD and GSH (p<0.005)
Ajiboye et al. 2024. [192]	Nigeria	STZ	Wistar Rats	Flavonoid-Rich Plant Extract	Ocimum gratissimum leaf flavonoid-rich extract (OGFL)	• No Glycemic Control outcomes reported<br>• OGFL significantly attenuated MDA, GSH, GST, CAT, GPx, and SOD (p<0.05)
Al-Sultany et al. 2018. [221]	Iraq	Alloxan	Rattus Rattus	Other Plant Extract	Mixed plant phenolics (Cuscuta chinesis Lam.)	• Treatment significantly decreased fasting blood glucose (p<0.05)<br>• No Oxidative Stress outcomes reported
Al-Sultany et al. 2019. [220]	Iraq	Alloxan	Rattus Rattus	Other Plant Extract	Mixed plant phenolics (Cuscuta chinesis Lam.)	• Treatment significantly decreased fasting blood glucose at 30, 60 and 90 days (p<0.05)<br>• No Oxidative Stress outcomes reported
Alanazi et al. 2021. [207]	Saudi Arabia	STZ	Wistar Rats	Phenolic Acid & Tannin-Rich Extract	Loranthus regularis	• L. regularis significantly lowered blood gluose (p<0.01 and p<0.001 in 150 and 300 mg/kg/day groups respectively); significantly increased insulin (p<0.01)<br>• L. regularis significantly decreased TBARS (p<0.01); significantly increased GSH (p<0.05); significantly increased SOD, CAT, and GR levels (p<0.05 and p<0.01 for 150 or 300 mg/lg/day groups respectively); significantly increased GPx and GST enzymatic activity (p<0.05 and p<0.01 respectively)
Alblihd et al. 2023. [225]	Saudi Arabia, Egypt	STZ	Wistar Rats	Other Plant Extract	Okra (Abelmoschus esculentus (L.) Moench) pod extract	• Okra significantly lowered blood glucose (p<0.05) and HbA1C (p<0.001)<br>• No Oxidative Stress outcomes reported

Cabral et al. 2025. [198]	Brazil	STZ	Wistar Rats	Fruit & Berry Polyphenol Extract	Passion fruit peel aqueous extract (PFAE), Passion fruit peel hydroethanolic extract (PFHE)	• PFAE and PFHE significantly decreased serum glucose (p<0.05)<br>• No Oxidative Stress outcomes reported
Bastos et al. 2023. [218]	Brazil	STZ	Wistar Rats	Phenolic Acid & Tannin-Rich Extract	Eugenia sonderiana hydroethanolic leaf extract	• Extract significantly reduced fasting glucose (p<0.05); significantly reduced fructosamine (p<0.05); significantly reduced HbA1c p<0.05); no significant reduction in AGEs<br>• Extract significantly reduced liver and kidney MDA (p<0.05); significantly increased SOD activity in liver and kidney (p<0.05); significantly increased GPx activity in liver and kidney (p<0.05); significantly increased CAT activity in liver and kidney (p<0.05)
Bhadada et al. 2016. [179]	India	STZ	Sprague-Dawley Rats	Flavonoid-Rich Plant Extract	Alcoholic extract of Tephrosia purpurea (AcTp) and flavonoid-rich fraction of T. purpurea (FFTp)	• AcTp and FFTp significantly decreased serum glucose and significantly increased serum insulin (p<0.05)<br>• AcTp and FFTp significantly prevented reduction in lens SOD (p<0.05); significantly prevented depletion of lens GSH (p<0.05); significantly reduced lens MDA/lipid peroxidation (p<0.05)
Bikri et al. 2022. [216]	Morocco	STZ	Wistar Rats	Phenolic Acid & Tannin-Rich Extract	Phenolic fraction concentrate (PFC) from date seeds	• PFC significantly decreased fasting blood glucose (p<0.001)<br>• PFC, with insulin, significantly enhanced CAT and SOD activities in brain regions and peripheral organs (p<0.01); significantly reduced MDA and NO in brain regions and peripheral organs (p<0.01); significantly regulated

						MDA (except hippocampus) and NO (except striatum) ($p<0.05$); CAT activity in all tissues except hippocampus and striatum significantly increased ($p<0.001$)
Bommineni et al. 2022. [229]	India	Alloxan	Albino Rabbits	Other Plant Extract	Moringa oleifera leaves	• Moringa oleifera leaves significantly reduced blood glucose at all doses ($p<0.05$)<br>• No Oxidative Stress outcomes reported
Chaudhry et al. 2016. [208]	Pakistan, Germany	Alloxan	Sprague-Dawley Rats	Phenolic Acid & Tannin-Rich Extract	Heliotropium strigosum extract (Hs. Cr)	• H. strigosum significantly decreased blood glucose in a dose-dependent manner ($p<0.001$)<br>• No Oxidative Stress outcomes reported
Chelbi et al. 2025. [212]	Algeria	STZ	Wistar Rats	Phenolic Acid & Tannin-Rich Extract	Methanolic extract of Algerian Trigonella foenum-graecum L. seeds (MEF)	• MEF significantly decreased fasting blood glucose ($p<0.001$)<br>• No Oxidative Stress outcomes reported
Cui et al. 2016. [191]	China	STZ	NIH Swiss Outbred Mice	Flavonoid-Rich Plant Extract	Astragalus polysaccharides (APS), Crataegus flavonoids (CF)	• AC treatment significantly reduced fasting blood glucose at weeks 3 and 4 ($p<0.05$ and $p<0.01$); CF alone did not significantly reduce FBG after 2 weeks; AC and CF significantly increased serum insulin ($p<0.01$); AC and CF significantly reduced OGTT AUC ($p<0.05$)<br>• No Oxidative Stress outcomes reported
De Lima et al. 2018. [224]	Brazil	STZ	Swiss Mice	Other Plant Extract	Ilex paraguariensis (yerba mate) aqueous extract	• IP extract significantly reduced blood glucose and serum fructosamine ($p<0.05$)<br>• IP extract significantly reduced TBARS in liver, kidney, and brain (all $p<0.05$); significantly reduced SOD activity in liver ($p<0.05$) and kidney ($p<0.05$); significantly

						reduced CAT activity in liver (p<0.05) and brain (p<0.05); NPSH normalized in liver (p<0.05), kidney (p<0.05), and brain (p<0.05)
Dos Santos et al. 2018. [223]	Brazil	STZ	Wistar Rats	Other Plant Extract	Yacon leaf extract (Smallanthus sonchifolius) (YLE)	• YLE significantly reduced glycemia and increased serum insulin concentration (p<0.05)<br>• YLE significantly increased CAT (p=0.018), SOD (pM0.001), and GPx activities (p=0.047) and significantly decreased MDA (p=0.016)
Dzydzan et al. 2019. [201]	Ukraine	STZ	Wistar Rats	Fruit & Berry Polyphenol Extract	Cornelian cherry fruits (red and yellow)	• Cornelian cherry extract (red fruit) significantly decreased fasting blood glucose (p<0.05), significantly decreased OGTT AUCglc (p<0.05), and significantly decreased HbA1C (p<0.05); Cornelian cherry extract (yellow fruit) significantly decreased fasting blood glucose (p<0.05), significantly decreased OGTT AUCglc (p<0.05) and did not significantly affect HbA1c<br>• Cornelian cherry extract (red fruit) significantly reduced TBARS (p<0.05), significantly reduced oxidatively modified proteins (p<0.05), significantly reduced AGEs (p<0.05), and significantly reduced AOPPs (p<0.05); Cornelian cherry extract (yellow fruit) significantly reduced AGEs (p<0.05), significantly reduced AOPPs (p<0.05), and significantly increased reduced glutathione

						(p<0.05)
Fachinan et al. 2017. [182]	Benin	STZ	Wistar Rats	Flavonoid-Rich Plant Extract	Momordica charantia fruit juice extract (flavonoids, tannins, coumarins)	• Momordica charantia fruit juice induced significant decrease in blood glucose of T1D rats (p<0.05)<br>• No Oxidative Stress outcomes reported
Farid et al. 2022. [194]	Egypt	STZ	Sprague-Dawley Rats	Fruit & Berry Polyphenol Extract	Grape seed extract (GSE)	• GSE/MSCs co-treatment significantly decreased FBG and fructosamine (p<0.05); significantly increased serum insulin (p<0.05); GSE alone or MSCs alone showed partial improvement<br>• GSE/MSCs co-treatment significantly reduced serum MDA (p<0.05); increased GPx and SOD (p<0.05); significantly reduced pancreatic MDA (p<0.05); significantly increased GSH (p<0.05)
Figueiredo et al. 2016. [232]	Brazil	NOD	NOD Mice	Other Plant Extract	Passiflora alata aqueous leaf extract	• P. alata significantly decreased insulin (p = 0.01); significantly decreased glucose AUC (p < 0.0001)<br>• P. alata significantly increased FRAP (p < 0.0001); significantly decreased TBARS (p<0.001)
Hou et al. 2011. [186]	China	STZ	Sprague-Dawley Rats	Flavonoid-Rich Plant Extract	Flavonoid-rich fraction of Lithocarpus polystachyus Rehd. leaves (ST-3)	• ST-3 significantly reduced fasting serum insulin (p<0.05); significantly increased fasting blood glucose (p<0.05)<br>• No Oxidative Stress outcomes reported
Huo et al. 2021. [197]	China	STZ	C57BL/6 Mice	Fruit & Berry Polyphenol Extract	Fu-Pen-Zi (Chinese raspberry) polyphenol-enriched extract (FPZ)	• FPZ significantly reduced fasting blood glucose(p<0.05); significantly increased fasting insulin (p<0.05); significantly increased HOMA-β

						index (p<0.05); significantly improved glucose tolerance (p<0.05)<br>• FPZ significantly increased hepatic SOD content (p<0.05); significantly reduced total superoxide (p<0.05); significantly reduced ROS generation (p<0.05); significantly reduced hepatic MDA (p<0.05)
Kang et al. 2010. [213]	South Korea	STZ	Sprague-Dawley Rats	Phenolic Acid & Tannin-Rich Extract	Ecklonia cava methanol extract (ECM)	• ECM significantly lowered blood glucose after 3 weeks of treatment (p<0.01); significantly increased blood insulin at the end of treatment (p<0.01)<br>• No Oxidative Stress outcomes reported
Kompoura et al. 2023. [202]	Greece	STZ	Wistar Rats	Fruit & Berry Polyphenol Extract	Corinthian currants	• Corinthian currants significantly lowered blood insulin (p<0.001); did not significantly change blood glucose<br>• No Oxidative Stress outcomes reported
Kulkarni et al. 2015. [226]	India	STZ	Sprague-Dawley Rats	Other Plant Extract	Bauhinia variegata alcoholic leaf extract (AlcE)	• AlcE significantly decreased blood glucose in a dose-dependent manner (p<0.01 - 0.001); significantly decreased blood glucose in response to OGTT (p<0.001)<br>• No Oxidative Stress outcomes reported
Lafraxo et al. 2021. [231]	Morocco	Alloxan	Wistar Rats	Other Plant Extract	Thyme honey (TH) and olive oil (OO)	• TH and OO both significantly decreased blood glucose (p<0.001).<br>• Treatment with TH, OO, or combination significantly restored CAT, GSH, GPx toward normal and reduced MDA (p<0.001 for most comparisons across all three organs).

Lin et al. 2023. [199]	Taiwan	Cyclophosphamide	NOD/ShiLtJNarl Mice	Fruit & Berry Polyphenol Extract	Phyllanthus emblica L ethyl acetate extract (EPE)	• EPE significantly decreased blood glucose ($p<0.001$); significantly decreased HbA1c ($p<0.001$); significantly increased blood insulin ($p<0.001$)<br>• No Oxidative Stress outcomes reported
Madić et al. 2021. [187]	Serbia	Alloxan	Wistar Rats	Flavonoid-Rich Plant Extract	Polyherbal mixture	• D-10 significantly reduced blood glucose to near-normoglycemic levels by day 14 ($p<0.05$); D-20 significantly reduced blood glucose to normoglycemic levels by day 7 ($p<0.05$) and day 14 ($p<0.05$); both concentrations significantly more effective than insulin or metformin ($p<0.05$)<br>• No Oxidative Stress outcomes reported
Malardé et al. 2013. [188]	France	STZ	Wistar Rats	Flavonoid-Rich Plant Extract	Fermented Soy Permeate (FSP)	• FSP significantly restored muscle glucose content ($p<0.01$); did not significantly lower blood glucose<br>• No Oxidative Stress outcomes reported
Malardé et al. 2013. [189]	France	STZ	Wistar Rats	Flavonoid-Rich Plant Extract	Fermented Soy Permeate (FSP)	• FSP had no significant effect on blood glucose or plasma fructosamine<br>• FSP significantly normalized reduced SOD activity ($p<0.05$); significantly normalized reduced GPX activity ($p<0.05$); FSP significantly reduced plasma CML levels ($p<0.01$); tended to increase Mn-SOD content but not significantly; isoprostanes and GSH/GSSG ratio were not significantly different between groups

Mohammed Yusof et al. 2018. [174]	Malaysia	STZ	Sprague-Dawley Rats	Flavonoid-Rich Plant Extract	Hibiscus sabdariffa polyphenol-rich extract (HPE)	• HPE significantly decreased plasma glucose (p<0.05)<br>• HPE significantly reduced cardiac MDA and AOPP (both p<0.05; significantly increased SOD-1, SOD-2, CAT and GSH activity/levels (all p<0.05) increased GSH level
Mohammed Yusr et al. 2025. [173]	Malysia	STZ	Sprague-Dawley Rats	Flavonoid-Rich Plant Extract	Hibiscus sabdariffa polyphenol-rich extract (HPE)	• HPE significantly reduced plasma glucose (p<0.05)<br>• HPE significantly increased CAT activity (p<.05); significantly reduced MDA levels (p<0.05); HPE reduced AOPP levels (p<0.05); did not significantly increase SOD activity; did not significantly affect GSH levels;
Park et al. 2013. [228]	South Korea	STZ	ICR Mice	Other Plant Extract	Olive leaf powder	• Olive leaf powder at very low and low doses showed significantly reduced fasting blood glucose (p<0.05); insulin levels were significantly increased in all dose groups(p<0.05)<br>• Olive leaf powder at very low and low doses significantly increased SOD and CAT activity (both p<0.05); significantly increased GPx (p<0.05 and p<0.001 respectively); significantly decreased iNOS mRNA expression in the very low group (p<0.05); did not significantly effect NO levels
Patel et al. 2011. [200]	India	STZ	Wistar Rats	Fruit & Berry Polyphenol Extract	Emblica officinalis fruit juice	• E. officinalis fruit juice significantly reduced serum glucose (p<0.05); significantly reduced AUC glucose (p<0.05); did not significantly increase serum insulin

						• E. officinalis fruit juice significantly increased SOD, CAT, GSH (all p<0.05); significantly decreased MDA (p<0.05)
Patel et al. 2011. [183]	India	STZ	Wistar Rats	Flavonoid-Rich Plant Extract	Alcoholic extract of Hybanthus enneaspermus (AHE)	• AHE significantly reduced fasting plasma glucose on days 7, 14, and 21 (p<0.05)<br>• No Oxidative Stress outcomes reported
Qi et al. 2022. [175]	China	STZ	C57BL/6 Mice	Flavonoid-Rich Plant Extract	Total flavonoids of Sedum aizoon L. (STF)	• STF significantly reduced FBG (p<0.05); significantly improved OGTT glucose levels and reduced AUC (p<0.05)<br>• STF significantly increased T-SOD activity (p<0.05); significantly increased GSH levels (p<0.05); significantly increased CAT activity (p<0.05); significantly reduced MDA content (p<0.05); significantly upregulated HO-1 and NQO1 protein expression and Nrf2 nuclear expression, and reduced Keap1 expression (p<0.05)
Rahim Pouran et al. 2020. [181]	Malaysia, South Korea	Alloxan	Wistar Rats	Flavonoid-Rich Plant Extract	Milk thistle seed extract (MTSE; silymarin-rich), MTSE/ZnO/Ag nanocomposite	• MTSE/ZnO/Ag significantly decreased fasting blood glucose (p<0.05); significantly increased plasma insulin (p<0.05)<br>• No Oxidative Stress outcomes reported
Ramachandran et al. 2011. [176]	India	STZ	Wistar Rats	Flavonoid-Rich Plant Extract	Ethanolic extract of Sphaeranthus indicus root (EESIR)	• EESIR 100 and 200 mg/kg significantly reduced blood glucose (p<0.01); EESIR 200 mg/kg produced significantly greater reduction than 100 mg/kg (p<0.01)<br>• EESIR 100 and 200 mg/kg significantly increased SOD and CAT (p<0.01); 200 mg/kg significantly increased SOD vs.

						100 mg/kg (p<0.01); 100 and 200 mg/kg significantly increased GPx (p<0.05); 100 and 200 mg/kg significantly reduced TBARS(p<0.01)
Sameni et al. 2016. [180]	Iran	STZ	Wistar Rats	Flavonoid-Rich Plant Extract	Propolis ethanolic extract (Iranian propolis) (EEP)	• Propolis extract at both doses significantly reduced serum glucose levels (p<0.001)<br>• Propolis extract significantly reduced MDA content and increased SOD and GPx activity in kidney tissue (p<0.001); significantly increased total antioxidant activity (FRAP) (p<0.001)
Sanna et al. 2019. [195]	India	STZ	Wistar Rats	Fruit & Berry Polyphenol Extract	Grape seed proanthocyanidin extract (GSPE)	• GSPE significantly reduced FBS and PPBS (p<0.05); significantly improved OGTT (p<0.05); significantly improved fasting plasma insulin (p<0.001)<br>• No Oxidative Stress outcomes reported
Schumacher et al. 2015. [214]	Brazil	NOD	NOD Mice	Phenolic Acid & Tannin-Rich Extract	Eugenia uniflora L. aqueous leaf extract	• Treatment significantly increased serum insulin (p<0.05); significantly lowered blood glucose and GTT AUC (p<0.001)<br>• Treatment significantly increased hepatic GSH (p<0.05); significantly decreased serum TBARS (p<0.01)
Kiran et al. 2019. [210]	India	STZ	Wistar Rats	Phenolic Acid & Tannin-Rich Extract	Excoecaria agallocha L. ethanolic leaf extract (EAL)	• EAL 250 mg/kg and 500 mg/kg significantly decreased blood glucose (p<0.05)<br>• EAL significantly increased SOD, CAT and GSH (p<0.05); significantly decreased MDA (p<0.05)

Shivanna et al. 2013. [227]	India	STZ	Wistar Rats	Other Plant Extract	Stevia Leaves	• Stevia polyphenols significantly decreased blood glucose (p<0.05) and significantly increased serum insulin (p<0.05); significantly improved IPGTT blood glucose at 90 min (p<0.05) and significantly improved IPITT blood glucose at 15, 45, and 90 min (p<0.05)<br>• Stevia polyphenols significantly reduced liver hydroperoxides and significantly reduced liver conjugated dienes (both p<0.05); significantly increased liver SOD and catalase (both p<0.05); did not significantly affect liver TBARS
Shu et al. 2009. [190]	China	STZ	Kunming Mice, Sprague-Dawley Rats	Flavonoid-Rich Plant Extract	Total Flavonoids of Polygonatum odoratum (TFP)	• TFP significantly reduced fasting blood at 100 and 200 mg/kg by day 6 and at 50, 100, and 200 mg/kg by day 9 (p<0.05-p<0.001); TFP did not significantly impact insulin levels<br>• No Oxidative Stress outcomes reported
Ştefănescu Braic et al. 2018. [205]	Romania	STZ	Wistar Rats	Fruit & Berry Polyphenol Extract	Vaccinium myrtillus leaf/fruit extract (VML/VMCLF), Vaccinium corymbosum leaf/fruit extract (VCL/VCLF)	• VML, VMLF, and VCLF groups showed decreased blood glucose during 8-week experiment, but not significantly; DC, PC, and VCL groups showed no improvement<br>• Vaccinium extracts did not significantly impact MDA levels
Tedong et al. 2006. [233]	Cameroon	STZ	Wistar Rats	Other Plant Extract	Hexane Extract of Anacardium occidentale	• Extract at 300 mg/kg/day significantly decreased fasting blood glucose, significantly reduced total urinary protein, significantly reduced albuminuria,

						significantly reduced glycosuria, and significantly reduced urinary urea (all p<0.05); 150 mg/kg/day significantly reduced glycosuria but did not significantly reduce urinary urea<br>• No Oxidative Stress outcomes reported
Usatiuc et al. 2025. [211]	Romania	STZ	Wistar Rats	Phenolic Acid & Tannin-Rich Extract	Lythrum salicaria L. ethanol extract (LSEE)	• LSEE 50% significantly reduced blood glucose (p<0.001); 100% and 25% caused significant reduction (p<0.01); 100% effect was better than metformin (p<0.05)<br>• LSEE at all three concentration significantly reduced TOS, OSI, MDA, AOPP and 3-NT (all p<0.001); reduced 8-OHdG (p<0.01 at 100% and 25%, p<0.001 at 50%); significantly increased SH (p<0.001); significantly increased TAC (p<0.01 at 100%, p<0.05 at 25%); did not significantly impact NOx
Usatiuc et al. 2025. [217]	Romania	STZ	Wistar Rats	Phenolic Acid & Tannin-Rich Extract	Plantago ovata ethanol extract (POEE)	• POEE100% significantly reduced blood glucose (p<0.01), comparable to metformin (p<0.01); POEE50% and POEE25% showed non-significant reductions<br>• POEE100% and 50% significantly reduced TOS and OSI (both p<0.001); POEE25% showed smaller reduction in TOS and OSI (p<0.05); all three POEE concentrations significantly reduced MDA (p<0.001); POEE100% and 50% significantly reduced AOPP (p<0.001);

						POEE100% and 50% significantly reduced 8-OHdG (p<0.01); POEE100% significantly reduced AGEs (p<0.05); POEE100% and 50% significantly reduced NOx (p<0.01); all three POEE concentrations significantly reduced 3NT (p<0.001); all three POEE concentrations significantly increased TAC (p<0.001); POEE100% and 50% significantly increased SH (p<0.001)
Veerapur et al. 2017. [177]	India	STZ	Wistar Rats	Flavonoid-Rich Plant Extract	Cassia glauca polyphenolic extract (CGE)	• CGE (400 mg/kg) significantly reduced serum glucose (p<0.001) at day 15; significantly improved OGTT AUC glucose (p<0.001); significantly increased AUC insulin (p<0.001); significantly reduced AUC glucose (p<0.001)<br>• CGE (200 and 400 mg/kg) significantly increased GSH levels (p<0.001); significantly increased total thiols (p<0.001); significantly reduced TBARS levels (p<0.001); significantly increased catalase activity (p<0.05)
Wang et al. 2025. [203]	Taiwan	STZ	Sprague-Dawley Rats	Fruit & Berry Polyphenol Extract	Apple Polyphenols	• Apple polyphenol did not significantly affect blood glucose<br>• Apple polyphenol significantly reduced kidney TBARS (MDA) at 1% and 2% (p<0.05); significantly reduced kidney GST-p at 0.5% and 2% (p<0.05); did not significantly affect kidney GST or GST-a (p>0.05)

Xu et al. 2024. [230]	China	STZ	C57BL/6 Mice	Other Plant Extract	Si Wei Jiang Huang Tang San (SWJHTS)	• SWJHTS significantly reduced FBG at day 7 ($p<0.05$); significantly reduced OGTT AUC ($p<0.05$)<br>• No Oxidative Stress outcomes reported
Yadav et al. 2025. [215]	India	STZ	Sprague-Dawley Rats	Phenolic Acid & Tannin-Rich Extract	Aqueous seed extract of Syzygium cumini (AESC)	• AESC significantly reduced fasting blood glucose levels($p<0.001$);<br>• AESC significantly restored SOD activity ($p<0.001$); significantly restored CAT activity ($p<0.001$); significantly restored GSH levels ($p<0.001$); significantly reduced MDA levels ($p<0.001$)
Yin et al. 2018. [234]	China	Alloxan	Sprague-Dawley Rats	Other Plant Extract	Ethanol crude extract (ECE)	• ECE at 800 mg/kg significantly reduced FBG from day 2 to day 7 ($p<0.05$) and extremely significantly from day 10 ($p<0.01$); ECE at 200 mg/kg did not significantly reduce FBG<br>• ECE at 800 mg/kg significantly decreased MDA in heart tissue ($p<0.05$); at both doses significantly recovered SOD activities in heart, kidney, and spleen ($p<0.05$) and extremely significantly in liver ($p<0.01$); significantly reduced GSH levels ($p<0.01$)
Ziyanok-Demirtas et al. 2024. [222]	Turkey	STZ	Wistar Rats	Other Plant Extract	Hibiscus trionum tea (HTT)	• HTT significantly reduced blood glucose ($p<0.05$); HTT significantly increased serum insulin ($p<0.05$)<br>• HTT significantly increased plasma SOD ($p<0.01$); significantly increased muscle and kidney SOD ($p<0.05$); significantly increased liver SOD ($p<0.05$); significantly increased plasma GSH-Px ($p<0.05$); significantly

						increased muscle and kidney GSH-Px ($p<0.05$); significantly reduced plasma, heart, skeletal muscle, liver, and kidney MDA ($p<0.05$)
Zunino et al. 2007. [196]	USA	NOD	NOD Mice	Fruit & Berry Polyphenol Extract	Grape polyphenols (freeze-dried grape powder)	• Grape polyphenols significantly reduced diabetes incidence ($p<0.05$) • No Oxidative Stress outcomes reported
