# Supplementary material for: Polyphenol-Mediated Modulation of Oxidative Stress Pathways in Type 1 Diabetes: A Systematic Review
Source: Antioxidants (Basel). 2026 May 30;15(6):693. doi: 10.3390/antiox15060693 (PMC13295557; doi:10.3390/antiox15060693)
Supplement: Supplementary file 1 [file antioxidants-15-00693-s001.zip › Supplementary Table 5.pdf]

Supplementary Table 5: A summary of included other polyphenol studies

Reference	Country	T1D Induction Method	Animal Model	Polyphenol Subclass	Polyphenol Investigated	Outcomes Summarized
Abdulhadi et al. 2022. [251]	Iraq, Egypt	STZ	Wistar Rats	Ellagitannin	Punicalagin	• Punicalagin significantly decreased blood glucose (p<0.001); significantly increased serum insulin (p<0.001)<br>• Punicalagin significantly reduced pancreatic MDA and protein carbonyl (p<0.001); significantly increased pancreatic GSH, GPx, GR, SOD, CAT (p<0.001); significantly increased serum PON-1 activity (p<0.001)
Al-Hussan et al. 2024. [239]	Saudi Arabia	STZ	Wistar Rats	Chalcones and Dihydrochalcones	Phloretamide	• Phloretamide significantly reduced fasting plasma glucose (p<0.05) and insulin (p<0.05)<br>• Phloretamide significantly reduced renal MDA (p<0.05) and AGEs (p<0.05); significantly increased renal GSH, SOD , and HO-1 (all p<0.05)
Altamimi et al. 2022. [250]	Saudi Arabia	STZ	Wistar Rats	Ellagitannin	Ellagic Acid (EA)	• EA significantly reduced fasting glucose (p<0.05); significantly increased fasting insulin (p<0.05)<br>• EA significantly reduced hepatic ROS, MDA (both p<0.05); significantly increased hepatic GSH and SOD (both p<0.05); significantly increased nuclear Nrf2 activity (p<0.05)

Fang et al. 2015. [241]	China	STZ	C57BL/6 Mice	Chalcones and Dihydrochalcones	L2H17 (1-(3,4-dihydroxyphenyl)-3-(2-methoxyphenyl)prop-2-en-1-one) (synthetically derived Chalcone)	• L2H17 did not significantly affect blood glucose levels<br>• No Oxidative Stress outcomes reported
Huang et al. 2020. [242]	China	STZ	C57BL/6 Mice	Chalcones and Dihydrochalcones	Isoliquiritigenin (ISL)	• No Glycemic Control outcomes reported<br>• ISL significantly prevented HG-induced decrease of SOD activity in kidney tissues ($p<0.05$); significantly reduced ROS (O_2^-) production in renal tissues as shown by DHE staining ($p<0.001$); significantly increased Nrf2 mRNA and protein levels ($p<0.001$); significantly increased HO-1 mRNA levels ($p<0.001$)
Lee et al. 2024. [244]	Taiwan	STZ	Sprague-Dawley Rats	Phenolic Acid	Syringaldehyde (SA)	• SA significantly decreased blood glucose ($p<0.01$) and increased serum insulin ($p<0.05$)<br>• No Oxidative Stress outcomes reported
Li et al. 2020. [255]	China	STZ	C57BL/6 Mice	Lignan	Syringaresinol (SYR)	• SYR did not significantly affect blood glucose, or glucose tolerance levels<br>• SYR significantly reduced ROS generation in diabetic myocardium ($p<0.05$); significantly increased Nrf2 mRNA and protein expression ($p<0.05$); significantly increased SOD protein expression ($p<0.05$); significantly increased HO-1 mRNA ($p<0.05$); significantly down-

						regulated Keap1 protein ($p<0.05$); significantly increased NQO-1 mRNA ($p<0.05$)
Liu et al. 2024. [253]	China	STZ	C57BL/6 Mice	Lignan	Syringaresinol (SYR)	• SYR treatment attenuated fasting blood glucose elevation ($p<0.05$)<br>• SYR significantly reduced retinal ROS generation and serum ROS and H_2O_2($p<0.001$); significantly upregulated Nrf2 protein and mRNA ($p<0.05$), HO-1 ($p<0.0001$), and SOD2 ($p<0.0001$)
Najafian et al. 2010. [238]	Iran	STZ	Wistar Rats	Chalcones and Dihydrochalcones	Trans-chalcone	• trans-chalcone significantly reduced blood glucose levels (p not reported) and serum insulin ($p<0.01$)<br>• No Oxidative Stress outcomes reported
Rodriguez-Pérez et al. 2023. [246]	Spain	STZ	Wistar Rats	Phenolic Acid	3',4'-Dihydroxyphenylglycol (DHPG)	• DHPG significantly reduced blood glucose at both doses ($p<0.05$)<br>• DHPG significantly reduced serum and kidney TBARS ($p<0.05$); significantly reduced 8-OHdG in serum and kidney ($p<0.05$); significantly reduced oxLDL in serum ($p<0.05$); significantly increased GSH, TAC, 3-nitrotyrosine in serum and kidney (all $p<0.05$); significantly reduced urinary 8-isoprostane ($p<0.05$)
Semaming et al. 2014. [247]	Thailand	STZ	Sprague-Dawley Rats	Phenolic Acid	Protocatechuic acid (PCA)	• PCA 50 and 100 mg/kg significantly decreased FBG ($p<0.05$); significantly decreased plasma HbA1c ($p<0.05$); did

						not significantly affect plasma insulin<br>• PCA at 50 and 100 mg/kg significantly decreased cardiac MDA at week 12 (p<0.05); significantly decreased cardiac mitochondrial ROS production at both doses (p<0.05)
Shelke et al. 2023. [240]	India	STZ	Wistar Rats	Chalcones and Dihydrochalcones	Phloretin	• Phloretin significantly lowered blood glucose (p<0.05)<br>• No Oxidative Stress outcomes reported
Sorrenti et al. 2019. [245]	Italy	STZ	Wistar Rats	Phenolic Acid	Caffeic acid phenethyl ester (CAPE), VP961 (CAPE analogue)	• CAPE- and VP961-significantly reduced blood glucose at 8, 15, and 21 days (p<0.05); significantly increased plasma insulin (p<0.05)<br>• CAPE- and VP961-significantly reduced plasma lipid hydroperoxide (p<0.05); significantly reduced pancreatic lipid hydroperoxide (p<0.05); significantly increased plasma RSH (p<0.05); significantly upregulated HO-1 and GGCL protein expression (p<0.05)
